# Discovery, Semisynthesis, Antiparasitic and Cytotoxic Evaluation of 14-Membered Resorcylic Acid Lactones and Their Derivatives

**DOI:** 10.1038/s41598-017-12336-0

**Published:** 2017-09-18

**Authors:** Xue-Qing Zhang, Carmenza Spadafora, Laura M. Pineda, Michelle G. Ng, Ji-Hong Sun, Wei Wang, Chang-Yun Wang, Yu-Cheng Gu, Chang-Lun Shao

**Affiliations:** 1 Key Laboratory of Marine Drugs, The Ministry of Education of China, School of Medicine and Pharmacy, Ocean University of China, Qingdao, 266003 People’s Republic of China; 2 Laboratory for Marine Drugs and Bioproducts, Qingdao National Laboratory for Marine Science and Technology, Qingdao, 266200 People’s Republic of China; 3 Center of Cellular and Molecular Biology of Diseases, Instituto de Investigaciones Científicas y Servicios de Alta Tecnología, City of Knowledge, Clayton, Apartado 0816-02852 Panama; 4 Syngenta Jealott’s Hill International Research Centre, Bracknell, Berkshire, RG42 6EY United Kingdom

## Abstract

Ten antifouling 14-membered resorcylic acid lactones **1**–**10** were isolated previously with low or trace natural abundance from the zoanthid-derived *Cochliobolus lunatus* fungus. Further optimization of fermentation conditions led to the isolation of two major natural compounds **7** and **8** with multi-gram quantities. By one or two steps, we semisynthesized the six trace natural compounds **1**–**6** and a series of derivatives **11**–**27** of compounds **7** and **8** with high yields (65–95%). Compounds **11**–**13** showed strong antiplasmodial activity against *Plasmodium falciparum* with IC_50_ values of 1.84, 8.36, and 6.95 *μ*M, respectively. Very importantly, **11** and **12** were non-toxic with very safety and high therapeutic indices (CC_50_/IC_50_ > 180), and thus representing potential promising leads for antiplasmodial drug discovery. Furthermore, **11** was the only compound showed obvious antileishmanial activity against *Leishmania donovani* with an IC_50_ value of 9.22 *μ*M. Compounds **11** and **12** showed the values of IC_50_ at 11.9 and 17.2 *μ*M against neglected Chagas’ disease causing *Trypanosoma cruzi*, respectively.

## Introduction

Protozoan infections such as malaria, leishmaniasis, and Chagas’ disease remain a major threat to public health especially in the tropical regions of the world. Malaria is a life-threatening mosquito-borne infectious disease caused by a parasite of the genus *Plasmodium*. According to the World Health Organization, there are 212 million new cases of malaria with 429,000 deaths in 2015. Malaria is a serious problem especially in Africa, where it is responsible for most global malaria cases (90%)^1^. Leishmaniasis is a debilitating and devastating disease caused by protozoan parasites of the genus *Leishmania*, which is endemic in 98 countries, with 20,000–30,000 deaths and 0.7–1.0 million new cases annually^2^. Chagas’ disease or American trypanosomiasis is caused by the parasite *Trypanosoma cruzi* and is responsible for about 5.7 million people infected with the parasite and 7,000 deaths per year^3,4^. Current drugs used for the treatment of these diseases are effective, but resistance and side effects, in some cases serious, have been reported for all the current therapeutic agents^5–8^. There is therefore an urgent need to develop new antimalarial, antileishmanial, and antitrypanosomal agents that act on new targets with novel modes of action.

Fungi have been proven to be a rich source of structurally diverse and biologically active secondary metabolites that are of considerable synthetic interest and dramatic significance as promising leads for drug discovery and development^9,10^. 14-Membered resorcylic acid lactones (RALs) constitute a class of fungal polyketide metabolites which exhibit a wide range of biological properties including antimalarial^11,12^, cytotoxic^13^, antiparasitic, antiviral^14^, and kinase inhibitory^15,16^ activities. Paecilomycins E and F, with potent antiplasmodial activity against *P. falciparum* lines 3D7 (IC_50_: 20 nM and 1.1 *μ*M) and Dd2 (IC_50_: 8.8 *μ*M and 1.7 *μ*M, respectively), were isolated from a *Paecilomyces* fungus^11,12^. Hypothemycin and aigialomycin D that exhibited antimalarial activity against *P. falciparum* lines K1 with IC_50_ values of 2.2 and 6.6 *μ*g/mL respectively and cytotoxicity, were isolated from *Aigialus parvus* ^13^. In our previous studies, cochliomycins A, C–F (**1**–**5**), and several analogues (**6**–**10**) have been isolated with low or trace yields from the fungus *C. lunatus* (M351)^17^ and *C. lunatus* (TA26-46)^18^. In view of their potentially biological applications, cochliomycins have attracted the attention of academic groups to synthesize these compounds. The most challenging part of their syntheses was the assembly of the macrolide ring and a significant range of techniques with complicated procedures have been tried and improved^19–25^. For example, cochliomycin A was firstly synthesized with 22 steps (6.5% overall yields) employing stereo-selective Keck allylation, Juliae–Kocienski olefination and a late-stage RCM reaction as the key steps^19^. Meanwhile, the scarce availability of the natural products including time-consuming isolation protocols has also prompted our study of alternative sources of these materials for additional studies.

Up to now, total syntheses of cochliomycins A–C have been accomplished extensively by Nanda’s group^19,24^, Du’s group^20,21^, Srihari’s group^23^, and Banwell’s group^22,25^. Cochliomycin A (**1**) was firstly synthesized in overall yield of 6.5% in 2012^19^, and then was stereo-selectively synthesized by Wang *et al*. with 16 steps and only mg scale (23.0 mg)^20^. Cochliomycin B has been synthesized in 4.8% overall yields^21^ and the latest progress in the modular total syntheses of **1** (36.8 mg) and cochliomycin B (58.0 mg) were reported with 18 steps^22^. Cochliomycins A–C were in close resemblance to antiplasmodial paecilomycins E and F structurally, and cochliomycin C (**2**) is a chlorinated derivative of paecilomycin F on aromatic ring at C5 carbon. Total syntheses of paecilomycins E and F were also accomplished and reported during the period 2012–2016^26–30^. Very recently, cochliomycin C (**2**) and paecilomycin F were synthesized successively by Srihari’s group^23^ with 16 steps, Nanda’s group^24^ with 12 steps in overall yield of 14.1%, and Banwell’s group^25^ with 11 steps. There is no report on total syntheses of compounds **3**–**6**. Alternatively, semisynthetic strategy could provide an efficient and economical route to obtain natural products for further investigation of their pharmacological activities.

**Figure 1:**
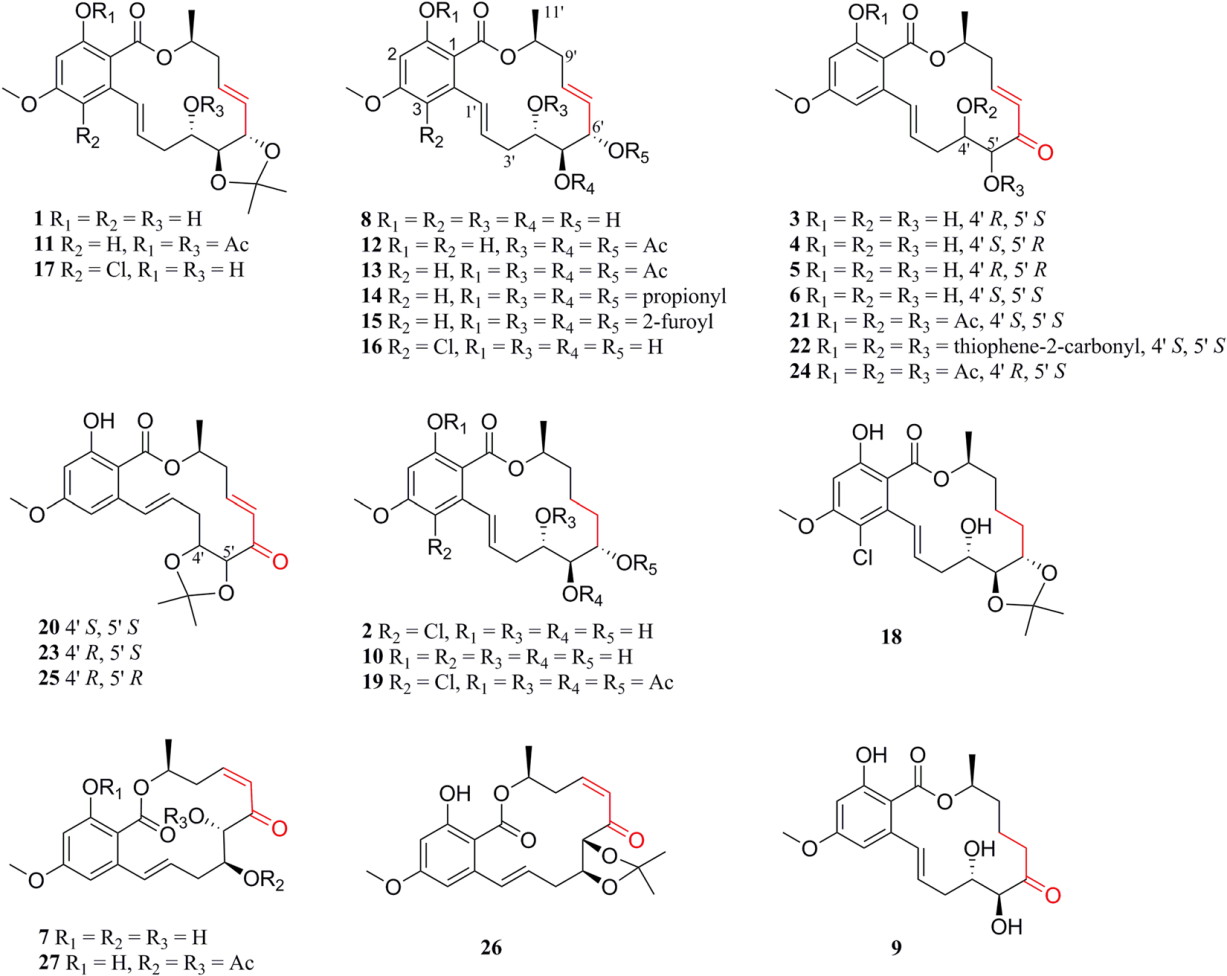
Chemical structures of natural compounds **1**–**10** and their derivatives **11**–**27**.

In this study, further optimization of the fermentation conditions of *C. lunatus* resulted in multi-gram quantities of compounds **7** and **8**, and the one or two-step semisyntheses of natural compounds **1**–**6** and a series of derivatives **11**–**27** with high yields were reported (Fig. 1). The antiplasmodial, antileishmanial, antitrypanosomal, and cytotoxic activities of these compounds were evaluated *in vitro*. The preliminary structure-activity relationships were discussed.

## Results and Discussion

### Progress of the optimization of fermentation

**Figure 2:**
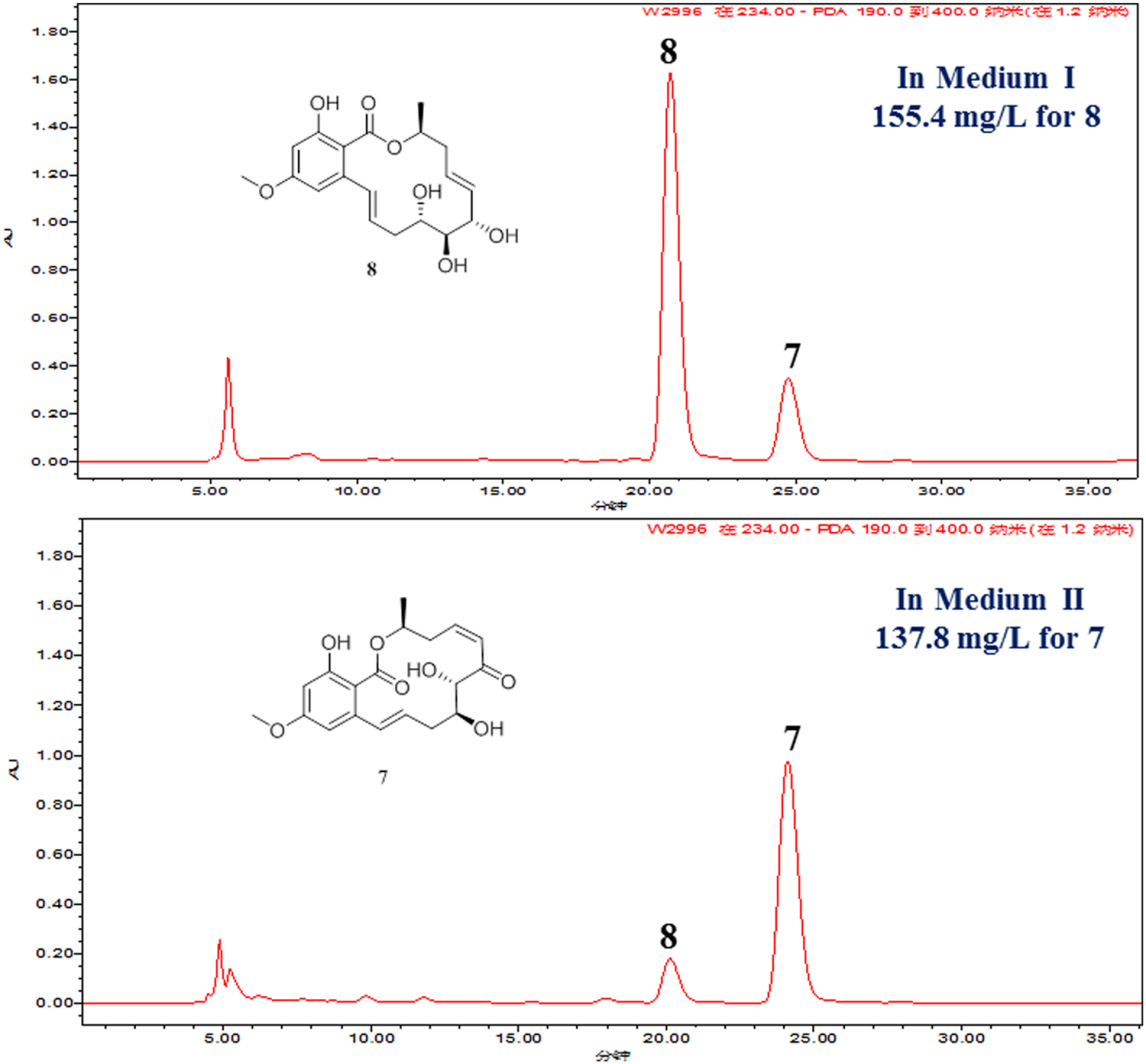
HPLC profiles for the EtOAc extract of metabolites in the media Medium I and Medium II.

The fungal strain *C. lunatus* was cultured in a 500 mL flask containing 200 mL liquid medium and incubated at 28 °C for 7 days on a rotary shaker at 120 rpm. The result of analysis showed that **7** and **8** are two predominant products in oligotrophic liquid medium (soluble starch 10 g/L, tryptone 1 g/L, 3% salinity). Then, the medium was selected as the basic medium for further optimization. The level of all the single factors including carbon source, nitrogen source, salinity, pH, inoculum size, and cultural time were studied. Subsequently, orthogonal test was carried out to approach the optimum point. All experiments were carried out in triplicate and the yields of **7** and **8** were calculated as the average values of three independent experiments. The fermented liquid medium was extracted with 200 mL ethyl acetate for each flask three times. The yield of **8** was 155.4 mg/L (soluble starch 10 g/L, NaNO_3_ 5 g/L, NaOAc 1 g/L, 1% salinity, 10% inoculum size, adjusting initial pH value to 6 with 10% HCl/NaOH, Medium I). The yield of **7** reached 137.8 mg/L (soluble starch 10 g/L, NaNO_3_ 5 g/L, 0.1% salinity, 15% inoculum size, adjusting initial pH value to 6 with 10% HCl/NaOH, Medium II) (Fig. 2). This supplied with a feasible method to obtain multi-gram quantities of **7** and **8** by fermentation in the laboratory. The advantages of the fermentation method were low cost, time saving, and easy separation by recrystallization. The structures of **7** and **8** were determined by comparing the NMR and MS spectroscopic data with that reported in literature^31,32^.

### Semisynthesis

**Figure 3:**
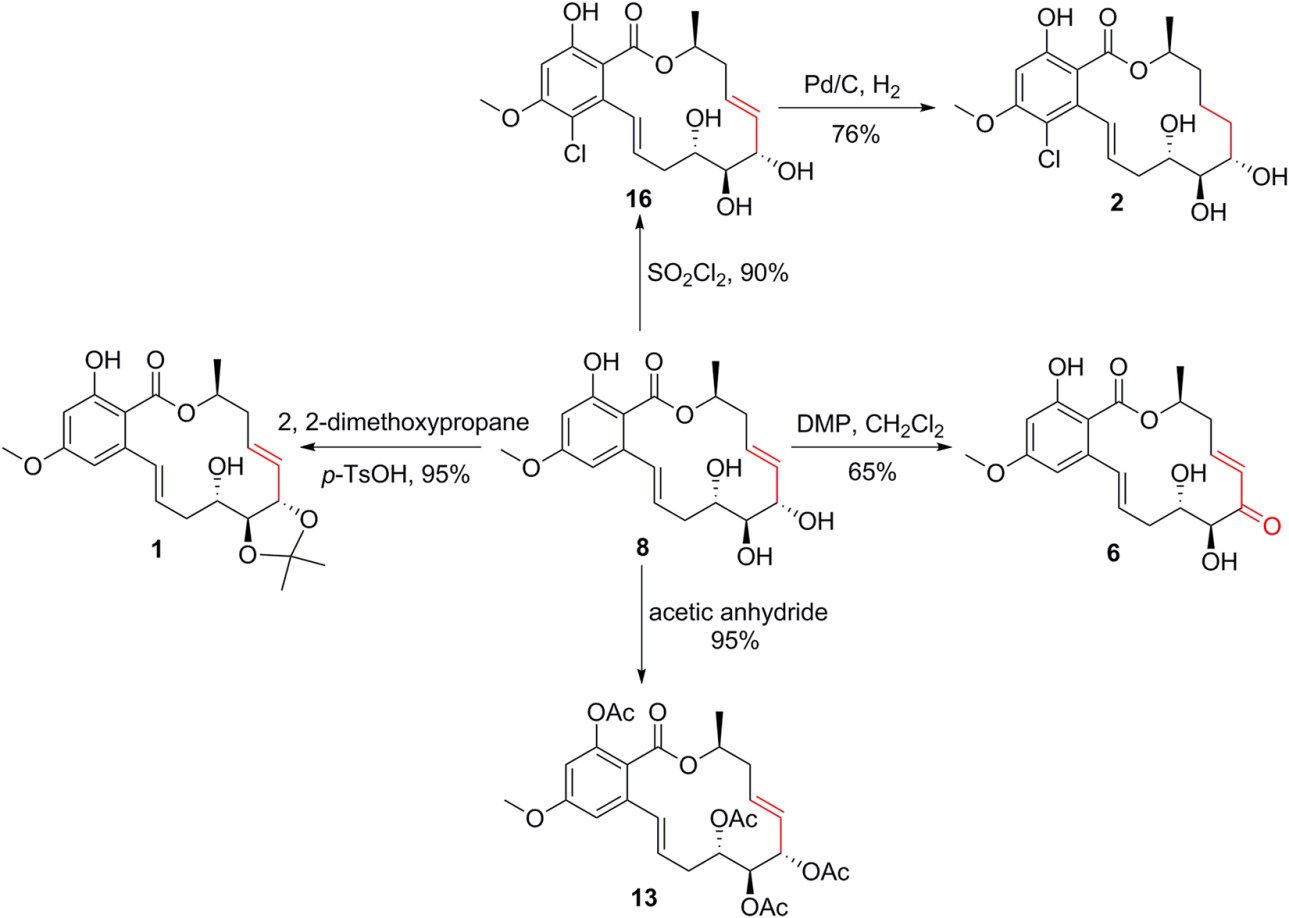
Example of one or two-step semisyntheses of compounds **1**, **2**, **6**, **13**, and **16**.

**Figure 4:**
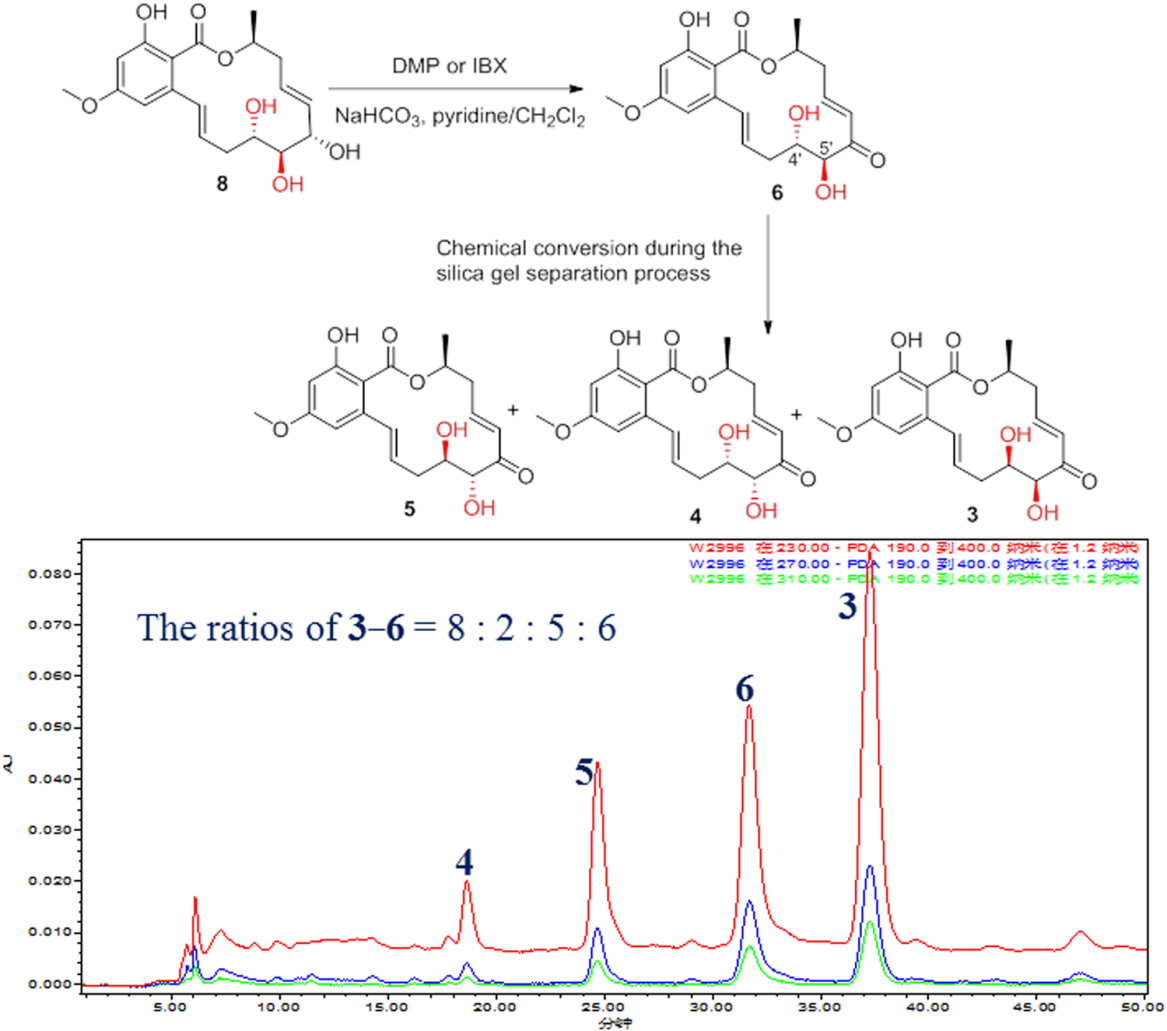
Semisynthesis of compound **6** and chemical conversion of **6** to **3**–**5**.

**Figure 5:**
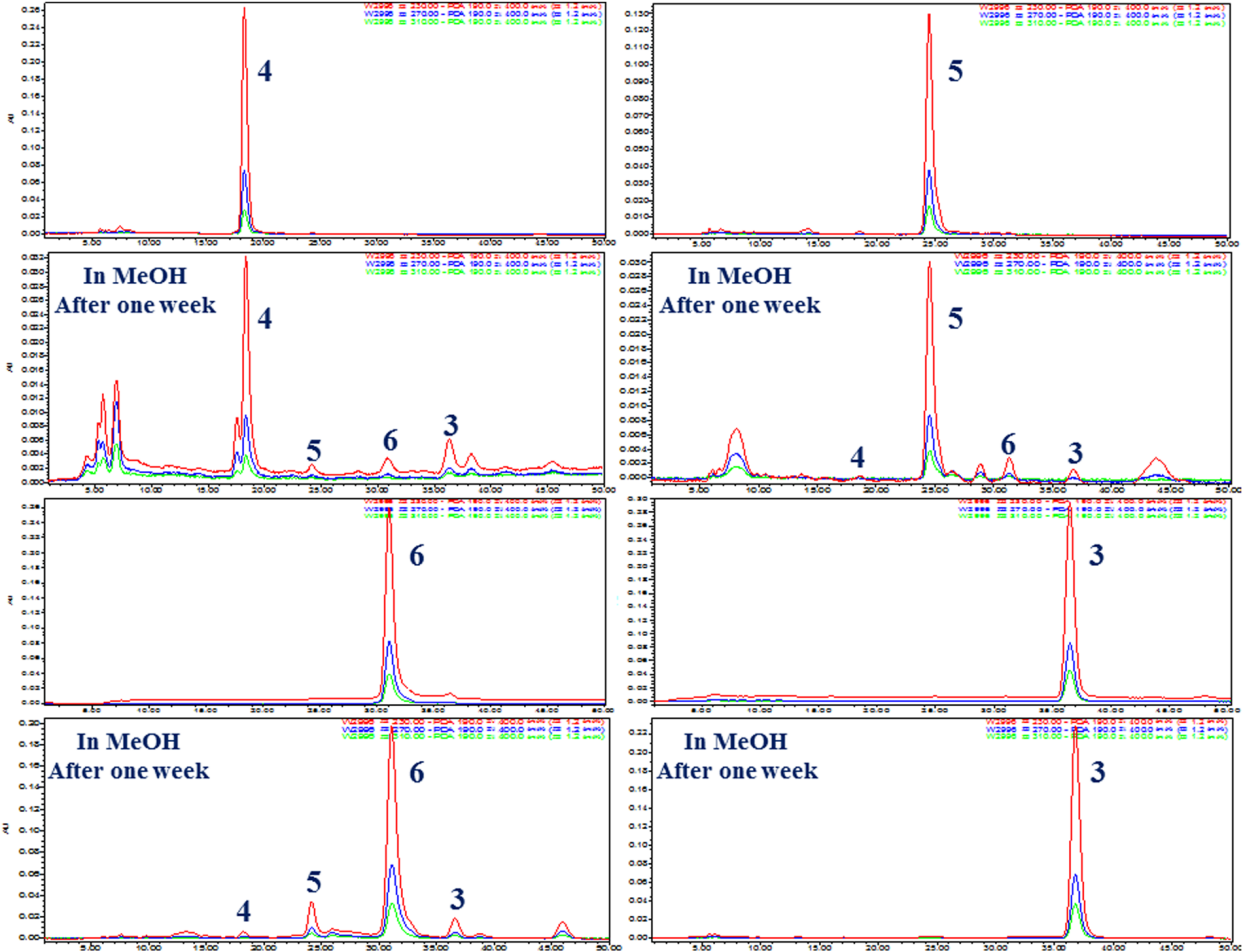
Chemical interconversion of compounds **3**–**6** in MeOH.

Compounds **1**–**6** and **11**–**27** were designed and synthesized through one or two-step semisynthetic reaction with high yields ranging from 65–95% from **7** and **8** (Fig. 1). The structures of new derivatives **12**, **14**–**19**, **21**–**22**, **24**, and **27** were identified by extensive spectroscopic methods including HRESIMS, ^1^H NMR, and ^13^C NMR. Compound **1** was prepared in yield of 95% through one-step acetonide reaction from **8**. Other acetonide derivatives (**11**, **17**, **18**, **20**, **23**, **25**, and **26**) were also prepared with the same yields. Chlorination of compound **8** with sulfuryl chloride^33^ followed by selective reduction with 10% Pd-C under H_2_ atmosphere led to the natural compound **2** (68%, yield). Acylation reaction was carried out by reaction of anhydride with the corresponding materials (**1**–**3**, and **6**–**8**) in high yields (85–95%). In order to prepare **6**, oxidation of the allyl alcohol **8** was examined under various oxidation conditions (PCC, PDC, MnO_2_, DMP, and IBX). However, only DMP and IBX enabled the selective oxidation of the allyl alcohol to give **6** in 65% and 35% yields, respectively (Fig. 3). Interestingly, a fortuitous observation revealed that **6** can slowly proceed in chemical conversion to **3**–**5** in the process of silica gel column separation. Compounds **3**–**6** are diastereomers differing from each other by the absolute configurations of the 4′, 5′-diol chiral centers. The proportions of compounds **3**–**6** were in dynamic equilibrium, and the final ratios of **3**–**6** were approximately 8: 2: 5: 6 (Fig. 4). Further study revealed that a subtle chemical conversion of **6** to **3**–**5** was observed in protic solvents (MeOH, EtOH, and MeOH-*d*_4_, except for H_2_O) in the stationary state within a week, and similar interconversion of **4** and **5** were also observed under the same conditions, while compound **3** was found to be quite stable (Fig. 5). However, compounds **4**–**6** were found to be stable in aprotic solvents (EtOAc, CH_3_CN, acetone, and CH_2_Cl_2_). Compounds **4**–**6** were unstable because of the existence of *trans*-enone moieties and the absolute configurations of the 4′, 5′-diol groups, causing chemical conversions under the condition of protic solvent, such as intramolecular hetero Michael addition^34^ and carbon migration of hemiacetal^35^. This conversion played a vital role in obtaining compounds **3**–**5** for further biological study.

### Antiplasmodial activity

**Figure 6:**
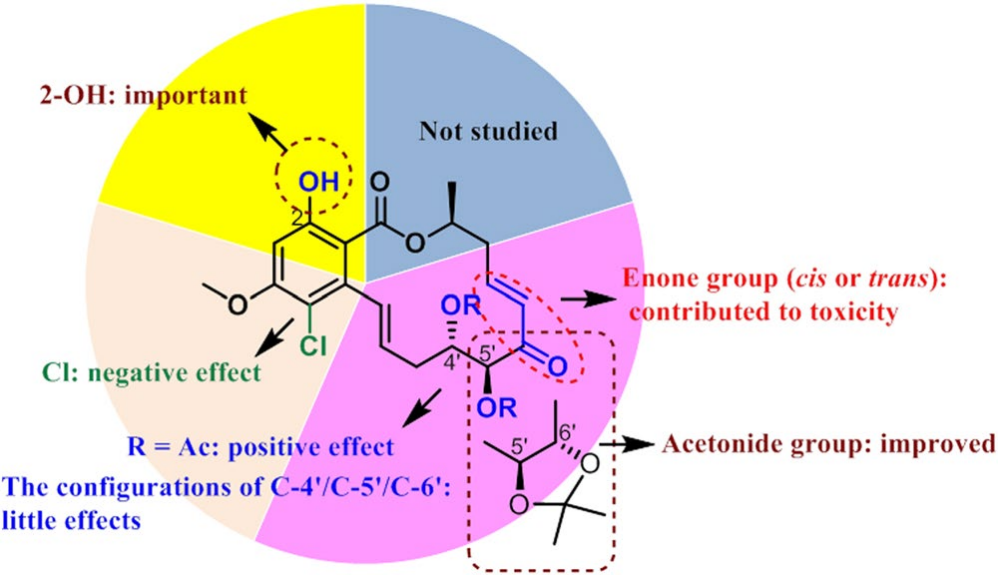
The structure-activity relationships of RALs.

Results for the *in vitro* antiplasmodial activity of all of the compounds were shown in Table 1. Compounds **11**–**13** and **26** exhibited strong antiplasmodial activity with the IC_50_ values of 1.84, 8.36, 6.95, and 8.95 *μ*M, respectively. It should be pointed that the introduction of the acetoxy groups in compounds **1** and **8** appreciably change the activity, indicating that adding the acetoxy groups has a positive effect on the antiplasmodial activity. The acetonide functionality in **11** improved the IC_50_ value approximately 4-fold over that of **13** with the acetoxy groups at C5′-C6′. This suggests that the acetonide functionality might contribute to the antiplasmodial activity. However, the introduction of the chlorine atom at C5 in **2** and **16**–**19** was all found to be inactive, indicating that the chlorine atom has a negative effect on the antiplasmodial activity (Fig. 6).

**Table 1:** Antiplasmodial, antileishmanial, antitrypanosomal, and toxic activities of compounds **1**–**27**.

No.	*Plasmodium falciparum*		*Leishmania donovani*		*Trypanosoma cruzi*		Vero cells
	IC_50_ (*μ*M)	SI	IC_50_ (*μ*M)	SI	IC_50_ (*μ*M)	SI	CC_50_ (*μ*M)
**1**	30.7	3	I	—	I	—	96.0
**2**	I	—	I	—	I	—	nt
**3**	I	—	9.11	—	I	—	nt
**4**	I	—	15.2	—	I	—	nt
**5**	I	—	I	—	I	—	nt
**6**	14.9	<1	1.93	2	nt	—	3.20
**7**	11.0	<1	2.21	<1	nt	—	<1
**8**	I	—	I	—	I	—	227.1
**9**	I	—	I	—	I	—	131.0
**10**	I	—	I	—	I	—	273.8
**11**	1.84	184	9.22	37	11.9	28	338.7
**12**	8.36	182	15.7	97	17.2	88	1520.5
**13**	6.95	61	I	—	I	—	426.6
**14**	I	—	I	—	I	—	nt
**15**	22.2	71	I	—	I	—	1567.8
**16**	I	—	I	—	I	—	260.0
**17**	I	—	I	—	I	—	143.1
**18**	I	—	I	—	I	—	101.9
**19**	I	—	I	—	I	—	nt
**20**	I	—	6.46	<1	nt	—	<1
**21**	I	—	3.69	2	nt	—	5.80
**22**	20.1	<1	3.03	4	6.31	2	11.5
**23**	I	—	I	—	I	—	nt
**24**	I	—	12.1	<1	nt	—	3.20
**25**	I	—	I	—	I	—	nt
**26**	8.95	<1	1.24	<1	nt	—	<1
**27**	I	—	3.14	<1	nt	—	<1

SI: Selectivity Index (CC_50_/IC_50_); I: Inactive; nt: Not tested.

### Antileishmanial activity

Antileishmanial activity of all of the compounds was tested *in vitro* against *L. donovani* and were also shown in Table 1. For the enone RALs, compounds **3**, **6**, **7**, **20**–**22**, **26**, and **27** displayed obvious antileishmanial activity with IC_50_ values ranging from 1.24 *μ*M to 9.11 *μ*M. Additionally, antiplasmodial compound **11** also showed antileishmanial activity with an IC_50_ value of 9.22 *μ*M.

### Antitrypanosomal activity

The bioassays against *T. cruzi* of all 27 molecules showed little activity against this parasite in initial screenings at 10 *μ*g/mL, except in the cases when the compound was also discovered to be toxic (data not shown). However, compounds **11** and **12** showed IC_50_ values of 11.9 and 17.2 *μ*M, respectively, with selectivity indexes of 28 and 88, making **12** a good candidate for *in vivo* studies.

### Toxicity

It should be emphasized that toxicity is a major concern of drug discovery and development. The above active compounds were evaluated for *in vitro* toxicity against a mammalian kidney cell line (Vero). The selectivity index (SI) was used as the evaluation parameter of drug potential of the test samples by comparing the toxicity on the Vero cell line (CC_50_) and the selective inhibitory effect on *P. falciparum*, *L. donovani* or *T. cruzi* (IC_50_) calculated here as CC_50_/IC_50_. Compounds **11**–**13** and **15** were found to be non-toxic and showed encouraging therapeutic indices, which were far greater than the value requested by the Medicine for Malaria Venture (SI > 10). Specially, antiplasmodial compounds **11** and **12** showed pronounced selectivity indices (SI > 180), and thus represent potentially promising antiplasmodial leads for further development. Compounds **12** and **13**, where **12**, with a 2-OH group, displayed 3-fold higher therapeutic ratio and equivalent or stronger activities than compound **13**, indicating that 2-OH is an important functionality for reducing the toxicity. For the enone RALs, most of them had high antileishmanial efficacy but poor therapeutic indices, indicating that the *cis* or *trans-*functionality has a positive contribution to toxicity, and that the antileishmanial activity of these compounds against *L. donovani* may well be related to their toxicity (Fig. 6).

### Cytotoxic activity

All of the compounds were also evaluated for their cytotoxic properties against six human cancer cell lines (lung carcinoma epithelian (A549), colon carcinoma (HCT-116), pancreatic carcinoma (BXPC-3), breast cancer (MCF-7), cervical epithelium (HeLa), and erythroleukemic (K562)) and a human umbilical vein endothelial cell (HUVEC) line (Table 2). Compounds **3**, **6**, **7**, **20**, **21**, **23**, **26**, and **27** with the enone functionality exhibited selectively cytotoxicity against the above cell lines with IC_50_ values less than 10 *μ*M. This further confirmed that the enone functionality was related to the toxicity.

**Table 2:** Cytotoxic activity of compounds **1**–**27**.

No.	Cytotoxicity IC_50_ (*μ*M)						
	HCT-116	BXPC-3	HeLa	MCF-7	A-549	K562	HUVEC
**1**	I	I	I	I	I	I	nt
**2**	I	I	I	I	I	I	nt
**3**	I	I	7.18	I	I	I	nt
**4**	I	I	I	I	I	I	nt
**5**	I	I	I	I	I	I	nt
**6**	I	8.47	I	I	I	I	8.59
**7**	1.09	5.07	I	I	I	I	I
**8**	I	I	I	I	I	I	I
**9**	I	I	I	I	I	I	nt
**10**	I	I	I	I	I	I	nt
**11**	I	I	I	I	I	I	I
**12**	I	I	I	I	I	I	I
**13**	I	I	I	I	I	I	I
**14**	I	I	I	I	I	I	nt
**15**	nt	nt	nt	nt	nt	nt	nt
**16**	I	I	I	I	I	I	nt
**17**	I	I	I	I	I	I	no
**18**	I	I	I	I	I	I	no
**19**	I	I	I	I	I	I	no
**20**	I	I	7.21	I	I	I	9.78
**21**	I	I	6.95	I	I	I	4.52
**22**	nt	nt	nt	nt	nt	nt	nt
**23**	3.51	nt	6.59	nt	9.75	nt	7.51
**24**	I	nt	I	nt	I	nt	I
**25**	I	I	I	I	I	I	I
**26**	9.16	7.22	4.02	I	I	I	4.81
**27**	2.83	nt	3.85	nt	7.30	nt	5.90
**Adriamycin**	0.21	0.033	0.60	0.86	0.16	0.25	—

I: Inactive; nt: Not tested.

## Conclusion

In summary, the natural products **1**–**6** and a group of derivatives **11**–**27** were semisynthesized with multi-gram scales of **7** and **8** by one or two steps with high yields. This semisynthetic route provided a convenient source of these RALs for further potentially biological applications. Compounds **11**–**13** showed strong antiplasmodial activity against *P. falciparum*, of which **11** and **12** were discovered to be promising non-toxic antiparasitic candidates. The structure-activity relationship analysis indicated that the acetoxy and acetonide groups are required for antiplasmodial activity, while the introduction of chlorine atom at C-5 is not necessary to the activity. Additionally, enone group can lead to an obvious toxicity and a comparatively lower therapeutic ratio, but 2-OH is critical for reducing toxicity. Further studies of selected compounds **11**–**13** on assessing their *in vivo* activities and a systematic optimization based upon these identified promising chemical leads are in progress and will be reported in due course.

## Methods

### General experimental procedures

NMR spectra were recorded with TMS as internal standard on an Agilent DD2 NMR spectrometer (500 and 125 MHz for ^1^H and ^13^C NMR, respectively). HRESIMS and ESIMS spectra were obtained from a Micromass Q-TOF spectrometer. Optical rotations were measured on a JASCO P-1020 digital polarimeter. Silica gel (Qing Dao Hai Yang Chemical Group Co.; 200–300 mesh), and Sephadex LH-20 (Amersham Biosciences) were used for column chromatography (CC). TLC silica gel plates (Yan Tai Zi Fu Chemical Group Co.; G60, F-254) were used for thin-layer chromatography. Semipreparative HPLC was carried out on a Waters 1525 system using a semipreparative C18 (Kromasil, 5 *μ*m, 10 × 250 mm) column equipped with a Waters 2996 photodiode array detector, at a flow rate of 2.0 mL/min.

### Fungal material

The fungal strain *C. lunatus* (CHNSCLM-0009) was isolated from the inner part of the zoanthid *Palythoa haddoni*, collected by hand from the South China Sea in April 2010. The fungus was identified as *C. lunatus* on the basis of 16 *S* rRNA gene analysis with the access code JF819163.

### Fermentation, extraction, and isolation

The fungal strain was cultivated in 10 L of optimal liquid medium (Medium I and Medium II, respectively) at 28 °C for 7 days on a rotary shaker at 120 rpm. Then the culture was filtered to separate the culture broth from the mycelia. The culture broth was extracted with an equal volume of EtOAc. The combined EtOAc solution was evaporated to dryness under a vacuum to give an EtOAc extract. The EtOAc extract (4.22 g) was subjected to Sephadex LH-20 column chromatography (120.0 cm × 3.0 cm i.d.) with 1.5 L petroleum ether/CH_2_Cl_2_/MeOH (2:1:1, v/v/v), and then recrystallization with MeOH/CH_2_Cl_2_ to obtain **8** (1.71 g) and **7** (1.35 g).

### Semisynthesis of cochliomycin C (2)

SO_2_Cl_2_ (0.045 mL, 0.36 mmol) in CH_2_Cl_2_ (5.0 mL) was added to the solution of zeaenol (**8**) (90.0 mg, 0.25 mmol) in dry CH_2_Cl_2_ (25.0 mL) at 0 °C. After 1 h the reaction was quenched by the addition of 25.0 mL of 5% aqueous NH_4_Cl solution and diluted with CH_2_Cl_2_ (30.0 mL). The organic layer was separated and the organic solvents were removed under a vacuum. The residue as purified by silica gel column chromatography (EtOAc/petroleum, 1:1, v/v) to give **16** as a white solid (87.5 mg, 90%). The resulting **16** (50.0 mg, 0.13 mmol) was then treated with 10% Pd-C (5.0 mg) in MeOH (35.0 mL) under H_2_ atmosphere, and the solution was stirred for 1.5 h at room temperature. The mixture was filtered and concentrated under a vacuum. The residue was purified by semipreparative HPLC to give **2** as a white solid (37.4 mg, 75%), the spectroscopic data of which were consistent with that reported in literature^17^.

### Allyl alcohol-oxidation of 8 to yield 3–6

Pyridine (24 *µ*L, 0.25 mmol) was added to a solution of **8** (30 mg, 0.082 mmol) in CH_2_Cl_2_ (20 mL). After the solution was cooled to 0 °C, the Dess-Martin periodinane (45 mg, 0.11 mmol) and NaHCO_3_ (14 mg, 0.16 mmol) were added as a solid. The mixture was stirred at 0 °C for 30 min, and then was allowed to stir at room temperature for 2 h. The mixture was diluted with Et_2_O (30 mL), 1 M aqueous Na_2_S_2_O_3_ (15 mL), and 5% aqueous NaHCO_3_ (15 mL) were added. This biphasic mixture was stirred for 0.5 h and the organic layer was separated and concentrated to dryness in vacuo. The crude mixture was immediately purified by Sephadex LH-20 CC eluted with petroleum ether/CH_2_Cl_2_/MeOH (2:1:1, v/v/v) to obtain pure compound **6** (19.5 mg). The obtained crude mixture was purified by silica gel CC eluted with EtOAc-petroleum (1:1), and then subjected to semipreparative HPLC to give the compounds **3** (6.8 mg), **4** (1.7 mg), **5** (4.3 mg), and **6** (5.2 mg), respectively. The structures of **3**–**6** were determined by comparing NMR data and MS spectrum with the literature^18^.

### Antiplasmodial assay

Activity against the causative agent of malaria was performed by culturing human erythrocytes and infecting them with *P. falciparum*, as described by Trager and Jensen^36^. Briefly, the W2 (Chloroquine resistant) and 3D7 (Chloroquine sensitive) strains of *P. falciparum* were cultured in RPMI 1640 medium (Sigma-Aldrich, USA) supplemented with 10% human serum (from O+ blood) at a hematocrit of 2% erythrocytes (O+) at 37 °C in a gas mixture of 5% CO_2_, 5% O_2_, and 90% N_2_. Parasites were synchronized by a temperature cycling technique as described by Almanza *et al*.^37^ Malaria bioassays were performed following the procedure of Corbett *et al*., which used Pico-Green to assess parasite growth inhibition by drugs and used chloroquine as positive control, with an IC_50_ of 32.9 nM^38^.

### Antileishmanial assay

The anti-leishmania activity was evaluated following the protocol described by Calderon *et al*.^39^, using the fluorescent DNA intercalator PicoGreen (Invitrogen, USA). The species responsible for visceral leishmaniasis, *L. donovani*, was used for the assays. Amphotericin B was used as the positive control and had an IC_50_ of 76.3 nM^40^.

### Antitrypanosomal assay

The antitrypanosomal test used the methods recommended in Romanha *et al*. for screening against this parasite with benznidazole as the control drug, with an IC_50_ of 3.85 *μ*M^41^. Briefly, the expression of the reporter gene for beta-galactosidase (β-Gal) in the Tulahuen clone C4 of *T. cruzi* is assessed by colorimetry at 570 nm, which correlates with the parasite growth^42^. Assays were performed on the intracellular amastigote form of the parasite infecting African green monkey kidney (Vero) cells, incubated for 120 h at 37 °C with 5% CO_2_.

### SRB and MTT assays

The cytotoxicity against HCT-116, BXPC-3, HeLa, MCF-7, A-549, and HUVEC cell lines was evaluated using the SRB method^43^. The cytotoxicity against K562 and Vero cell lines was evaluated using the MTT method^44^. Adriamycin was used as a positive control. All cells were cultivated in T-75 flasks containing 10% Fetal Bovine Serum (FBS), Dulbeco’s Modified Eagle Medium (DMEM) with 2 mM L-glutamine, and 1% penicillin-streptomycin at 37 °C in a humidified atmosphere with 5% CO_2_. For SRB assay, cells in their log phase of growth were seeded into 96 well micro plates (4 × 10^4^ cells per well) and followed by treating with test samples at 10 *μ*M or DMSO. After incubation at 37 °C for 72 h, the cells were fixed with the cold 20% (w/v) TCA for 2 h and stained with Sulforhodamine B (SRB) dye for 0.5 h. The protein-bound dye is dissolved in Tris base solution for OD determination at 540 nm on an ELISA Plate Reader. For MTT assay, cells (5 × 10^3^ cells per well) were plated in 96 well micro plates. After cell attachment overnight, new medium containing test compounds or DMSO was added. The plate was incubated for 48 h at 37 °C and incubated with 3-(4,5-dimethylthiazol-2-yl)-2,5-diphenyltetrazolium bromide (MTT) for additional 2–4 hours. The assay plate was then read at 490 nm on an ELISA Plate Reader. The data were obtained from experiments carried out in triplicate.

## Electronic supplementary material

supporting information
