# Supplementary material for: Discovery, Semisynthesis, Antiparasitic and Cytotoxic Evaluation of 14-Membered Resorcylic Acid Lactones and Their Derivatives
Source: Sci Rep. 2017 Sep 18;7:11822. doi: 10.1038/s41598-017-12336-0 (PMC5603512; doi:10.1038/s41598-017-12336-0)
Supplement: Supplementary file 1 — supporting information [file 41598_2017_12336_MOESM1_ESM.pdf]

Discovery, Semisynthesis, Antiparasitic and Cytotoxic Evaluation of 14-Membered Resorcylic Acid Lactones and Their Derivatives

Xue-Qing Zhang,[†,‡] Carmenza Spadafora,[§] Laura M. Pineda,[§] Michelle G. Ng,[§] Ji-Hong Sun,[†,‡] Wei Wang,[†,‡] Chang-Yun Wang,[†,‡] Yu-Cheng Gu,[Δ] and Chang-Lun Shao[*†,‡]

[†] Key Laboratory of Marine Drugs, The Ministry of Education of China, School of Medicine and Pharmacy, Ocean University of China, Qingdao 266003, The People's Republic of China

[‡] Laboratory for Marine Drugs and Bioproducts, Qingdao National Laboratory for Marine Science and Technology, Qingdao 266200, The People's Republic of China

[§] Center of Cellular and Molecular Biology of Diseases, Instituto de Investigaciones Científicas y Servicios de Alta Tecnología, City of Knowledge, Clayton, Apartado 0816-02852, Panama

[Δ] Syngenta Jealott's Hill International Research Centre, Bracknell, Berkshire, RG42 6EY, United Kingdom

Correspondence and requests for materials should be addressed to C.L.S. (shaochanglun@163.com)

List of Supporting Information

I. General Experimental procedures

Figure S1. ^{1}H NMR (500 MHz, $CDCl_3$) spectrum of compound **1**.

Figure S2. ^{13}C NMR (125 MHz, $CDCl_3$) spectrum of compound **1**.

Figure S3. ESIMS spectrum of compound **1**.

Figure S4. ^{1}H NMR (500 MHz, $CDCl_3$) spectrum of compound **2**.

Figure S5. HRESIMS spectrum of compound **2**.

Figure S6. ^{1}H NMR (500 MHz, DMSO-d_6) spectrum of compound **3**.

Figure S7. ^{13}C NMR (125 MHz, DMSO-d_6) spectrum of compound **3**.

Figure S8. ESIMS spectrum of compound **3**.

Figure S9. ^{1}H NMR (500 MHz, DMSO-d_6) spectrum of compound **4**.

Figure S10. ^{13}C NMR (125 MHz, DMSO-d_6) spectrum of compound **4**.

Figure S11. ESIMS spectrum of compound **4**.

Figure S12. ^{1}H NMR (500 MHz, DMSO-d_6) spectrum of compound **5**.

Figure S13. ^{13}C NMR (125 MHz, DMSO-d_6) spectrum of compound **5**.

Figure S14. ESIMS spectrum of compound **5**.

Figure S15. ^{1}H NMR (500 MHz, DMSO-d_6) spectrum of compound **6**.

Figure S16. ^{13}C NMR (125 MHz, DMSO-d_6) spectrum of compound **6**.

Figure S17. ESIMS spectrum of compound **6**.

Figure S18. ^{1}H NMR (500 MHz, $CDCl_3$) spectrum of compound **7**.

Figure S19. ESIMS spectrum of compound **7**.

Figure S20. ^{1}H NMR (500 MHz, $CDCl_3$) spectrum of compound **8**.

Figure S21. ESIMS spectrum of compound **8**.

Figure S22. ^{1}H NMR (500 MHz, $CDCl_3$) spectrum of compound **9**.

Figure S23. ESIMS spectrum of compound **9**.

Figure S24. ^{1}H NMR (500 MHz, $CDCl_3$) spectrum of compound **10**.

Figure S25. ESIMS spectrum of compound **10**.

Figure S26. ^{1}H NMR (500 MHz, $CDCl_3$) spectrum of compound **11**.

Figure S27. ^{13}C NMR (125 MHz, $CDCl_3$) spectrum of compound **11**.

Figure S28. ESIMS spectrum of compound **11**.

Figure S29. ^{1}H NMR (500 MHz, $CDCl_3$) spectrum of compound **12**.

Figure S30. ^{13}C NMR (125 MHz, $CDCl_3$) spectrum of compound **12**.

Figure S31. HRESIMS spectrum of compound **12**.

Figure S32. ^{1}H NMR (500 MHz, $CDCl_3$) spectrum of compound **13**.

Figure S33. ^{13}C NMR (125 MHz, $CDCl_3$) spectrum of compound **13**.

Figure S34. ESIMS spectrum of compound **13**.

Figure S35. ^{1}H NMR (500 MHz, $CDCl_3$) spectrum of compound **14**.

Figure S36. ^{13}C NMR (125 MHz, $CDCl_3$) spectrum of compound **14**.

Figure S37. HRESIMS spectrum of compound **14**.

Figure S38. ^{1}H NMR (500 MHz, $CDCl_3$) spectrum of compound **15**.

Figure S39. ^{13}C NMR (125 MHz, $CDCl_3$) spectrum of compound **15**.

Figure S40. HRESIMS spectrum of compound **15**.

Figure S41. ^{1}H NMR (500 MHz, $CDCl_3$) spectrum of compound **16**.

Figure S42. ^{13}C NMR (125 MHz, $CDCl_3$) spectrum of compound **16**.

Figure S43. ESIMS spectrum of compound **16**.

Figure S44. ^{1}H NMR (500 MHz, $CDCl_3$) spectrum of compound **17**.

Figure S45. ^{13}C NMR (125 MHz, $CDCl_3$) spectrum of compound **17**.

Figure S46. HRESIMS spectrum of compound **17**.

Figure S47. ^{1}H NMR (500 MHz, $CDCl_3$) spectrum of compound **18**.

Figure S48. ^{13}C NMR (125 MHz, $CDCl_3$) spectrum of compound **18**.

Figure S49. HRESIMS spectrum of compound **18**.

Figure S50. ^{1}H NMR (500 MHz, $CDCl_3$) spectrum of compound **19**.

Figure S51. ^{13}C NMR (125 MHz, $CDCl_3$) spectrum of compound **19**.

Figure S52. HRESIMS spectrum of compound **19**.

Figure S53. ^{1}H NMR (500 MHz, Acetone-d_6) spectrum of compound **20**.

Figure S54. ^{13}C NMR (125 MHz, Acetone-d_6) spectrum of compound **20**.

Figure S55. ESIMS spectrum of compound **20**.

Figure S56. ^{1}H NMR (500 MHz, $CDCl_3$) spectrum of compound **21**.

Figure S57. ^{13}C NMR (125 MHz, $CDCl_3$) spectrum of compound **21**.

Figure S58. HRESIMS spectrum of compound **21**.

Figure S59. ^{1}H NMR (500 MHz, $CDCl_3$) spectrum of compound **22**.

Figure S60. ^{13}C NMR (125 MHz, $CDCl_3$) spectrum of compound **22**.

Figure S61. HRESIMS spectrum of compound **22**.

Figure S62. ^{1}H NMR (500 MHz, Acetone-d_6) spectrum of compound **23**.

Figure S63. ^{13}C NMR (125 MHz, Acetone-d_6) spectrum of compound **23**.

Figure S64. ESIMS spectrum of compound **23**.

Figure S65. ^{1}H NMR (500 MHz, $CDCl_3$) spectrum of compound **24**.

Figure S66. ^{13}C NMR (125 MHz, $CDCl_3$) spectrum of compound **24**.

Figure S67. HRESIMS spectrum of compound **24**.

Figure S68. ^{1}H NMR (500 MHz, Acetone-d_6) spectrum of compound **25**.

Figure S69. ^{13}C NMR (125 MHz, Acetone-d_6) spectrum of compound **25**.

Figure S70. ESIMS spectrum of compound **25**.

Figure S71. ^{1}H NMR (500 MHz, $CDCl_3$) spectrum of compound **26**.

Figure S72. ESIMS spectrum of compound **26**.

Figure S73. ^{1}H NMR (500 MHz, $CDCl_3$) spectrum of compound **27**.

Figure S74. ^{13}C NMR (125 MHz, $CDCl_3$) spectrum of compound **27**.

Figure S75. HRESIMS spectrum of compound **27**.

References

I. General Experimental procedures

Acetonide of Compounds 2–3, 5–8, and 16. A mixture of **2** (48.0 mg), 2, 2-dimethoxypropane (4.5 mL), *p*-TsOH (10.0 mg) and acetone (10 mL) was stirred at room temperature for 1 h. Saturated aqueous $NaHCO_3$ (15 mL) was then added, and the reaction mixture was extracted with EtOAc (20 mL × 3). The organic solvents were removed with a high-vacuum pump, and the crude mixture was subjected to Sephadex LH-20 column chromatography with 1 L petroleum ether/CH_2Cl_2/MeOH (2:1:1, v/v/v) to obtain compound **18** (50.2 mg). Acetonide of compounds **3**, **5–8**, and **16** under the same reaction conditions gave the corresponding products **23**, **25**, **20**, **26**, **1**, and **17**, respectively. The known structures of **1**, **20**, **23**, **25**, and **26** were determined by comparing NMR data and MS spectrum with the literature.[1]

Compound 17: white, amorphous powder; $[\alpha]^{25}_{D}$ +41.0 (*c* 0.13, MeOH); ^{1}H NMR ($CDCl_3$, 500 MHz) δ 11.36 (1H, s, 2-OH), 6.64 (1H, dd, J = 16.1, 1.4 Hz, H-1′), 6.46 (1H, s, H-3), 5.99 (1H, ddd, J = 15.6, 8.2, 5.0 Hz, H-8′), 5.51 (1H, ddd, J = 16.1, 9.0, 3.7 Hz, H-2′), 5.44 (1H, ddt, J = 15.6, 8.6, 1.3 Hz, H-7′), 5.39 (1H, m, H-10′), 4.55 (1H, t, J = 8.6 Hz, H-6′), 4.19 (1H, m, H-4′), 3.91 (3H, s, 4-OCH_3), 3.80 (1H, dd, J = 8.6, 2.5 Hz, H-5′), 2.87 (1H, m, Ha-3′), 2.56 (1H, m, Ha-9′), 2.43(1H, m, Hb-9′), 2.36 (1H, m, Hb-3′), 1.43 (3H, s), 1.39 (6H, overlapped, -CH_3×2); ^{13}C NMR ($CDCl_3$, 125 MHz) δ 170.7 (C), 162.4 (C), 160.0 (C), 140.0 (C), 130.8 (CH), 130.4 (CH), 129.2 (CH), 128.9 (CH), 114.2 (C), 108.7 (C), 106.5 (C), 99.6 (CH), 81.0 (CH), 75.6 (CH), 71.4 (CH), 68.5 (CH), 56.5 (CH_3), 37.2 (CH_2), 37.2 (CH_2), 27.3 (CH_3), 27.1 (CH_3), 19.4 (CH_3); HRESIMS *m/z* 461.1338 $[M + Na]^+$ (calcd for $C_{22}H_{27}O_7ClNa$, 461.1338); 95% yield.

Compound 18: white, amorphous powder; $[\alpha]^{25}_{D}$ −23.2 (*c* 0.05, MeOH); ^{1}H NMR ($CDCl_3$, 500 MHz) δ 11.72 (1H, s, 2-OH), 6.68 (1H, dd, J = 16.0, 2.1 Hz, H-1′), 6.48 (1H, s, H-3), 5.61 (1H, ddd, J = 16.0, 10, 2.5 Hz, H-2′), 5.24 (1H, m, H-10′), 5.30 (1H, m), 4.17 (2H, m), 3.92 (3H, s, 4-OCH_3), 3.79 (1H, dd, J = 7.9, 1.8 Hz, H-5′), 2.94 (1H, m), 2.37 (1H, m), 2.01 (1H, m), 1.83 (1H, m), 1.62 (4H, overlapped), 1.42 (3H, s), 1.37 (3H, d, J = 6.4 Hz, H-11′), 1.36 (3H, s); ^{13}C NMR ($CDCl_3$, 125 MHz) δ 170.8 (C), 162.9 (C), 160.0 (C), 139.8 (C), 132.1 (CH), 128.9 (CH), 114.6 (C), 107.7 (C), 106.1 (C), 99.7 (CH), 80.6 (CH), 73.9 (CH), 73.8 (CH), 69.4 (CH), 56.6 (CH_3), 35.7 (CH_2), 34.7 (CH_2), 33.3 (CH_2), 29.8 (CH_2), 27.4 (CH_3), 27.3 (CH_3), 20.1 (CH_3); HRESIMS *m/z* 463.1495 $[M + Na]^+$ (calcd for $C_{22}H_{29}O_7ClNa$, 463.1494); 95% yield.

Acylation of compounds 1–3 and 6–8. To a solution of compound **1** (100 mg, 0.25 mmol) in

acetone (15 mL) was added excess anhydrous K_2CO_3 and 5.0 mL of acetic anhydride, and the mixture was stirred at 40 ℃ for 12 h. When the reaction was completed, the solvent was removed in vacuo. Water (15 mL) was then added, and the reaction mixture was extracted with EtOAc (20 mL × 3). The organic solvents were removed under a vacuum, and the crude mixture was subjected to Sephadex LH-20 CC eluted with petroleum ether/CH_2Cl_2/MeOH (2:1:1, v/v/v) to obtain the compound **11** (98.8 mg). Acylation of compounds **2**, **3**, and **6**–**8** under the same reaction conditions gave the corresponding products **19**, **24**, **21**, **22**, **27**, and **12**–**15**, respectively. The known structures of **11** and **13** were determined by comparing NMR data and MS spectrum with the literature.[2]

Compound 12: white, amorphous powder; $[\alpha]^{25}_{D}$ –56.8 (*c* 0.12, MeOH); ^{1}H NMR ($CDCl_3$, 500 MHz) δ 12.04 (1H, s, 2-OH), 7.07 (1H, d, *J* = 15.0 Hz, H-1′), 6.40 (2H, overlapped, H-3, H-5), 6.14 (1H, m), 5.82 (1H, m), 5.69 (1H, m), 5.49 (1H, t, *J* = 8.2 Hz), 5.39 (2H, overlapped), 5.01 (1H, m, H-10′), 3.81 (3H, s, 4-OCH_3), 2.66 (1H, m), 2.47 (3H, overlapped), 2.05 (9H, -CH_3×3), 1.47 (3H, d, *J* = 6.3 Hz, H-11′); ^{13}C NMR ($CDCl_3$, 125 MHz) δ 171.4 (C), 170.6 (C), 170.3 (C), 170.2 (C), 165.9 (C), 164.2 (C), 142.5 (C), 134.5 (CH), 132.9 (CH), 127.5 (CH), 126.0 (CH), 108.0 (C), 103.7 (CH), 100.2 (CH), 73.9 (CH), 73.8 (CH), 73.8 (CH), 71.1 (CH), 55.6 (CH_3), 37.2 (CH_2), 32.3 (CH_2), 21.2 (CH_3), 21.1 (CH_3), 21.0 (CH_3), 19.3 (CH_3); HRESIMS *m/z* 491.1921 $[M + H]^+$ (calcd for $C_{25}H_{31}O_{10}$, 491.1912); 67% yield.

Compound 14: white, amorphous powder; $[\alpha]^{25}_{D}$ +41.9 (*c* 0.14, MeOH); ^{1}H NMR ($CDCl_3$, 500 MHz) δ 6.82 (1H, d, *J* = 15.0 Hz, H-1′), 6.72 (1H, d, *J* = 2.5 Hz, H-5), 6.54 (1H, d, *J* = 2.5 Hz, H-3), 6.02 (1H, m, H-8′), 5.94 (1H, ddd, *J* = 15.0, 9.0, 4.7 Hz, H-2′), 5.62 (1H, dd, *J* = 15.5, 7.9 Hz, H-7′), 5.46 (1H, m, H-10′), 5.31 (1H, m), 5.12 (2H, overlapped), 3.80 (3H, s, 4-OCH_3), 2.95 (2H, overlapped), 2.55 (2H, overlapped), 2.58–2.28 (8H, -CH_2×4), 1.37 (3H, d, *J* = 6.1, H-11′), 1.23 (3H, t, *J* = 7.5 Hz, -CH_3), 1.10 (9H, -CH_3×3); ^{13}C NMR ($CDCl_3$, 125 MHz) δ 173.8 (C), 173.5 (C), 173.4 (C), 172.7 (C), 165.6 (C), 161.3 (C), 150.8 (C), 139.1 (C), 133.8 (CH), 131.5 (CH), 126.6 (CH), 126.9 (CH), 117.7 (C), 110.5 (CH), 107.7 (CH), 73.6 (CH), 73.6 (CH), 72.9 (CH), 72.3 (CH), 55.7 (CH_3), 37.8 (CH_2), 37.8 (CH_2), 27.8 (CH_2), 27.7 (CH_2), 27.6 (CH_2), 27.6 (CH_2), 20.1 (CH_3), 9.3 (CH_3), 9.2 (CH_3), 9.0 (CH_3), 9.0 (CH_3); HRESIMS *m/z* 611.2473 $[M + Na]^+$ (calcd for $C_{31}H_{40}O_{11}Na$, 611.2463); 92% yield.

Compound 15: white, amorphous powder; $[\alpha]^{25}_{D}$ −45.4 (*c* 0.05, MeOH); ^{1}H NMR ($CDCl_3$, 500

MHz) δ 7.66 (1H, d, J = 0.6 Hz), 7.56 (1H, s), 7.49 (2H, d, J = 9.1 Hz), 7.37 (1H, d, J = 3.2 Hz), 7.19 (1H, m), 7.06 (2H, m), 6.97 (1H, m), 6.81 (1H, brs), 6.71 (1H, d, J = 1.8 Hz), 6.58 (1H, m), 6.48 (1H, m), 6.41 (2H, overlapped), 6.20–6.05 (2H, overlapped), 5.80 (2H, overlapped), 5.69 (1H, m), 5.52 (1H, m), 5.23 (1H, m), 3.83 (3H, s, 4-OCH_3), 2.88–2.76 (2H, overlapped), 2.56–2.38 (2H, overlapped), 1.29 (3H, d, J = 6.2 Hz, H-11′); ^{13}C NMR ($CDCl_3$, 125 MHz) δ 165.4 (C), 161.4 (C), 157.9 (C), 157.5 (C), 157.5 (C), 156.5 (C), 149.9 (C), 147.4, 146.9, 146.8, 146.8, 144.2, 144.1, 143.9, 143.9, 139.2, 134.6, 131.6, 129.2, 126.4, 119.9, 118.9, 118.9, 118.7, 118.0, 112.4, 112.1, 112.0, 112.0, 110.9, 107.9, 77.4 (CH), 74.0 (CH), 73.4 (CH), 71.9 (CH), 55.8 (CH_3), 38.0 (CH_2), 33.6 (CH_2), 20.0 (CH_3). HRESIMS m/z 763.1634 $[M + Na]^+$ (calcd for $C_{39}H_{32}O_{15}Na$, 763.1633); 85% yield.

Compound 19: white, amorphous powder; $[\alpha]^{25}_{D}$ +15.1 (c 0.05, MeOH); ^{1}H NMR ($CDCl_3$, 500 MHz) δ 6.75 (1H, d, J = 15.9 Hz, H-1′), 6.67 (1H, s, H-3), 5.94 (1H, m, H-2′), 5.27 (1H, m), 5.20 (1H, m), 4.98 (2H, overlapped), 3.90 (3H, s, 4-OCH_3), 2.87 (1H, m), 2.47 (1H, m), 2.26 (3H, s, -CH_3), 2.06 (3H, s, -CH_3), 2.01 (6H, overlapped, -CH_3 ×2), 1.70–1.35 (6H, overlapped), 1.32 (3H, d, J = 6.0 Hz, H-11′); ^{13}C NMR ($CDCl_3$, 125 MHz) δ 170.4 (C), 170.2 (C), 170.2 (C), 168.7 (C), 165.6 (C), 156.2 (C), 147.1 (C), 136.1, 132.9, 128.7, 120.4, 119.8, 105.7, 74.6 (CH), 71.9 (CH), 71.8 (CH), 70.0 (CH), 56.7 (CH_3), 33.9 (CH_2), 33.9 (CH_2), 28.9 (CH_2), 28.9 (CH_2), 21.1 (CH_3), 21.1 (CH_3), 21.0 (CH_3), 21.0 (CH_3), 20.9 (CH_3); HRESIMS m/z 569.1788 $[M + H]^+$ (calcd for $C_{27}H_{34}O_{11}Cl$, 569.1784); 87% yield.

Compound 21: white, amorphous powder; $[\alpha]^{25}_{D}$ –8.5 (c 0.08, MeOH); ^{1}H NMR ($CDCl_3$, 500 MHz) δ 7.03 (1H, m), 6.80 (1H, d, J = 1.8 Hz, H-5), 6.62 (1H, d, J = 15.9 Hz, H-1′), 6.58 (1H, d, J = 1.8 Hz, H-3), 6.22 (1H, d, J = 15.9 Hz, H-7′), 5.99 (1H, m), 5.75 (1H, d, J = 2.4 Hz, H-5′), 5.49 (1H, m), 5.29 (1H, m), 3.81 (3H, s, 4-OCH_3), 2.62 (2H, m), 2.43 (2H, m), 2.26 (3H, s, -CH_3), 2.12 (3H, s, -CH_3), 2.09 (3H, s, -CH_3), 1.42 (3H, d, J = 6.1 Hz, H-11′); ^{13}C NMR ($CDCl_3$, 125 MHz) δ 193.3 (C), 170.3 (C), 170.2 (C), 168.8 (C), 165.7 (C), 161.2 (C), 149.5 (C), 143.9 (CH), 143.9 (C), 138.3 (CH), 131.3 (CH), 128.4 (CH), 118.3 (C), 109.6 (CH), 107.7 (CH), 75.7 (CH), 72.9 (CH), 69.9 (CH), 55.8 (CH_3), 39.0 (CH_2), 32.8 (CH_2), 21.1 (CH_3), 21.0 (CH_3), 20.8 (CH_3), 20.7 (CH_3); HRESIMS m/z 511.1577 $[M + Na]^+$ (calcd for $C_{25}H_{28}O_{10}Na$, 511.1575); 87% yield.

Compound 22: white, amorphous powder; $[\alpha]^{25}_{D}$ +67.1 (c 0.21, MeOH); ^{1}H NMR ($CDCl_3$, 500 MHz) δ 7.96 (1H, dd, J = 4.0, 1.2 Hz), 7.75 (1H, dd, J = 4.0, 1.2 Hz), 7.67 (1H, dd, J = 4.0, 1.2

Hz), 7.55 (3H, overlapped), 7.17 (1H, m), 7.07–6.99 (4H, overlapped), 6.73 (1H, d, *J* = 2.2 Hz), 6.68 (1H, d, *J* = 15.8 Hz), 6.42 (1H, d, *J* = 15.8 Hz), 6.33 (1H, m), 6.05 (1H, d, *J* = 4.0 Hz), 5.91 (1H, m), 5.32 (1H, m), 3.83 (3H, s, 4-OCH_3), 2.82–2.68 (2H, overlapped), 2.46 (1H, m), 2.32 (1H, m), 1.06 (3H, d, *J* = 6.3 Hz, H-11′); ^{13}C NMR ($CDCl_3$, 125 MHz) δ 195.1(C), 165.8 (C), 161.1 (C), 161.0 (C), 160.9 (C), 160.1 (C), 149.0 (C), 144.2, 138.7, 135.2, 134.6, 134.2, 134.1, 133.9, 133.2, 133.1, 132.6, 132.4, 130.4, 129.6, 129.2, 128.3, 128.2, 128.0, 119.1, 109.6 (CH), 107.7 (CH), 76.6 (CH), 73.3 (CH), 69.4 (CH), 55.9 (CH_3), 39.8 (CH_2), 34.1 (CH_2), 20.5 (CH_3). HRESIMS *m/z* 715.0738 $[M + Na]^+$ (calcd for $C_{34}H_{28}O_{10}S_3Na$, 715.0737); 85% yield.

Compound 24: white, amorphous powder; $[\alpha]^{25}_{D}$ –49.1 (*c* 0.08, MeOH); ^{1}H NMR ($CDCl_3$, 500 MHz) δ 7.04 (1H, m), 6.83 (2H, overlapped), 6.58 (1H, s, H-3), 6.26 (1H, d, *J* = 16.3 Hz), 6.06 (1H, m), 5.45 (2H, overlapped), 5.34 (1H, m, H-10′), 3.83 (3H, s, 4-OCH_3), 2.75 (1H, m), 2.65 (1H, m), 2.41 (2H, m), 2.29 (3H, s, -CH_3), 2.11 (6H, overlapped, -$CH_3 \times 2$), 1.44 (3H, d, *J* = 6.2 Hz, H-11′); ^{13}C NMR ($CDCl_3$, 125 MHz) δ 194.0 (C), 170.3 (C), 170.0 (C), 169.4 (C), 164.9 (C), 161.7 (C), 151.2 (C), 144.6 (CH), 139.5 (C), 133.5 (CH), 131.7 (CH), 126.8 (CH), 117.0 (C), 111.2 (CH), 108.2 (CH), 75.0 (CH), 72.0 (CH), 70.6 (CH), 55.8 (CH_3), 37.7 (CH_2), 33.3 (CH_2), 21.1 (CH_3), 21.1 (CH_3), 20.6 (CH_3), 19.7 (CH_3); HRESIMS *m/z* 511.1575 $[M + Na]^+$ (calcd for $C_{25}H_{28}O_{10}Na$, 511.1575); 87% yield.

Compound 27: white, amorphous powder; $[\alpha]^{25}_{D}$ –55.7 (*c* 0.13, MeOH); ^{1}H NMR ($CDCl_3$, 500 MHz) δ 12.16 (1H, s, 2-OH), 6.95 (1H, d, *J* = 15.0 Hz, H-1′), 6.49 (1H, dd, *J* = 11.5, 2.6 Hz, H-7′), 6.37 (2H, overlapped, H-3, H-5), 6.23 (1H, td, *J* = 11.5, 2.6 Hz, H-8′), 5.88 (1H, ddd, *J* = 15.0, 10.3, 4.3 Hz, H-2′), 5.58 (1H, brs), 5.24 (2H, overlapped), 3.80 (3H, s, 4-OCH_3), 3.55 (1H, m), 2.42 (3H, overlapped), 2.42 (3H, s, -CH_3), 2.10 (3H, s, -CH_3), 1.35 (3H, d, *J* = 6.0, H-11′); ^{13}C NMR ($CDCl_3$, 125 MHz) δ 192.9 (C), 171.5 (C), 170.8 (C), 170.3 (C), 166.1 (C), 164.3 (C), 147.0 (CH), 143.0 (C), 133.9 (CH), 129.4 (CH), 126.0 (CH), 108.5 (C), 103.6 (CH), 100.2 (CH), 80.1 (CH), 74.0 (CH), 72.9 (CH), 55.6 (CH_3), 36.9 (CH_2), 33.3 (CH_2), 21.2 (CH_3), 20.9 (CH_3), 20.8 (CH_3); HRESIMS *m/z* 447.1652 $[M + H]^+$ (calcd for $C_{23}H_{27}O_9$, 447.1650); 65% yield.

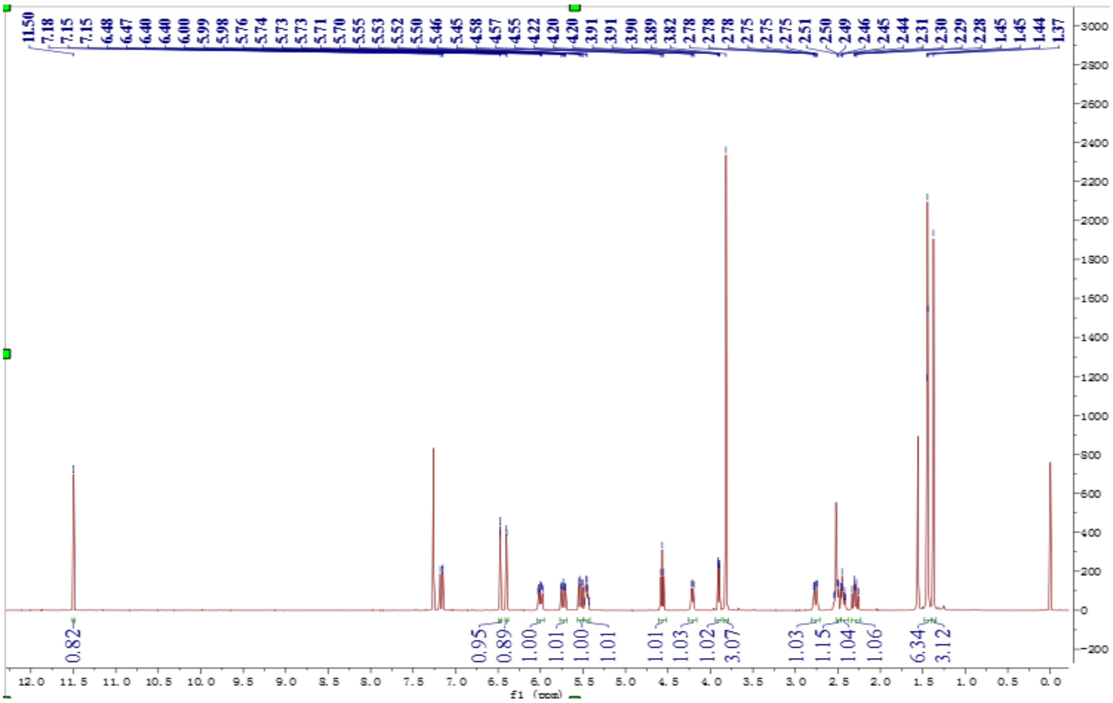

Figure S1. ^{1}H NMR (500 MHz, $CDCl_3$) spectrum of compound **1**.

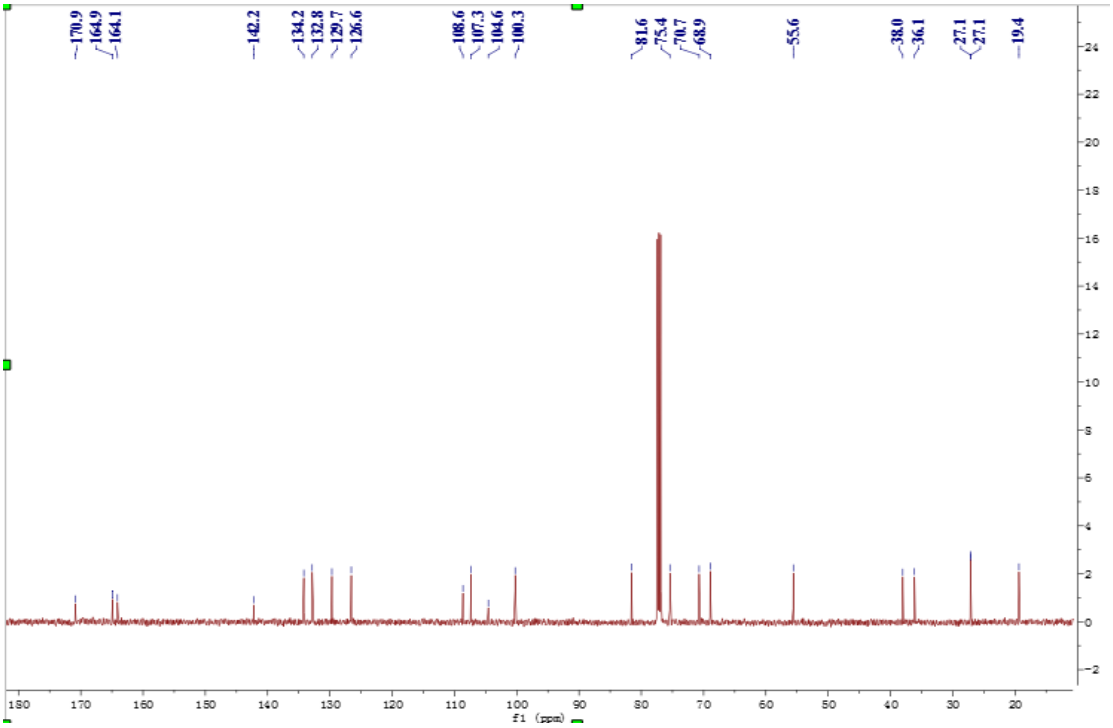

Figure S2. ^{13}C NMR (125 MHz, $CDCl_3$) spectrum of compound **1**.

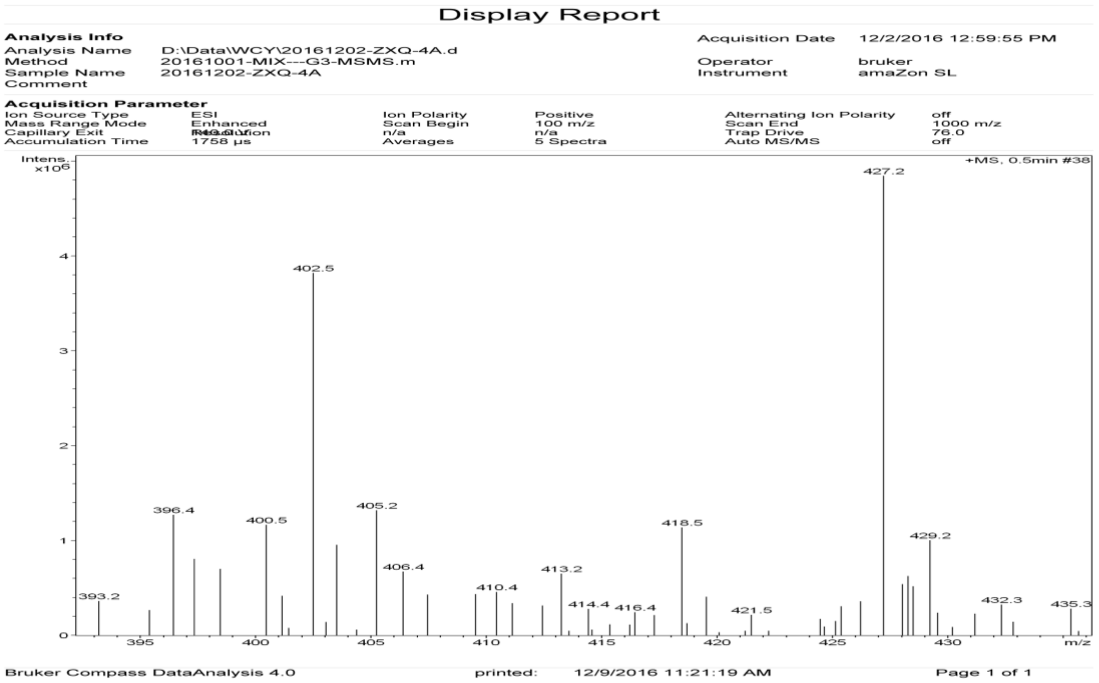

Figure S3. ESIMS spectrum of compound **1**.

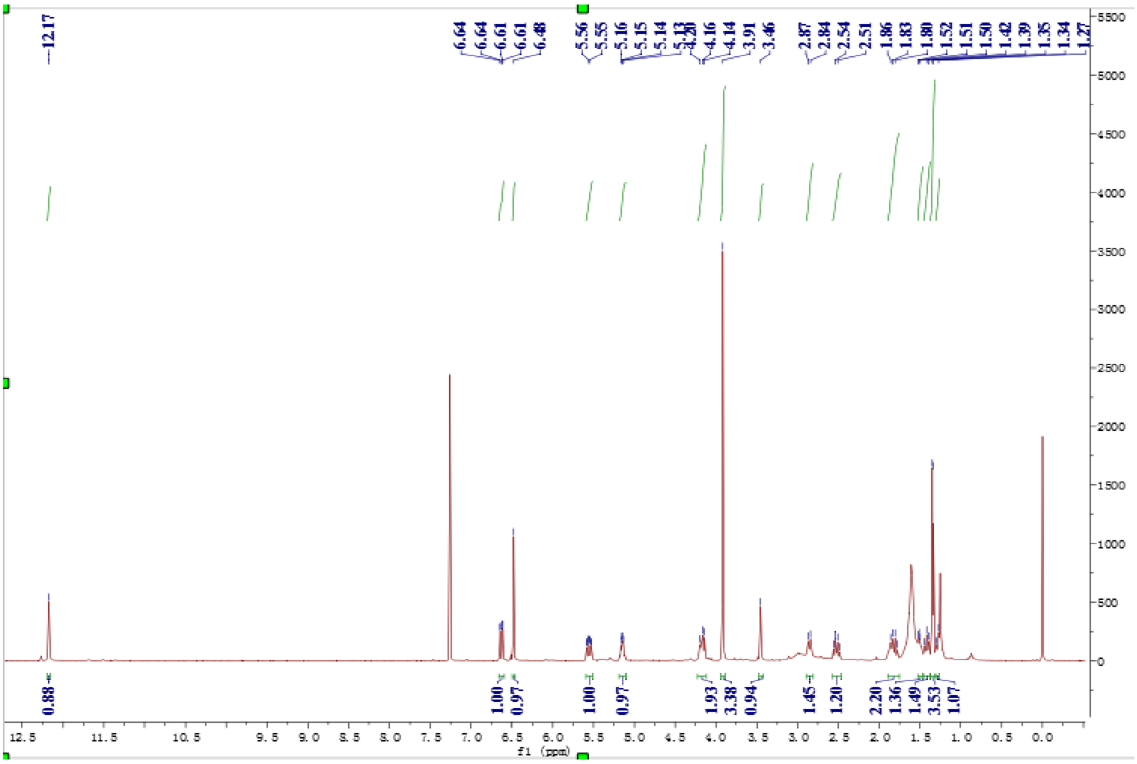

Figure S4. ^{1}H NMR (500 MHz, $CDCl_3$) spectrum of compound **2**.

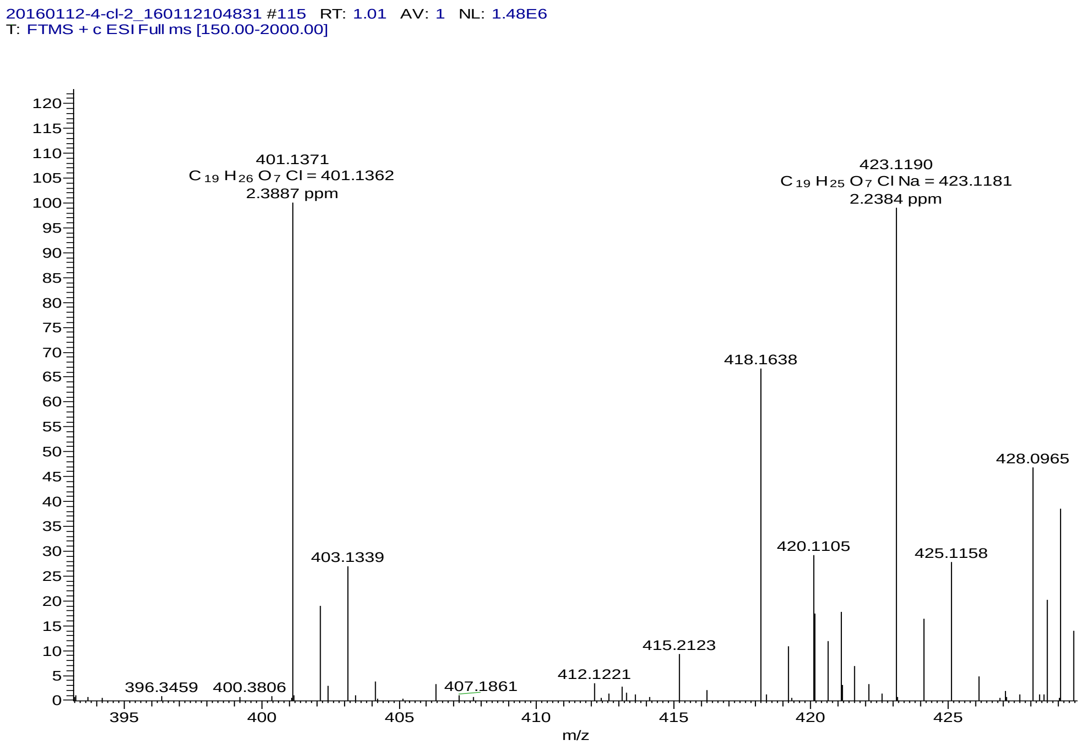

Figure S5. HRESIMS spectrum of compound **2**.

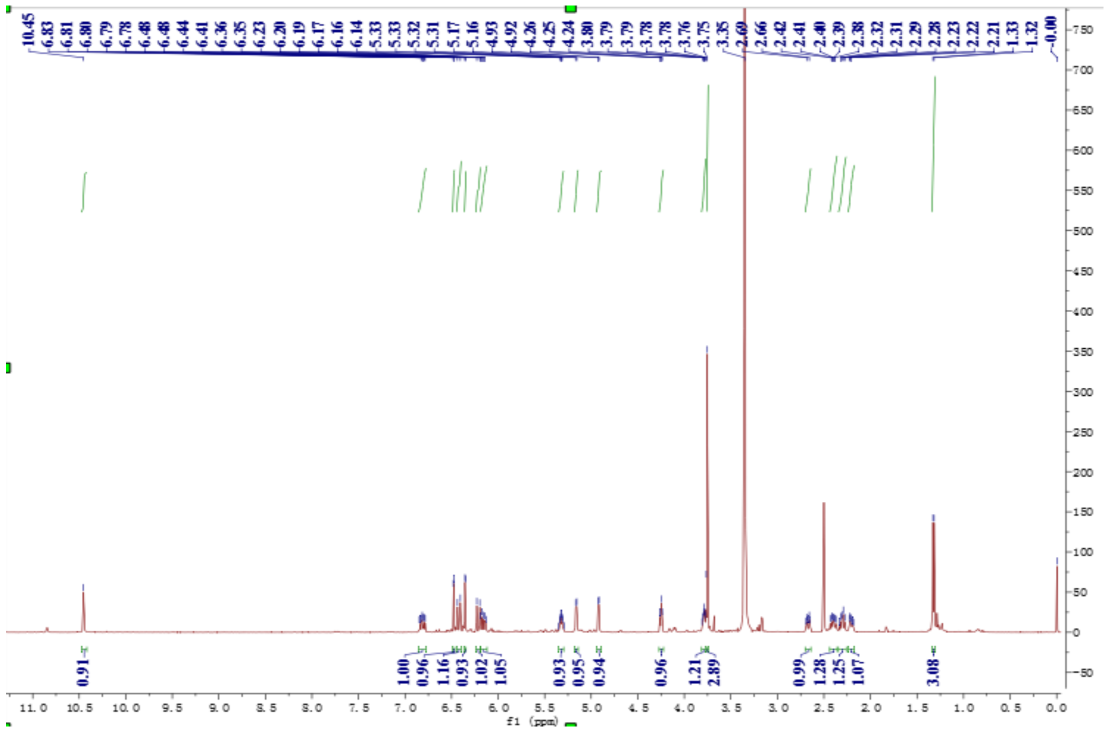

Figure S6. ^{1}H NMR (500 MHz, DMSO-d_6) spectrum of compound **3**.

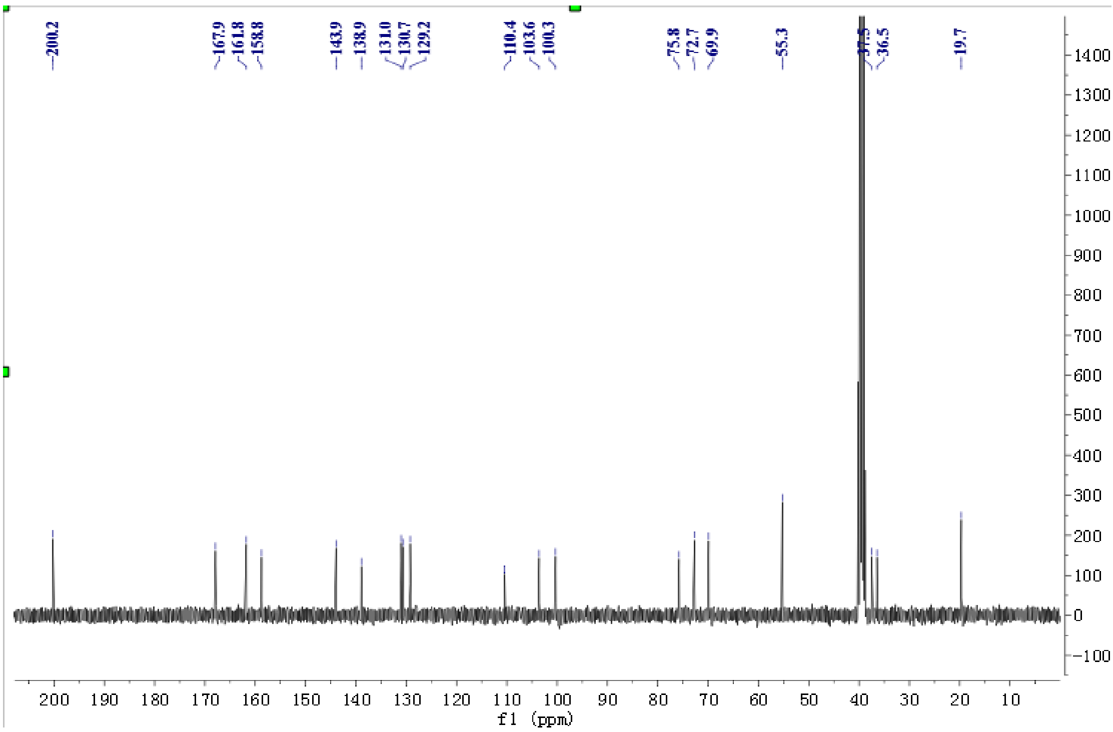

Figure S7. ^{13}C NMR (125 MHz, DMSO-d_6) spectrum of compound **3.**

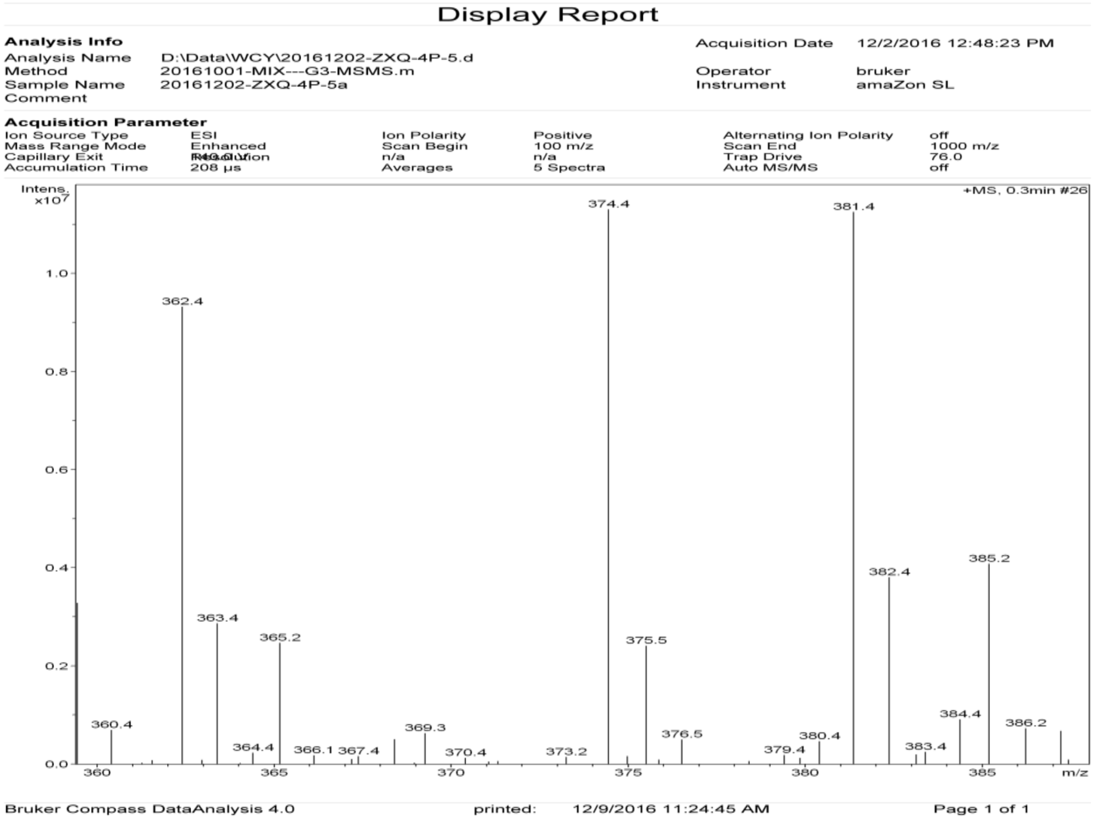

Figure S8. ESIMS spectrum of compound **3**.

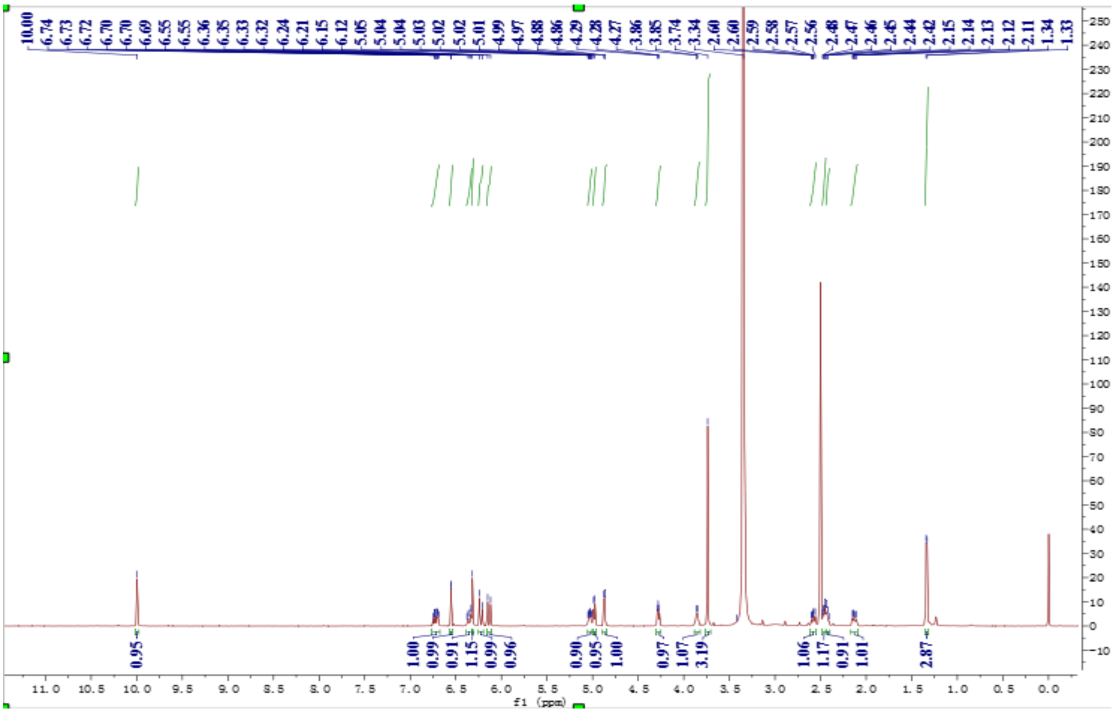

Figure S9. ^{1}H NMR (500 MHz, DMSO-d_6) spectrum of compound **4**.

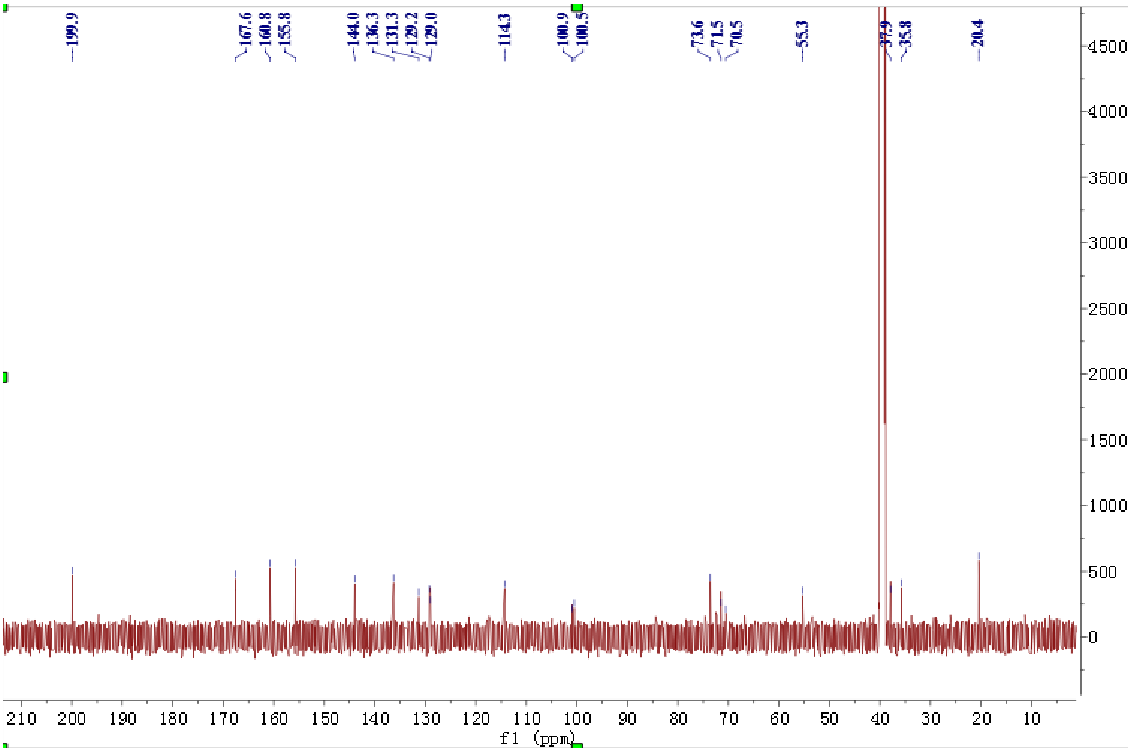

Figure S10. ^{13}C NMR (125 MHz, DMSO-d_6) spectrum of compound **4.**

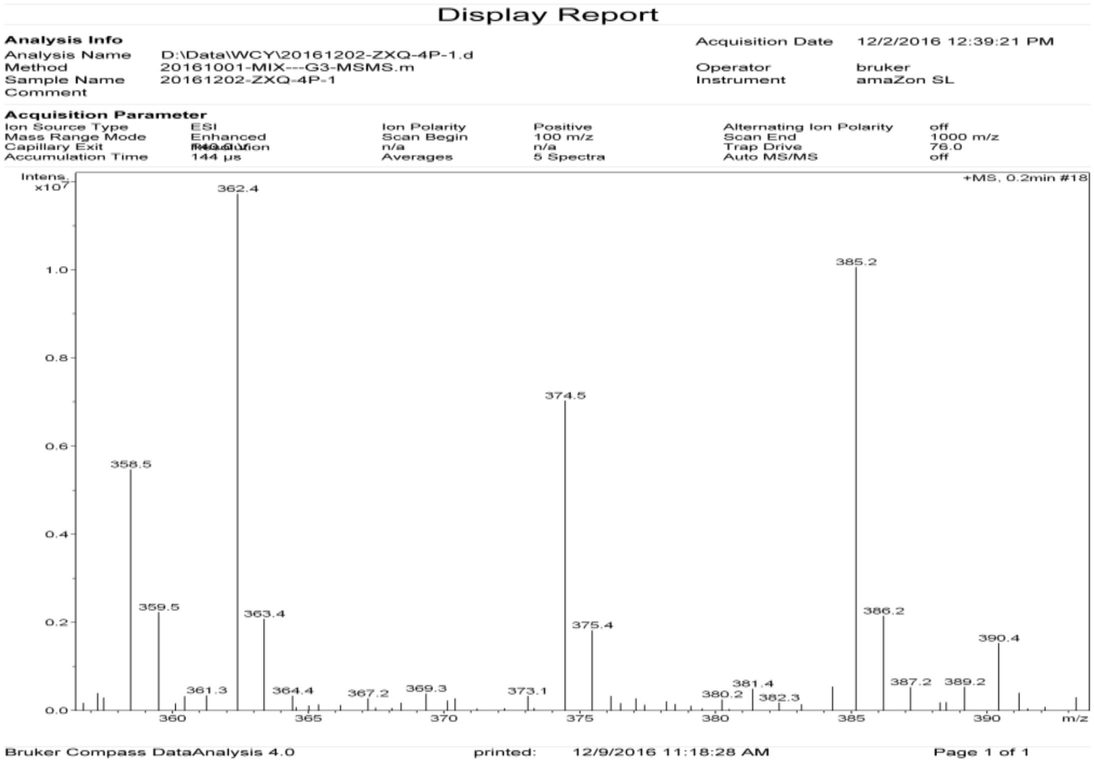

Figure S11. ESIMS spectrum of compound **4**.

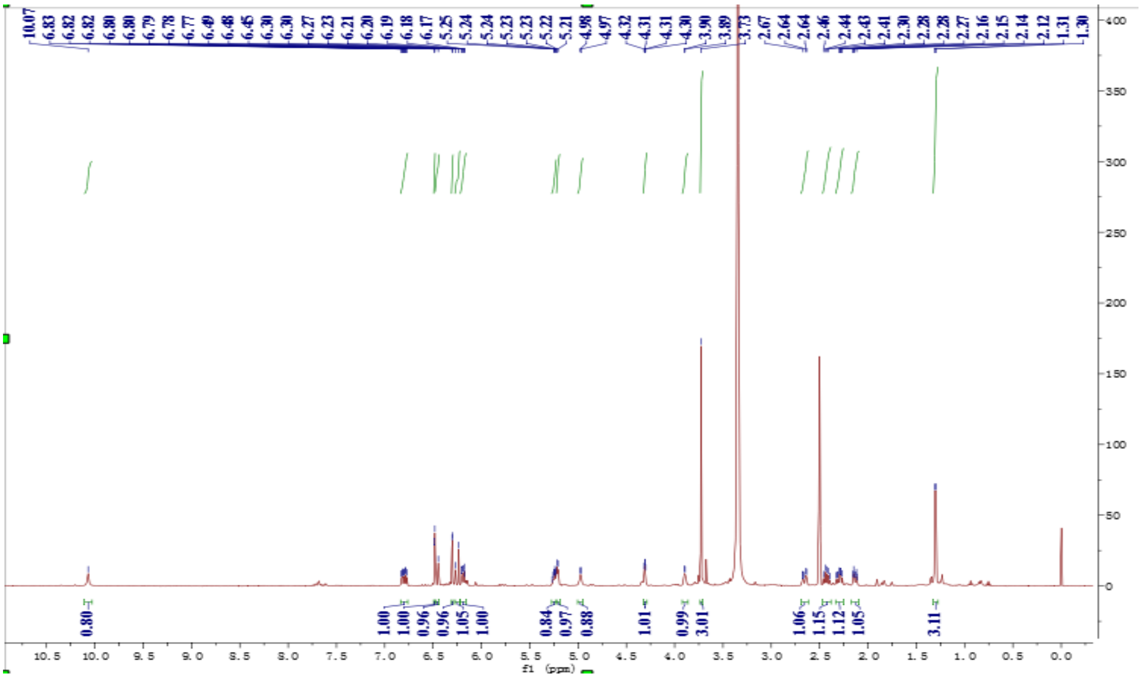

Figure S12. ^{1}H NMR (500 MHz, DMSO-d_6) spectrum of compound **5**.

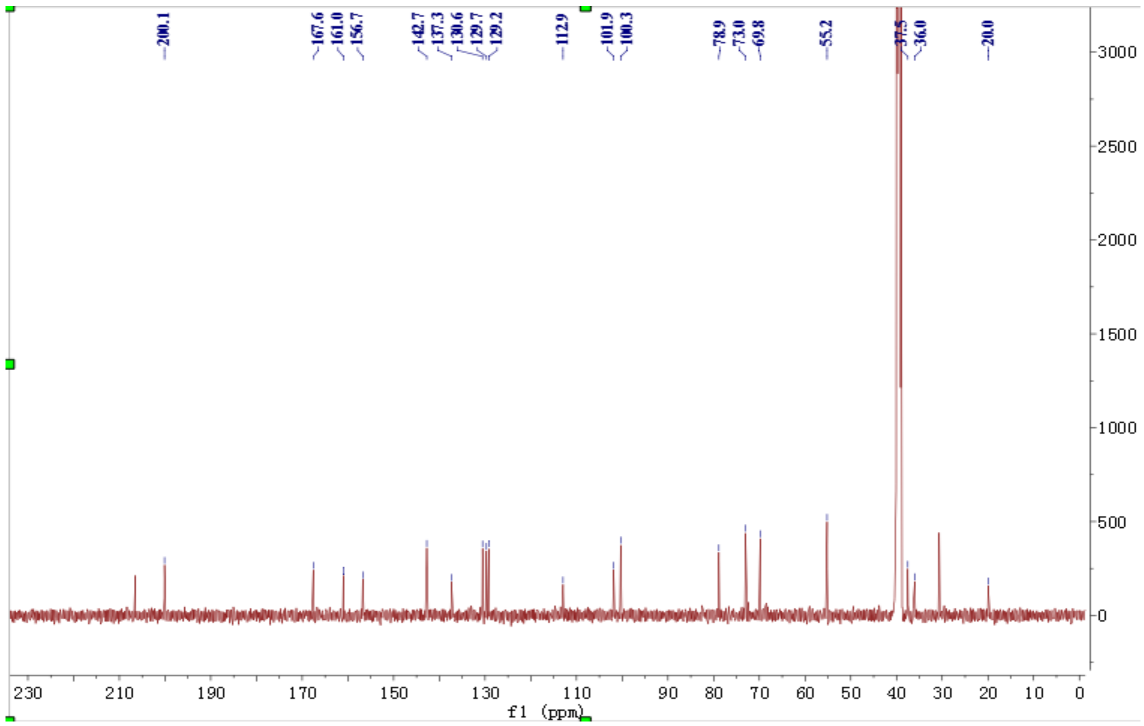

Figure S13. ^{13}C NMR (125 MHz, DMSO-d_6) spectrum of compound **5**.

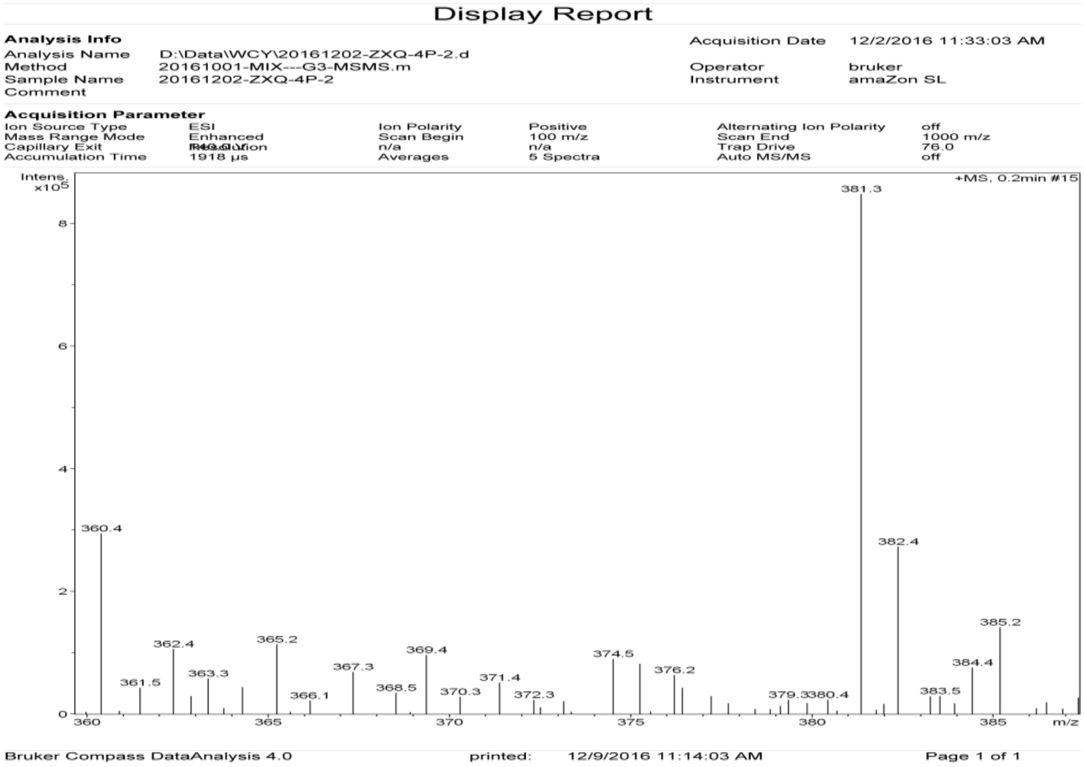

Figure S14. ESIMS spectrum of compound **5**.

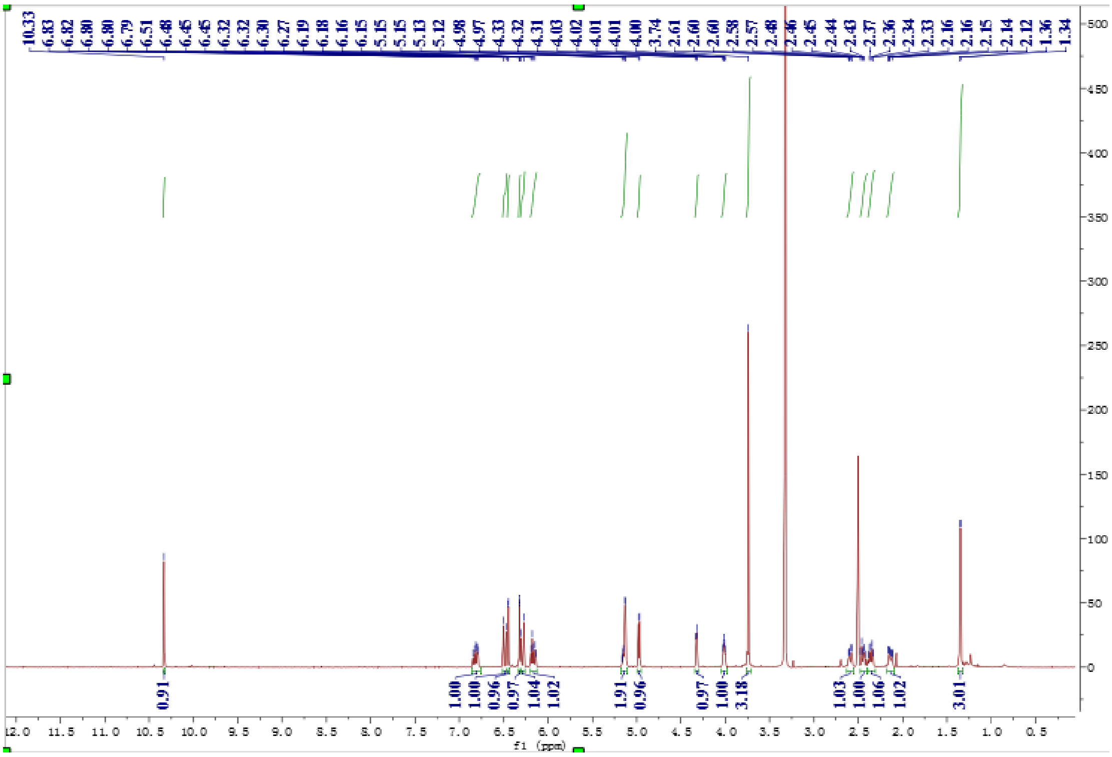

Figure S15. ^{1}H NMR (500 MHz, DMSO-d_6) spectrum of compound **6**.

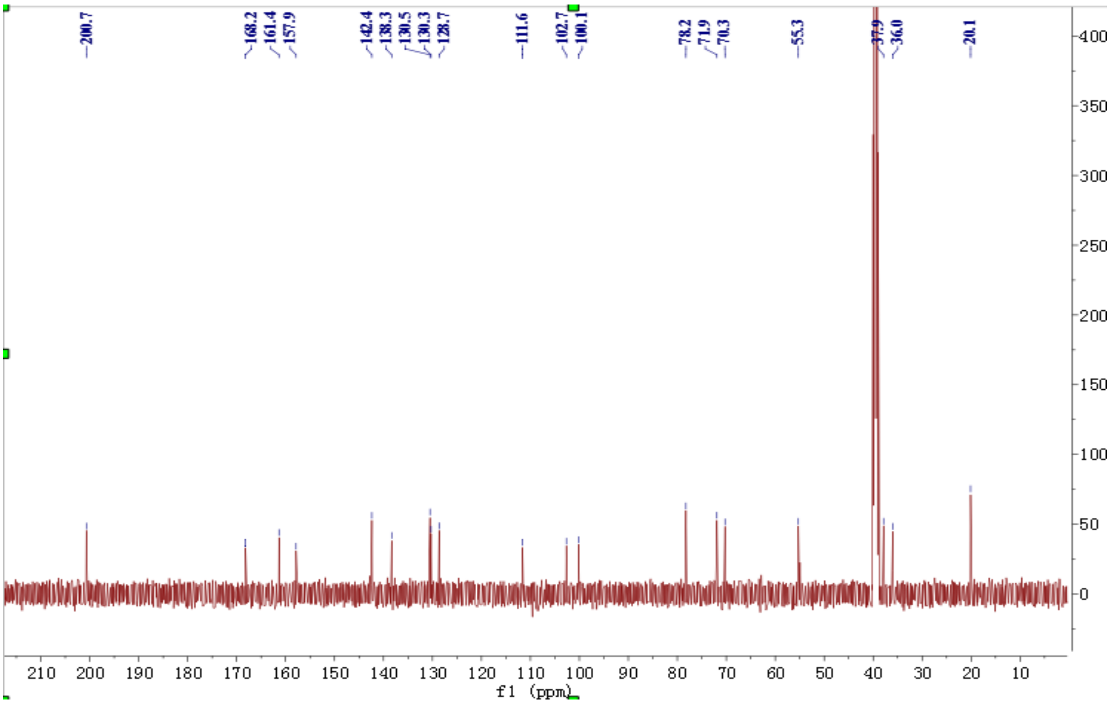

Figure S16. ^{13}C NMR (125 MHz, DMSO-d_6) spectrum of compound **6**.

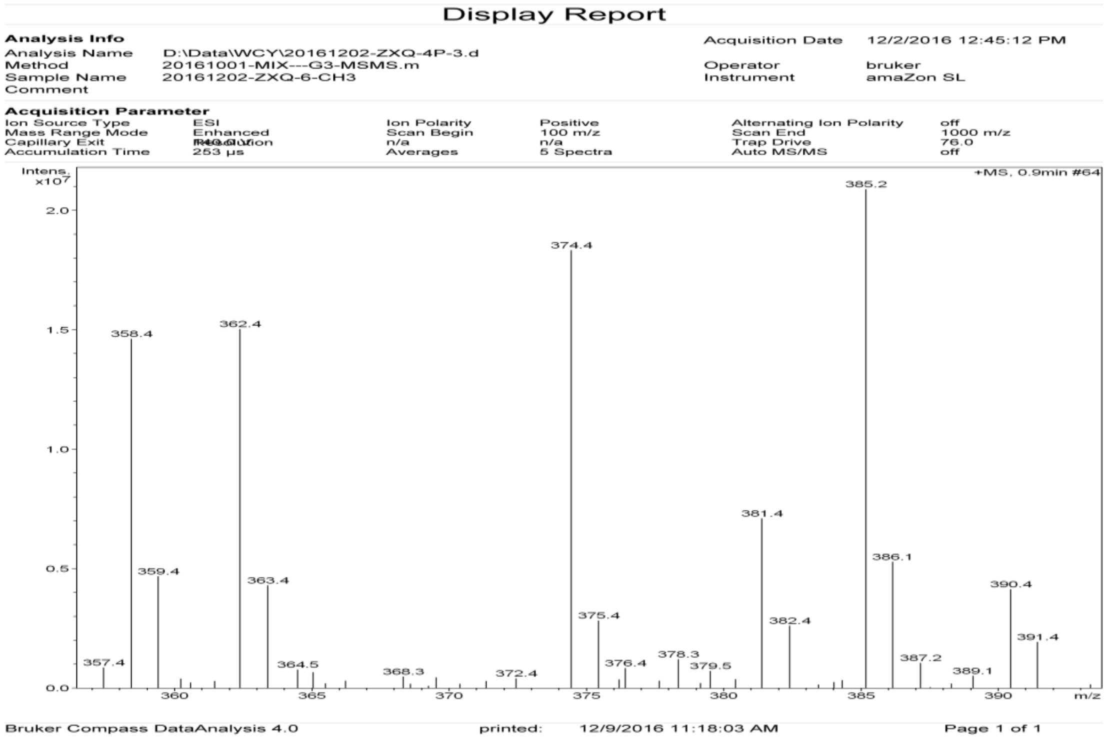

Figure S17. ESIMS spectrum of compound **6**.

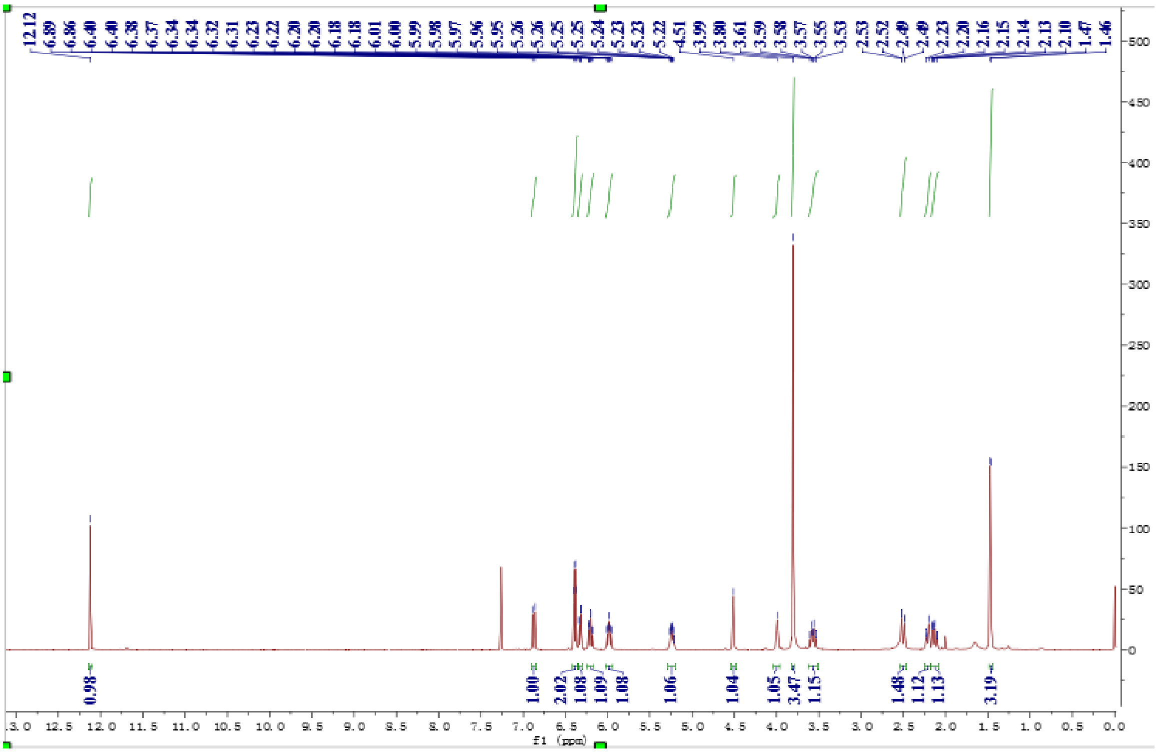

Figure S18. ^{1}H NMR (500 MHz, $CDCl_3$) spectrum of compound **7**.

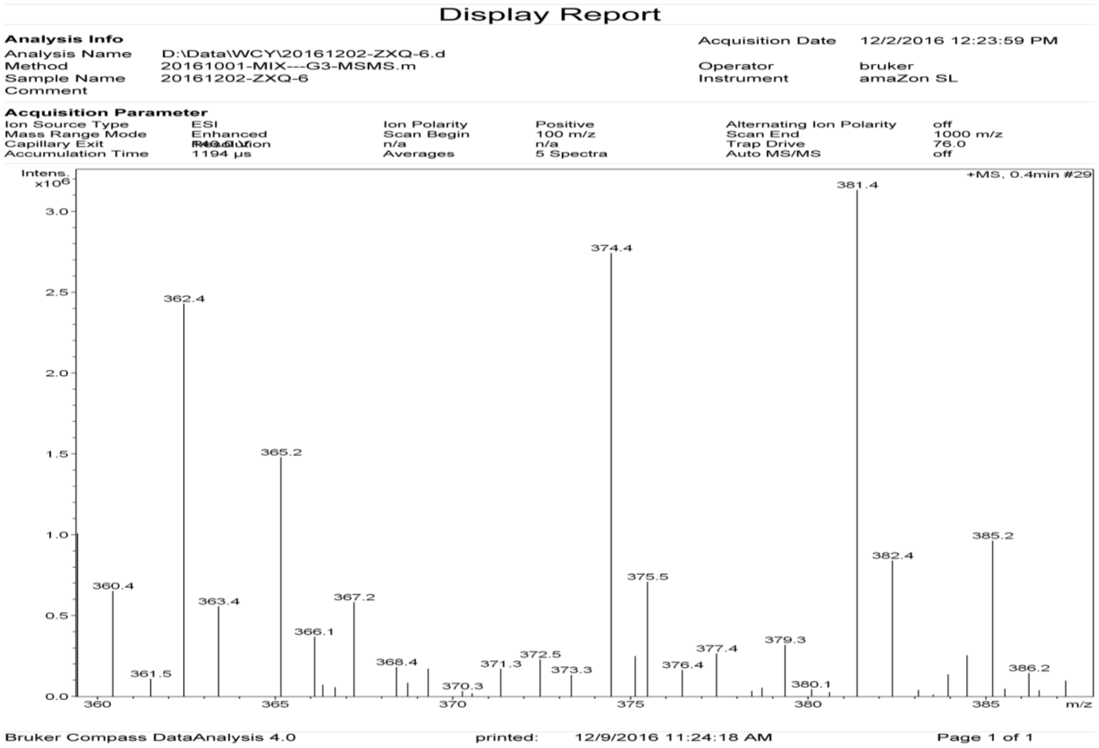

Figure S19. ESIMS spectrum of compound **7**.

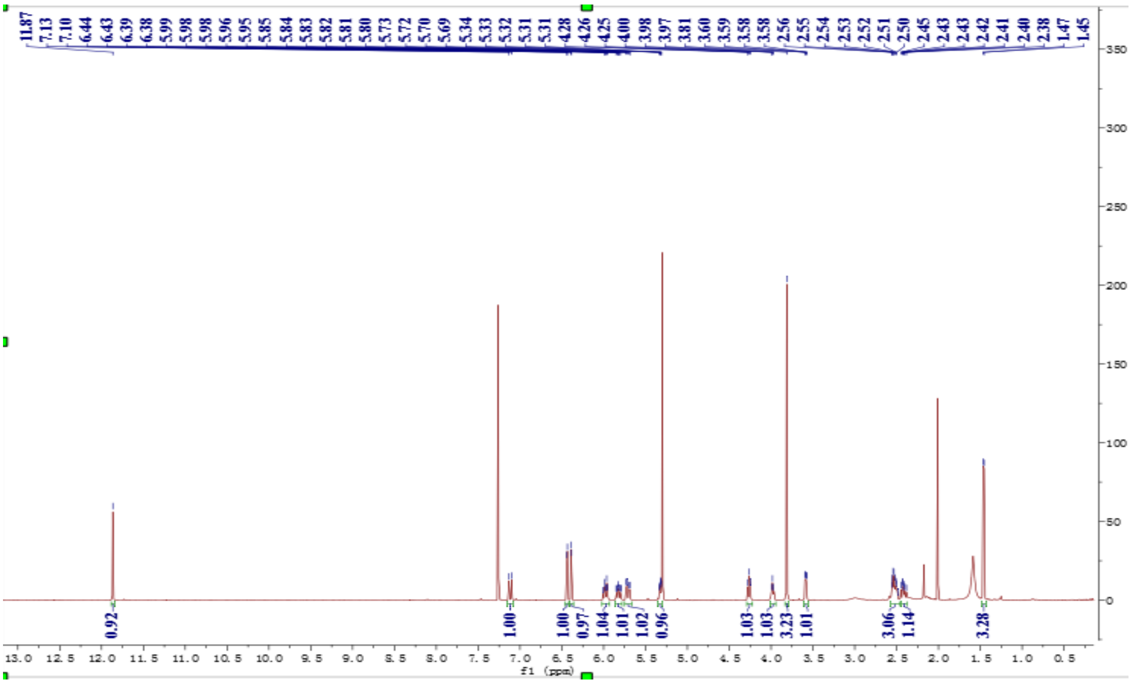

Figure S20. ^{1}H NMR (500 MHz, $CDCl_3$) spectrum of compound **8**.

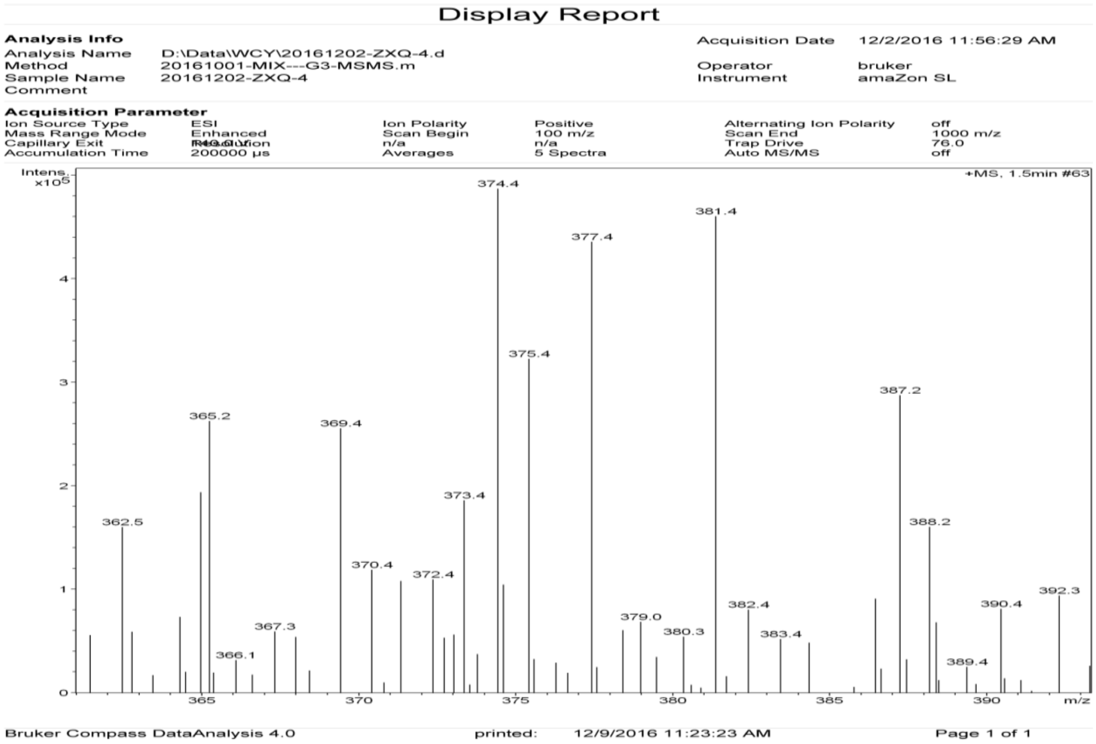

Figure S21. ESIMS spectrum of compound **8**.

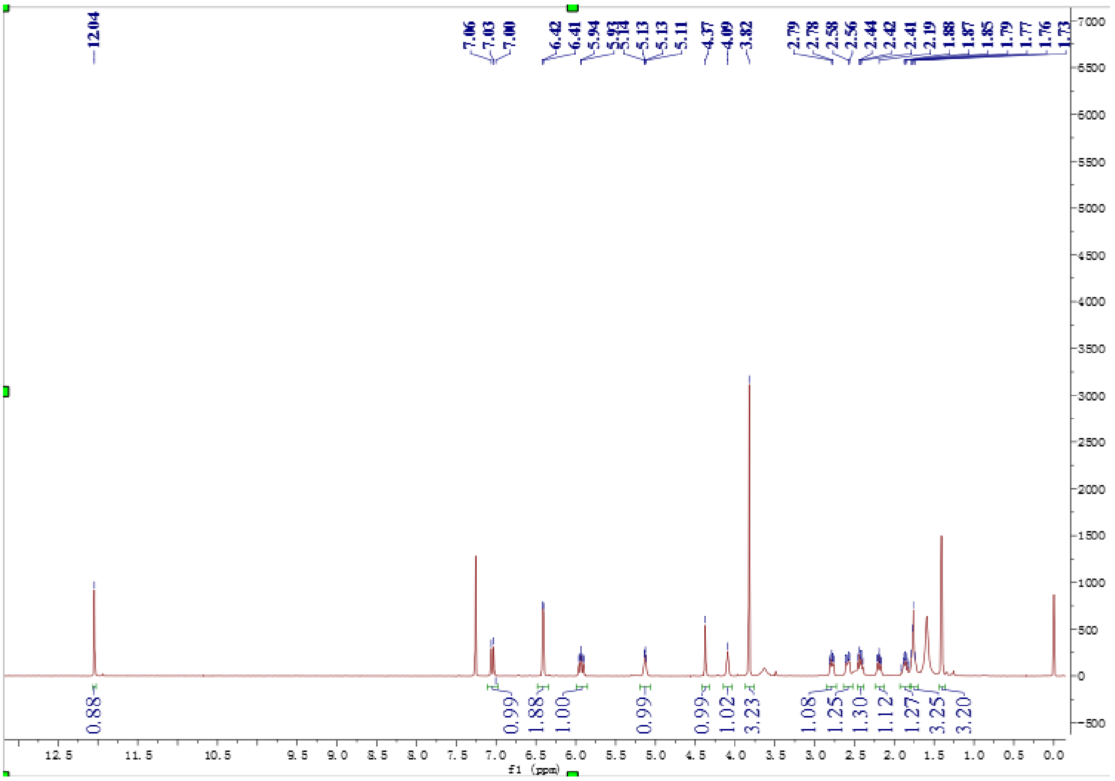

Figure S22. ^{1}H NMR (500 MHz, $CDCl_3$) spectrum of compound **9**.

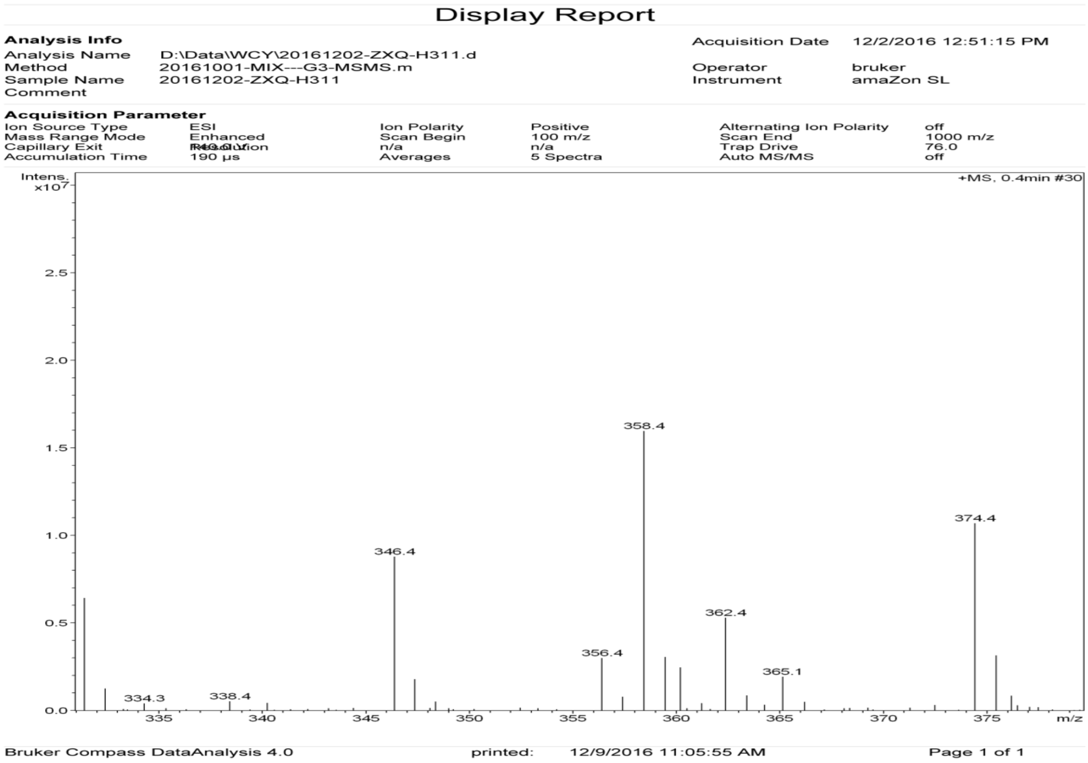

Figure S23. ESIMS spectrum of compound **9**.

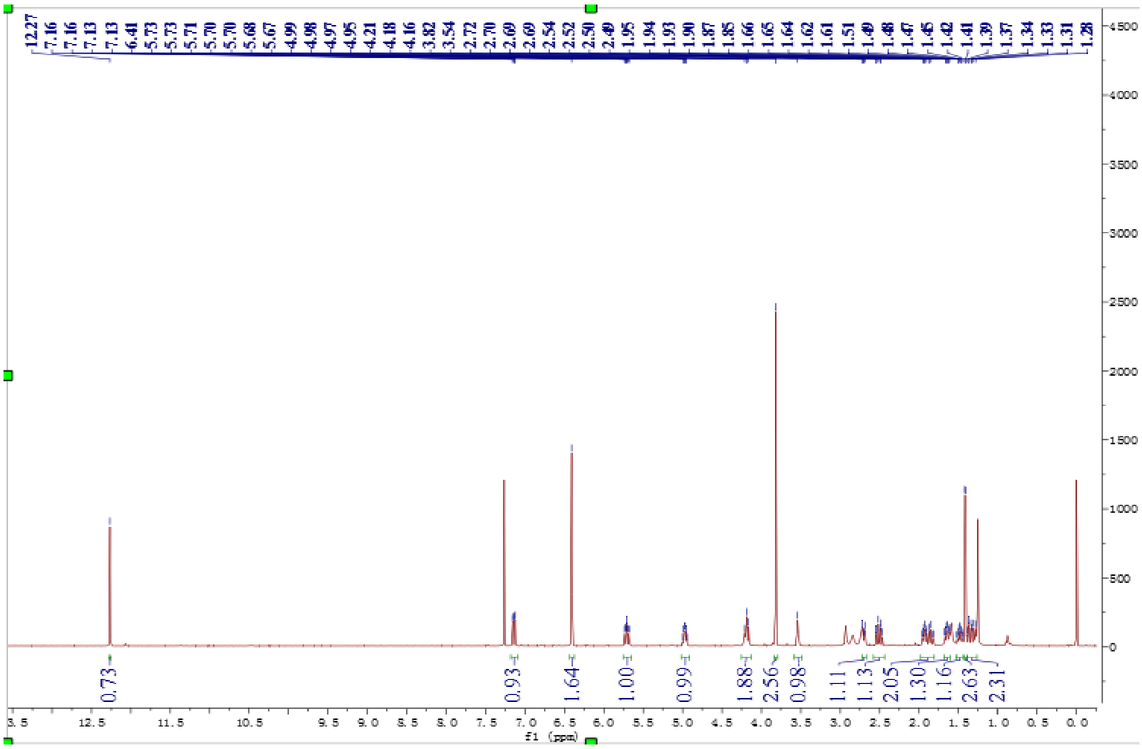

Figure S24. ^{1}H NMR (500 MHz, $CDCl_3$) spectrum of compound **10**.

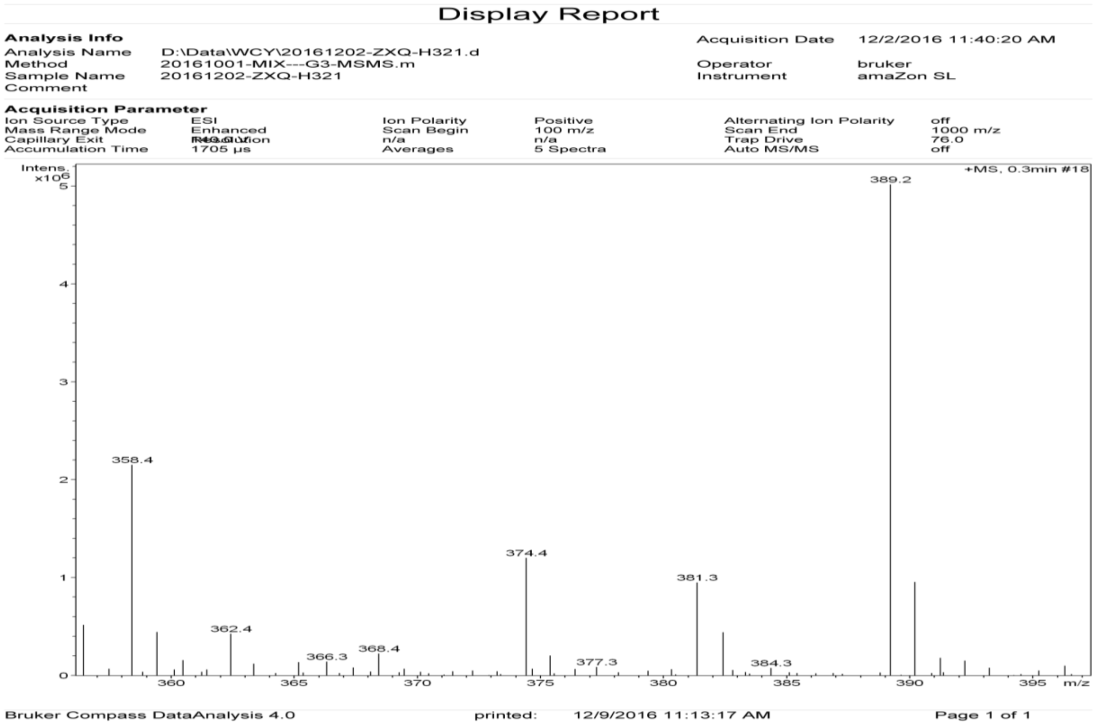

Figure S25. ESIMS spectrum of compound **10**.

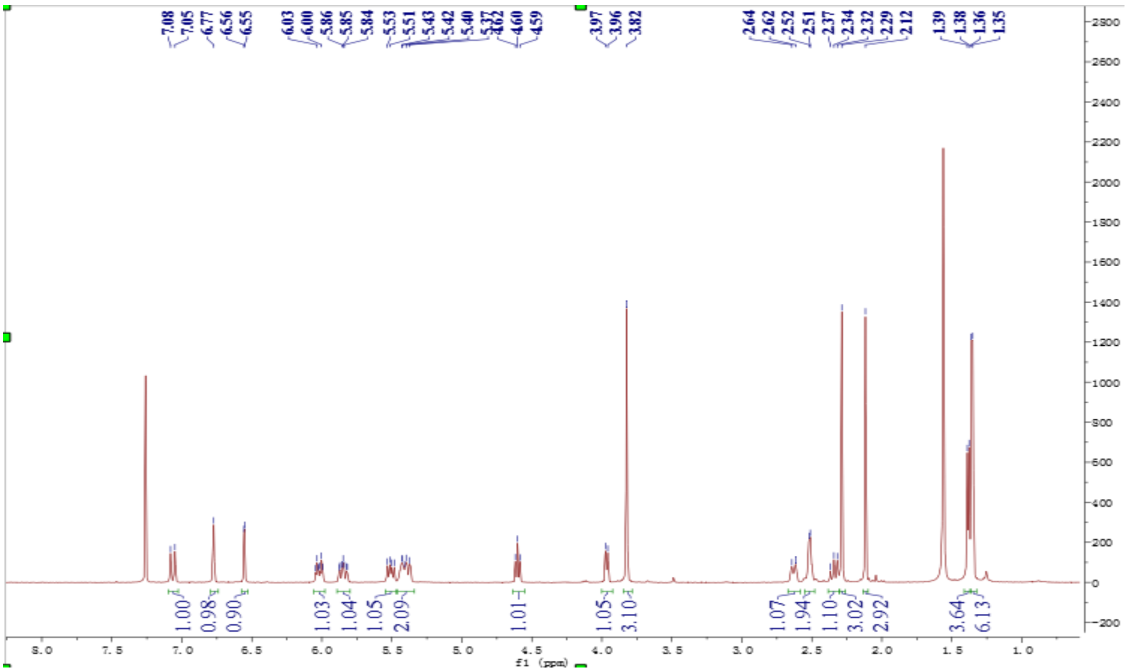

Figure S26. ^{1}H NMR (500 MHz, $CDCl_3$) spectrum of compound **11**.

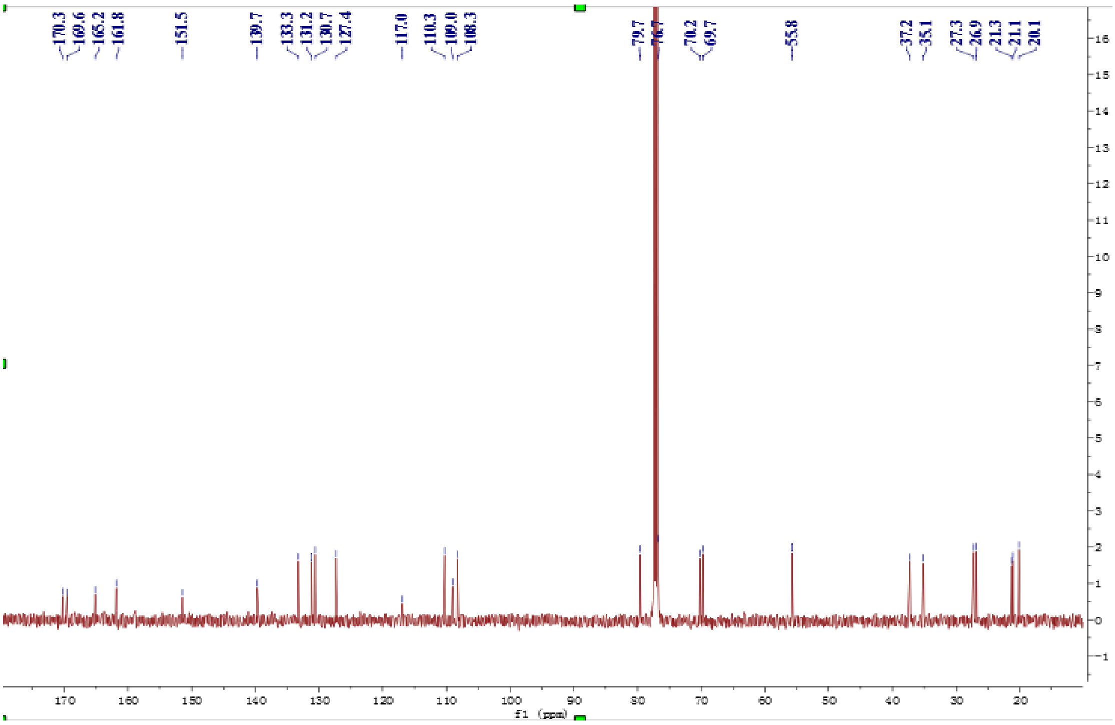

Figure S27. ^{13}C NMR (125 MHz, $CDCl_3$) spectrum of compound **11**.

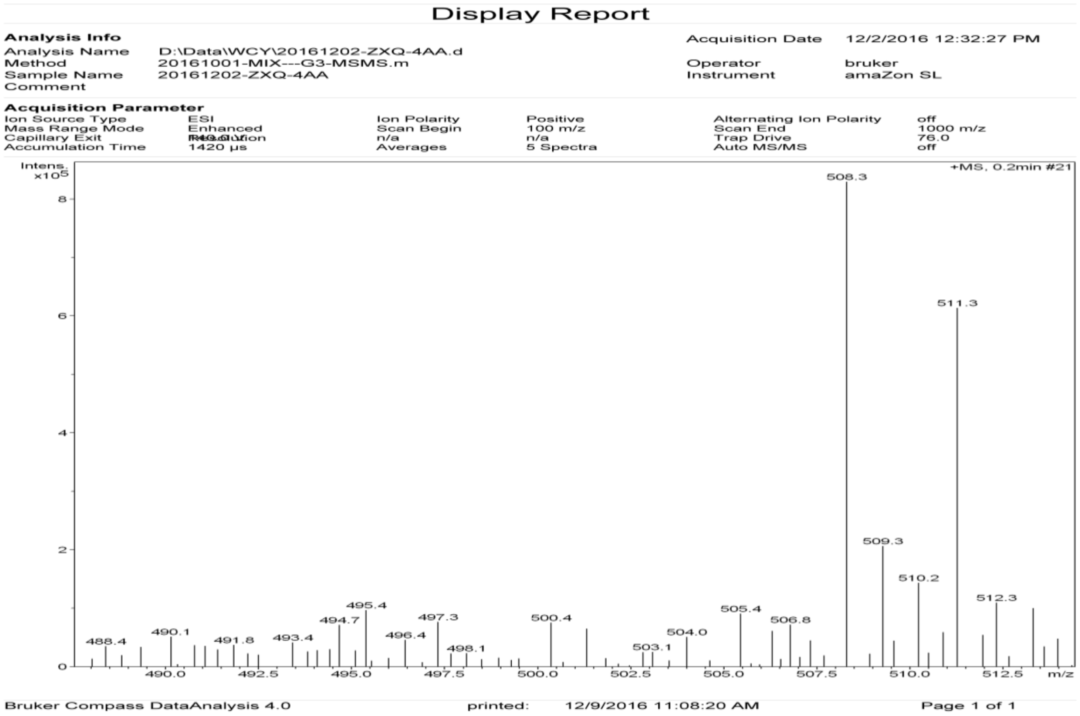

Figure S28. ESIMS spectrum of compound **11**.

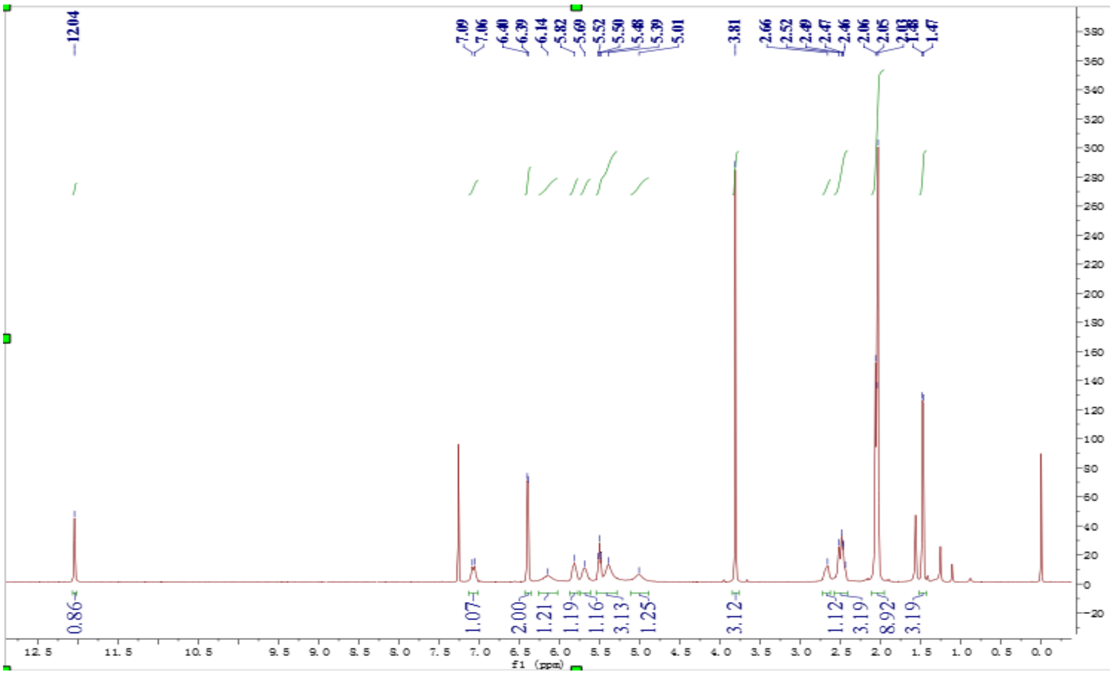

Figure S29. ^{1}H NMR (500 MHz, $CDCl_3$) spectrum of compound **12**.

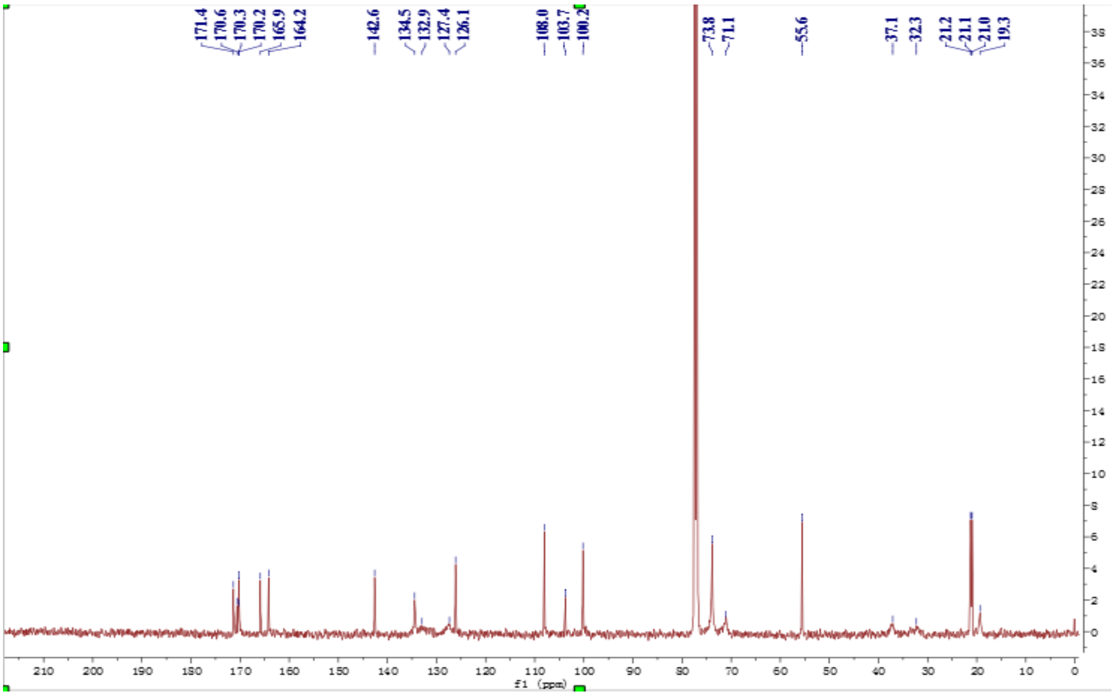

Figure S30. ^{13}C NMR (125 MHz, $CDCl_3$) spectrum of compound **12.**

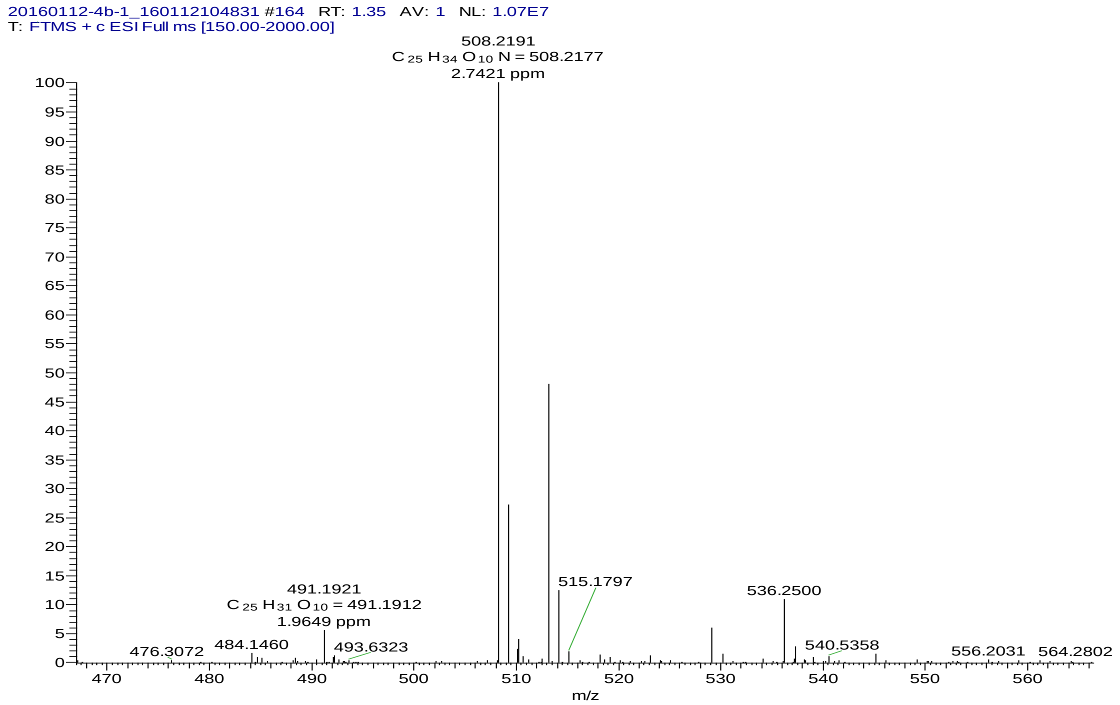

Figure S31. HRESIMS spectrum of compound **12**.

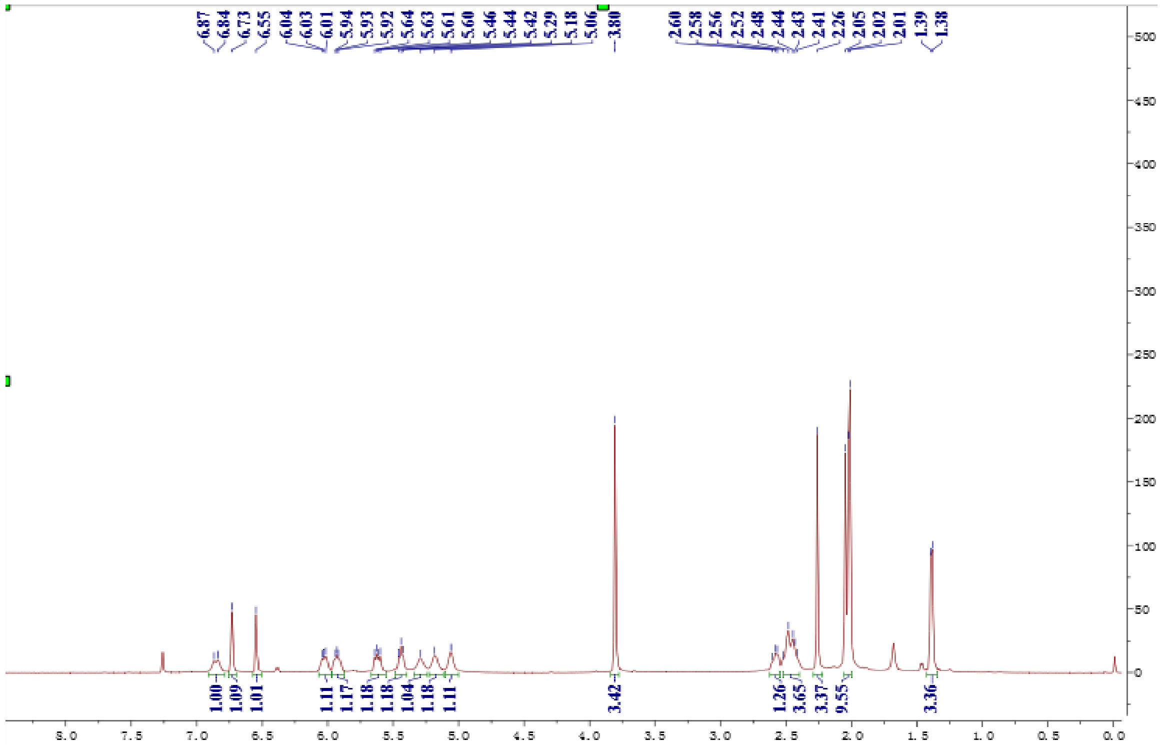

Figure S32. ^{1}H NMR (500 MHz, $CDCl_3$) spectrum of compound **13**.

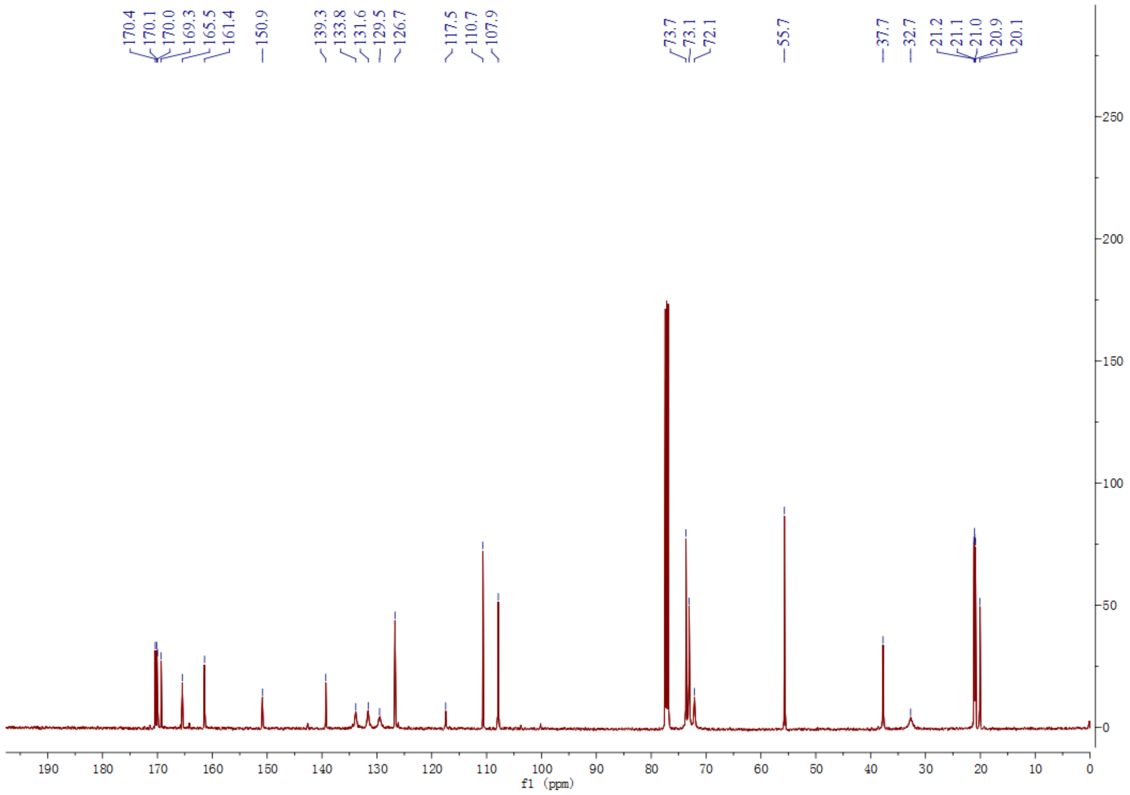

Figure S33. ^{13}C NMR (125 MHz, $CDCl_3$) spectrum of compound **13**.

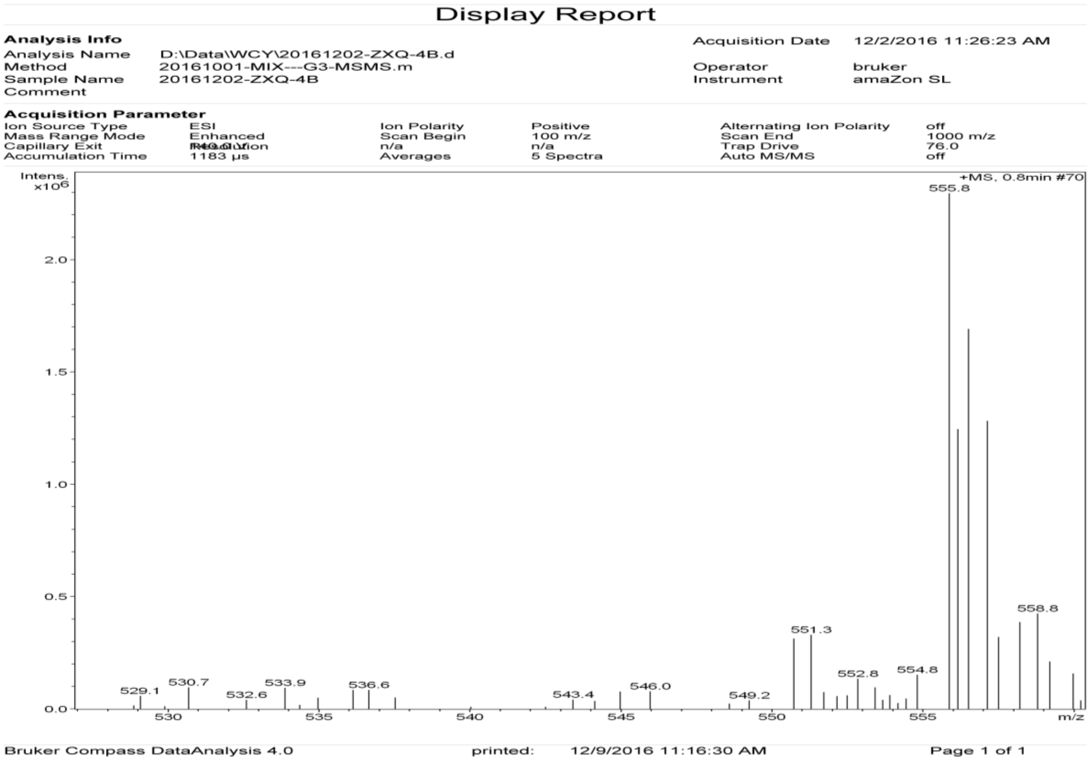

Figure S34. ESIMS spectrum of compound **13**.

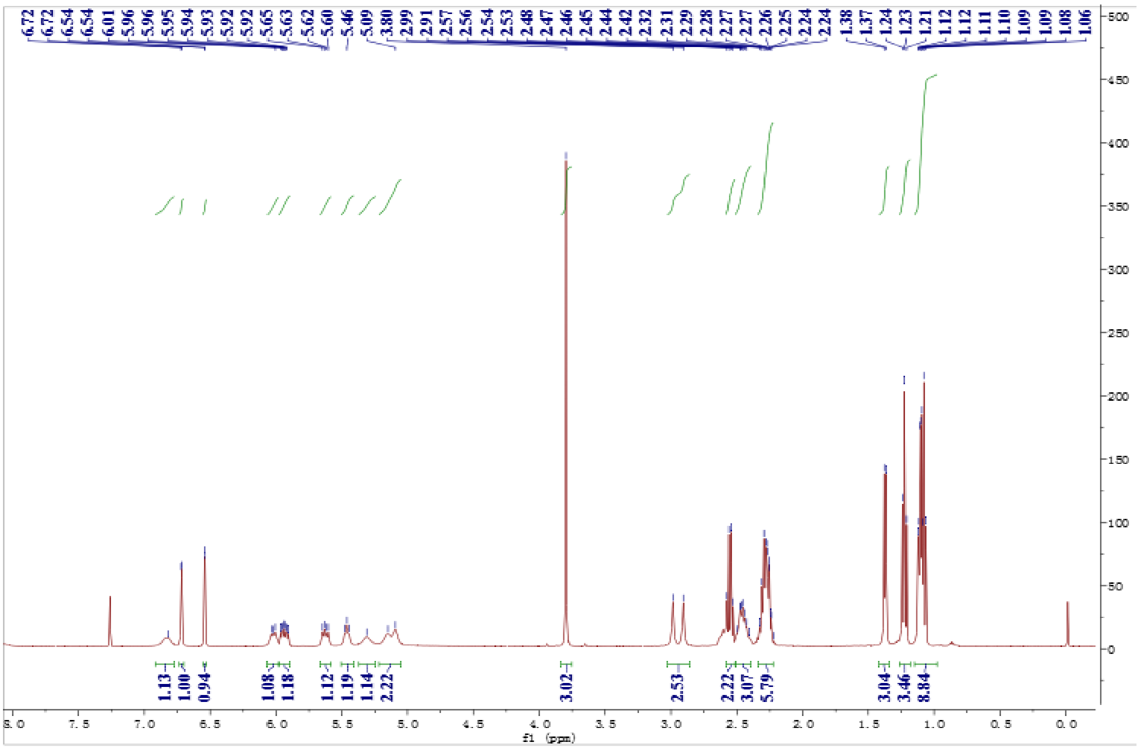

Figure S35. ^{1}H NMR (500 MHz, $CDCl_3$) spectrum of compound **14**.

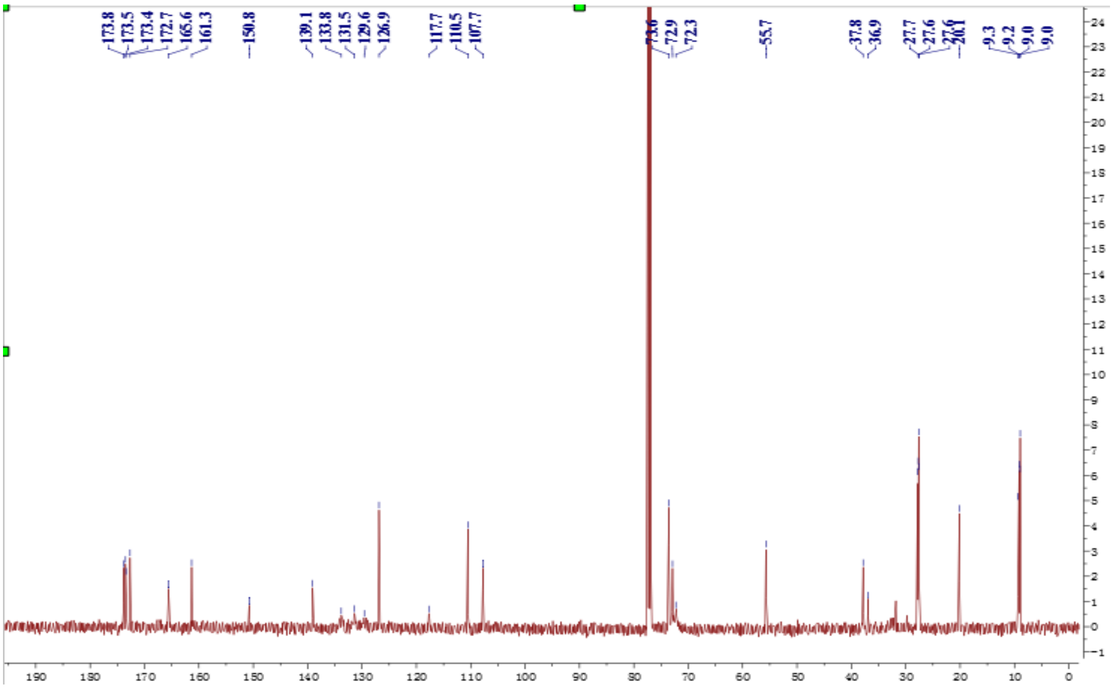

Figure S36. ^{13}C NMR (125 MHz, $CDCl_3$) spectrum of compound **14**.

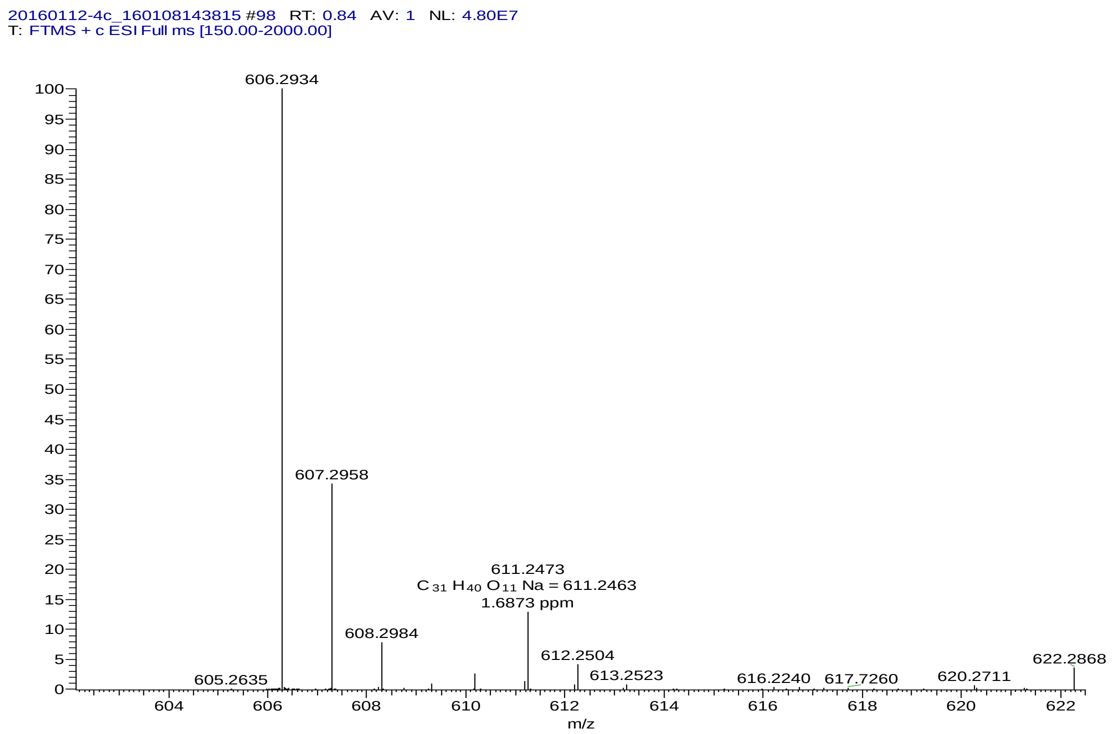

Figure S37. HRESIMS spectrum of compound **14**.

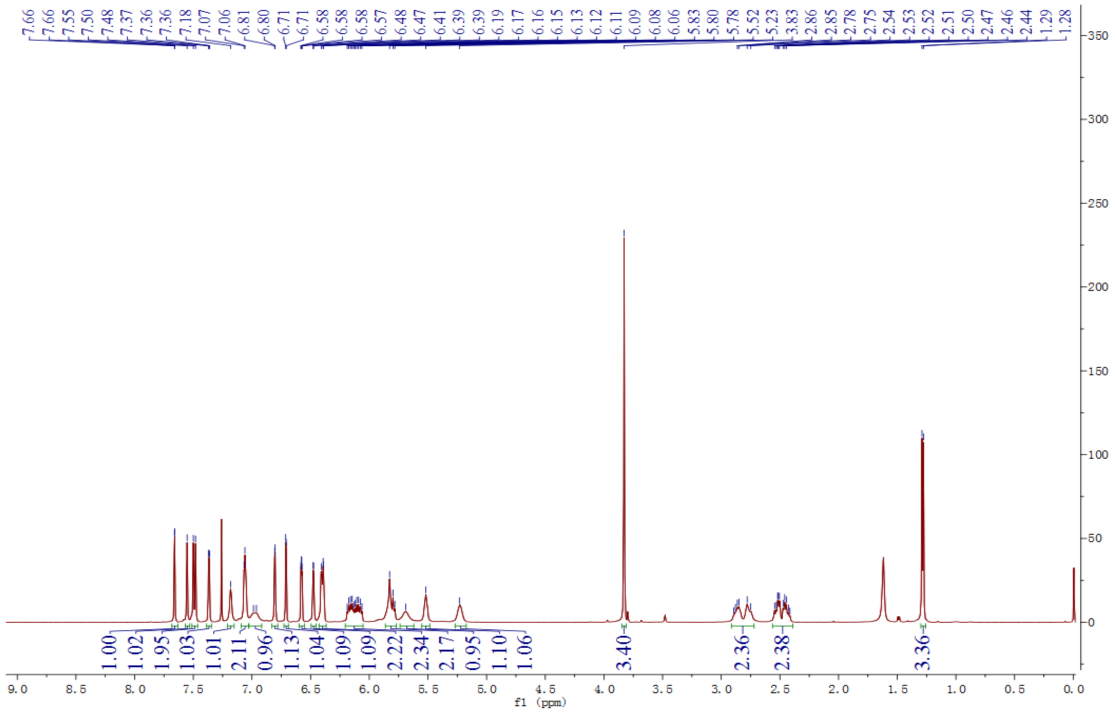

Figure S38. ^{1}H NMR (500 MHz, $CDCl_3$) spectrum of compound **15**.

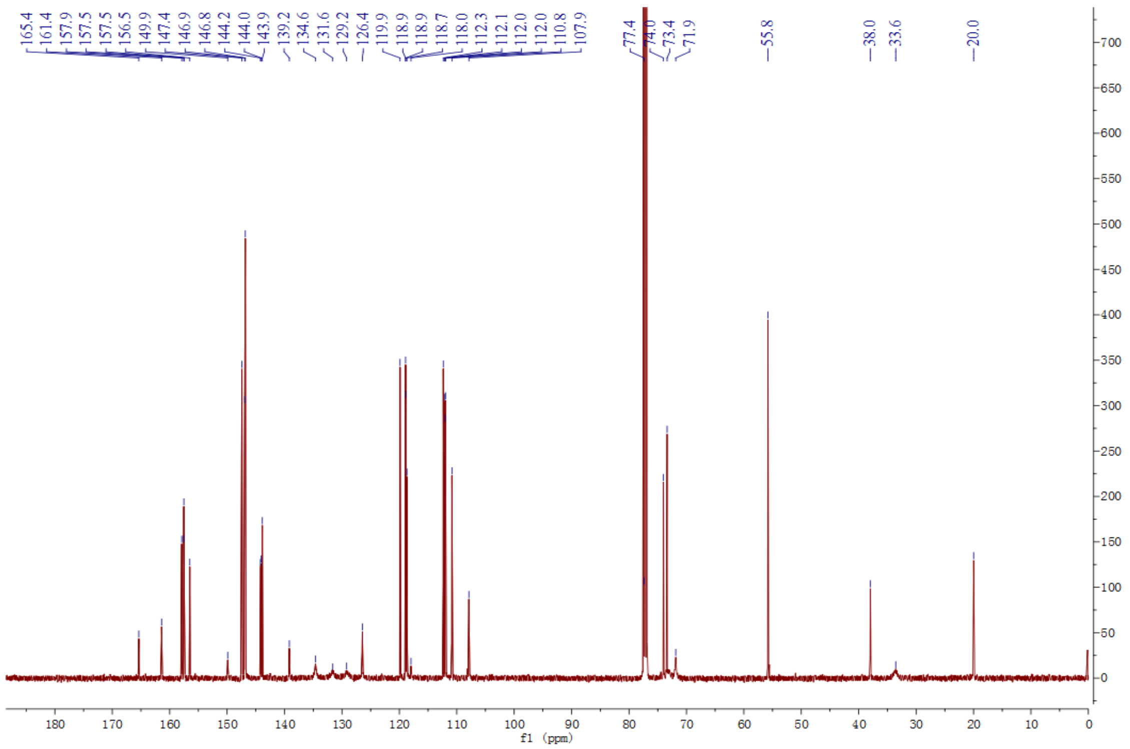

Figure S39. ^{13}C NMR (125 MHz, $CDCl_3$) spectrum of compound **15**.

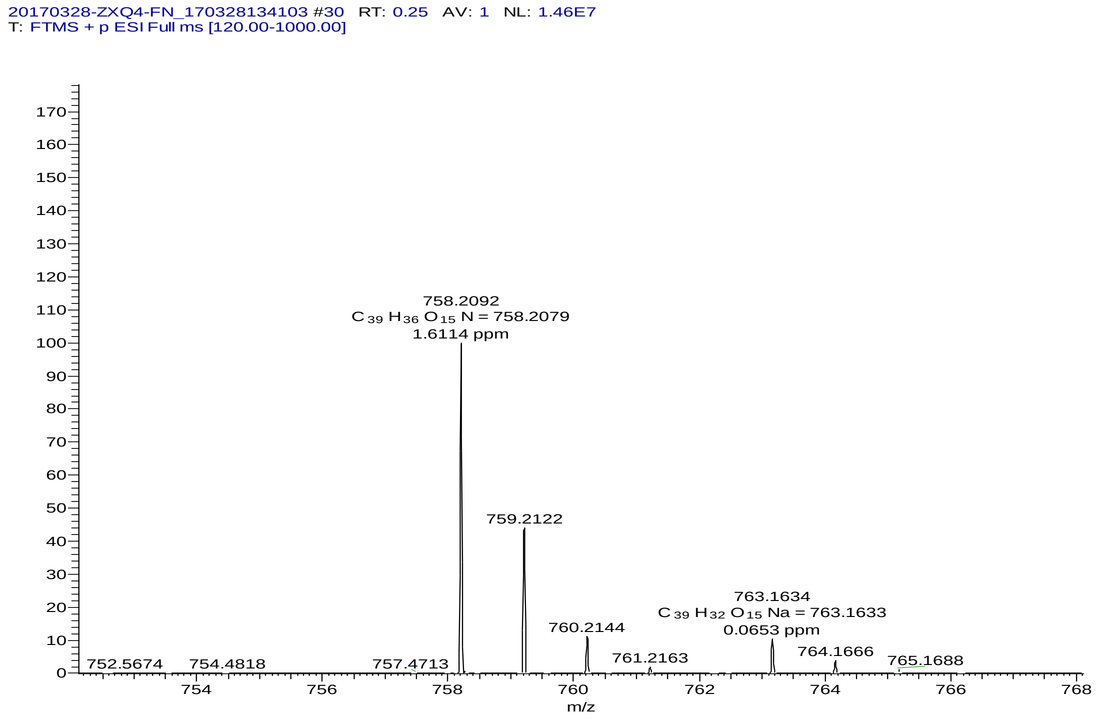

Figure S40. HRESIMS spectrum of compound **15**.

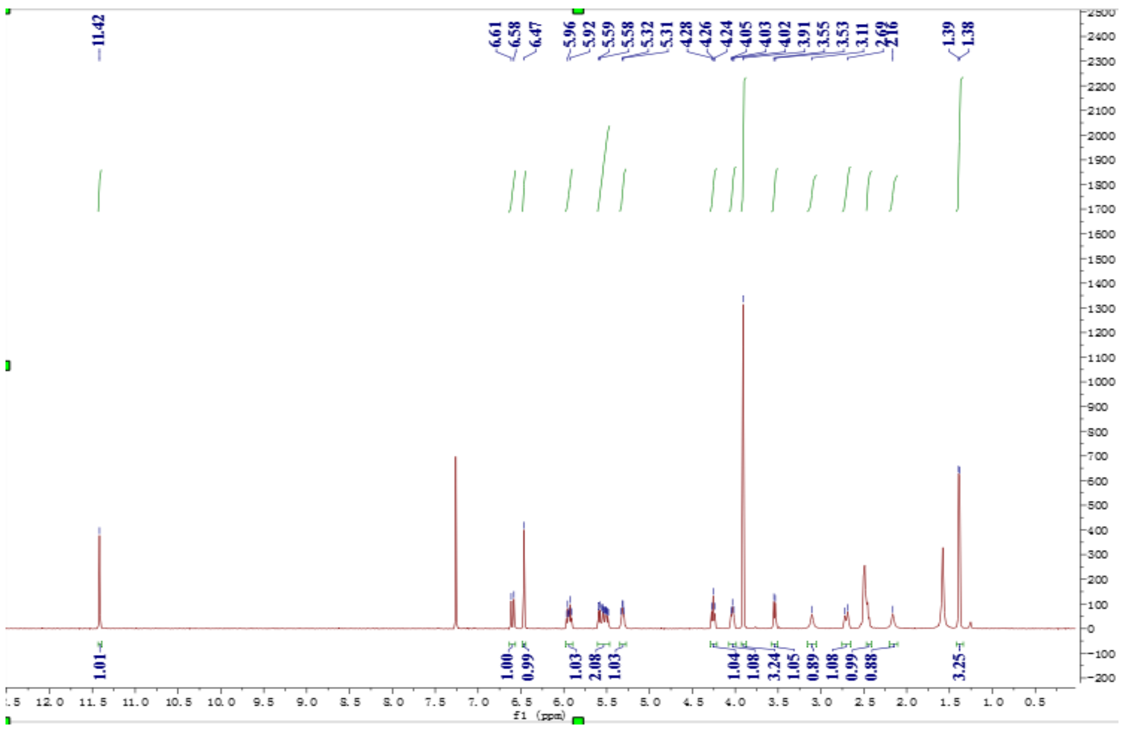

Figure S41. ^{1}H NMR (500 MHz, $CDCl_3$) spectrum of compound **16**.

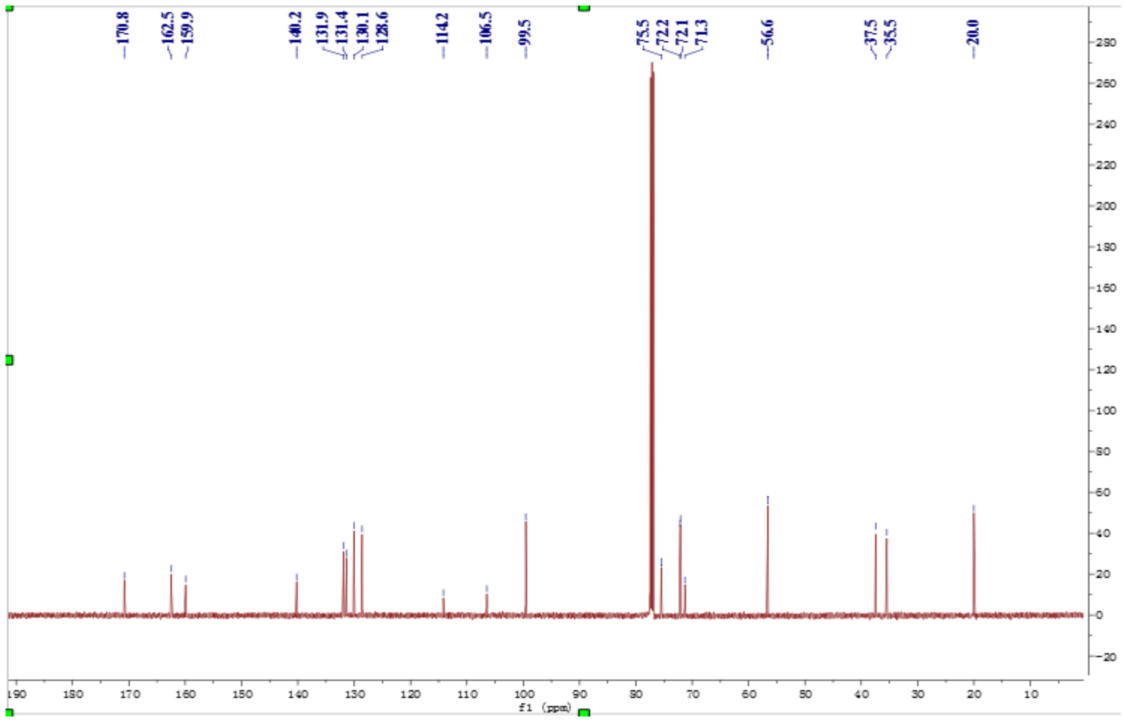

Figure S42. ^{13}C NMR (125 MHz, $CDCl_3$) spectrum of compound **16**.

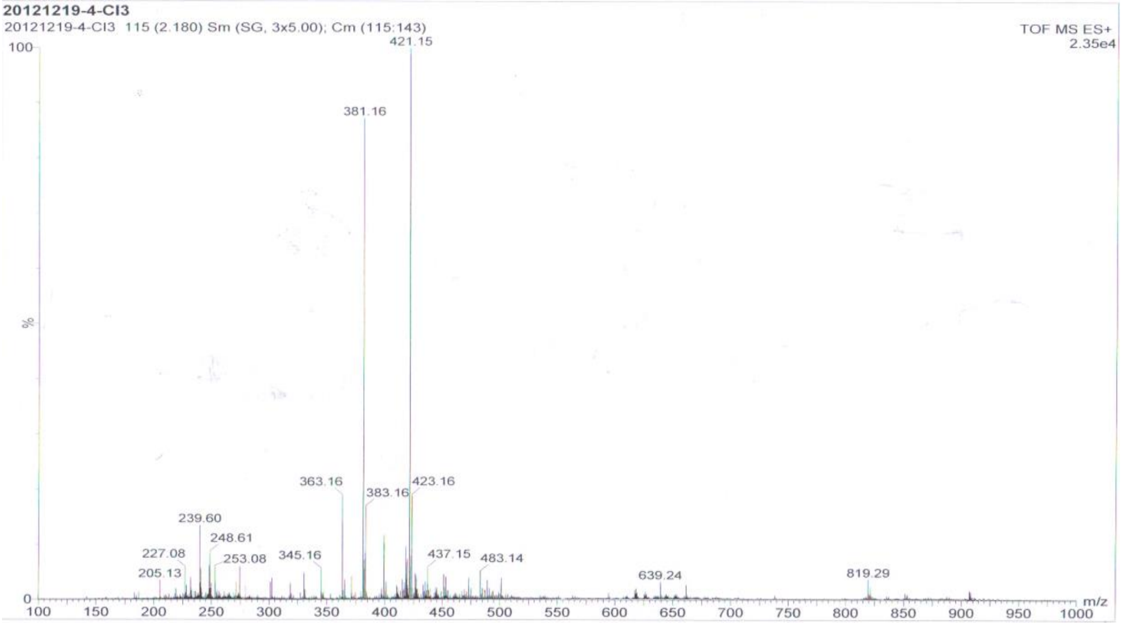

Figure S43. ESIMS spectrum of compound **16**.

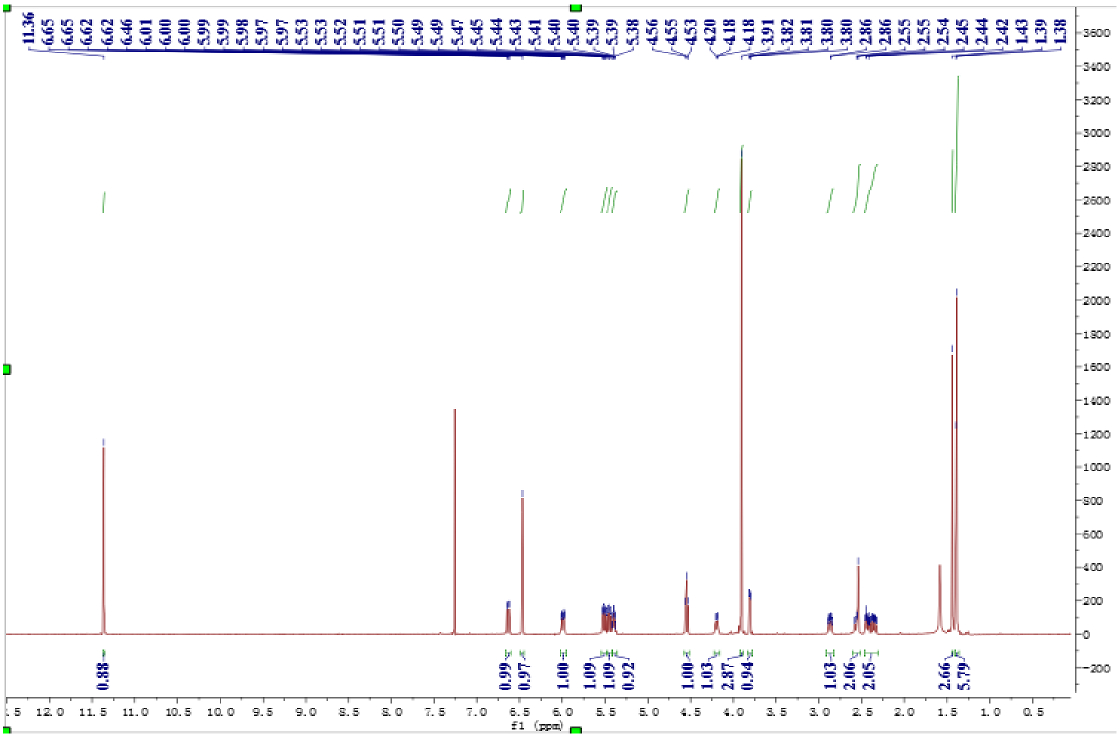

Figure S44. ^{1}H NMR (500 MHz, $CDCl_3$) spectrum of compound **17**.

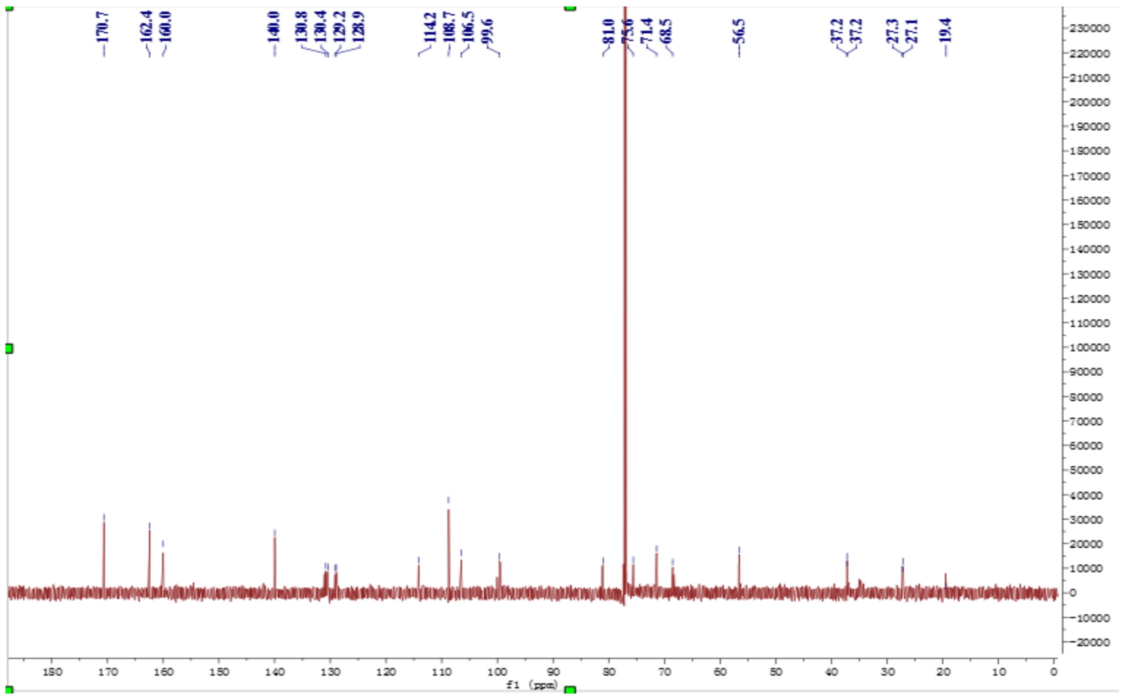

Figure S45. ^{13}C NMR (125 MHz, $CDCl_3$) spectrum of compound **17**.

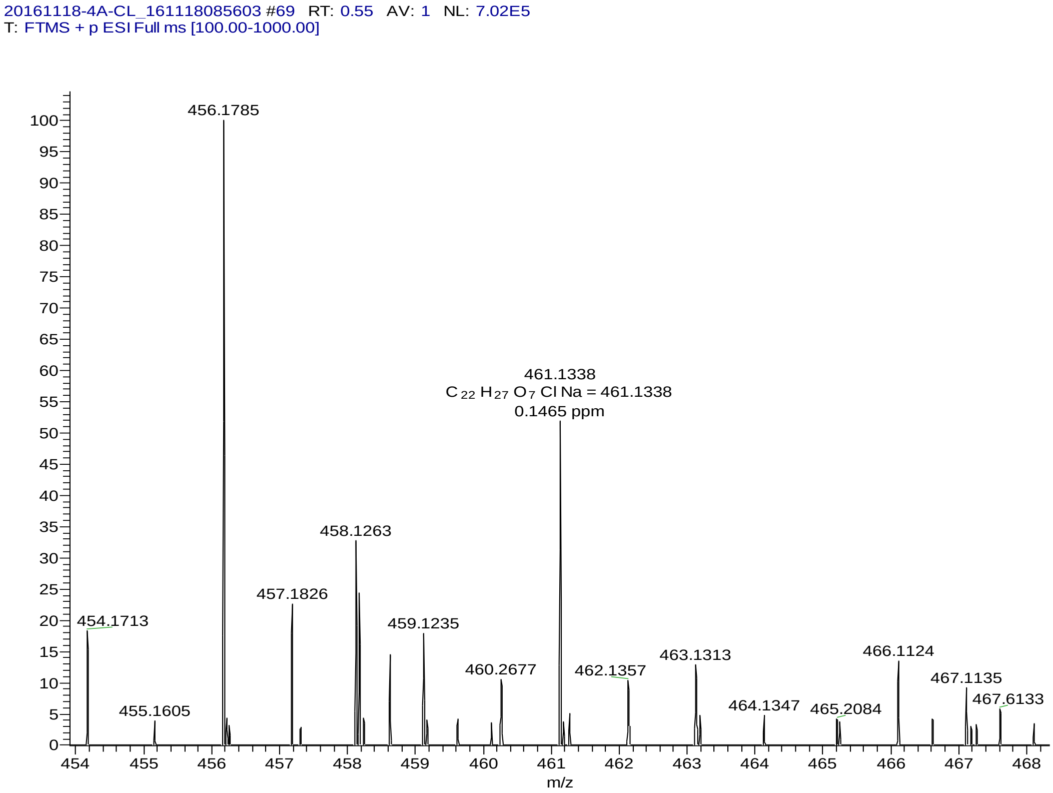

Figure S46. HRESIMS spectrum of compound **17**.

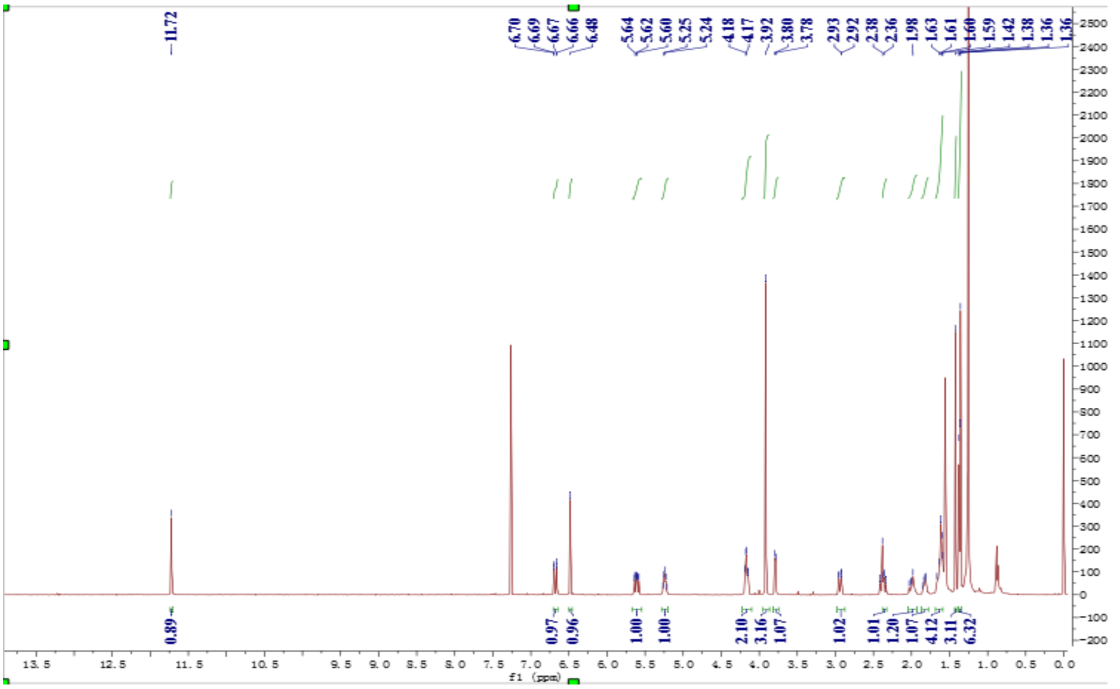

Figure S47. ^{1}H NMR (500 MHz, $CDCl_3$) spectrum of compound **18**.

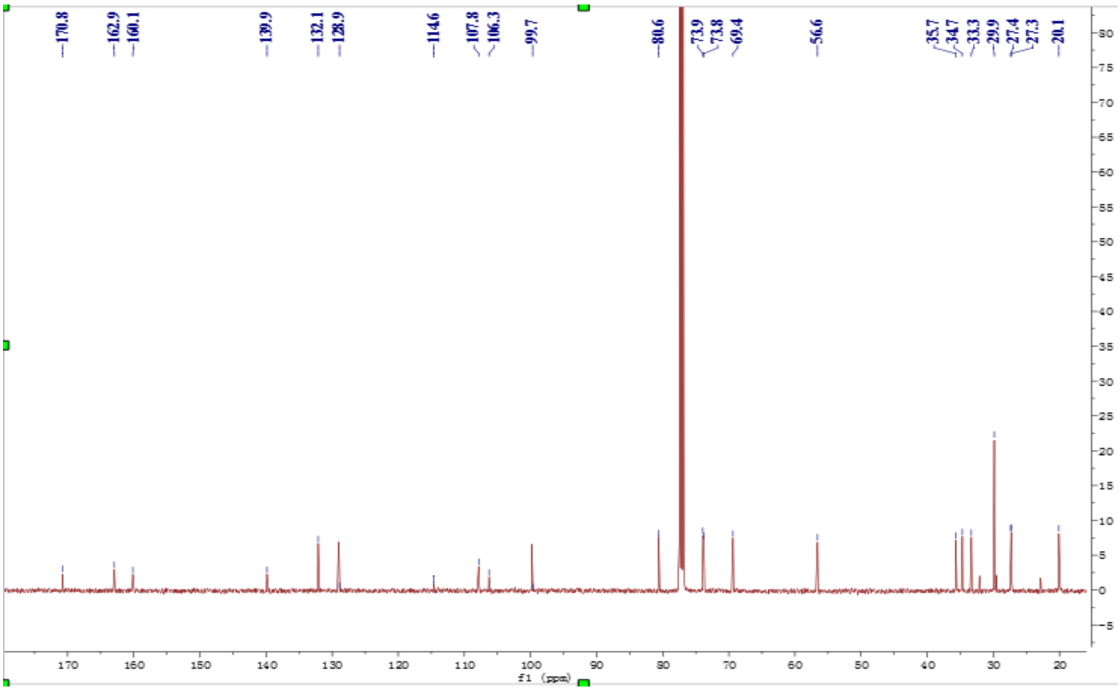

Figure S48. ^{13}C NMR (125 MHz, $CDCl_3$) spectrum of compound **18**.

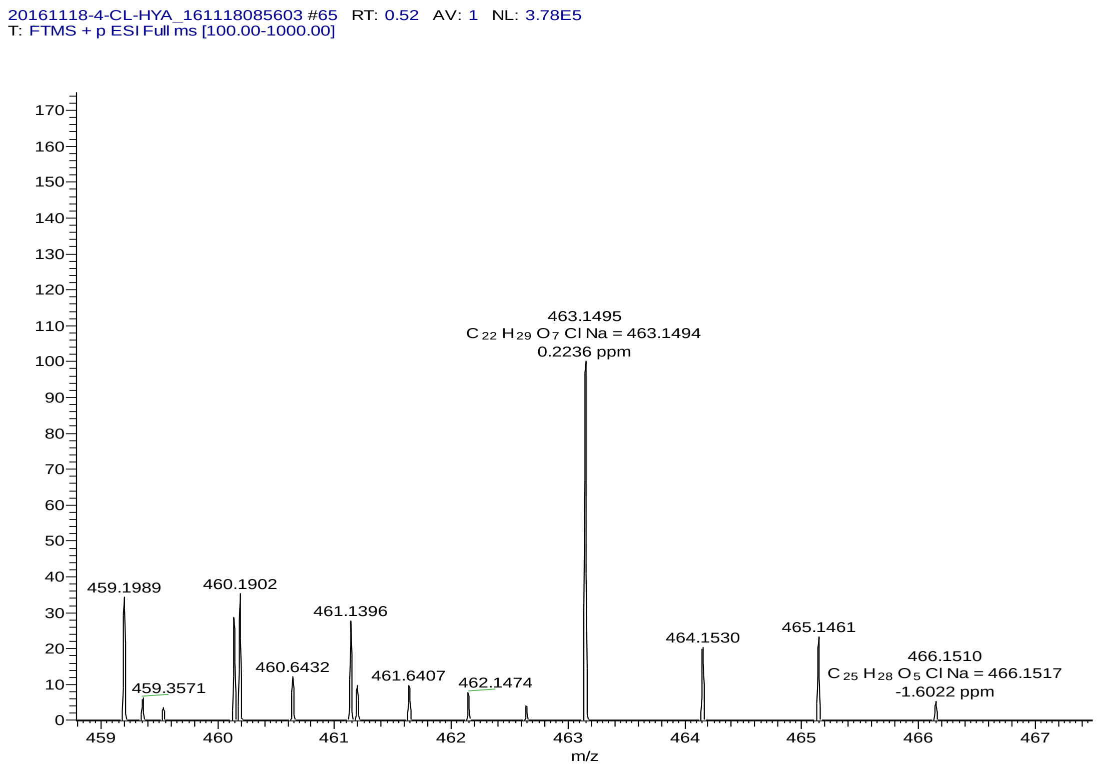

Figure S49. HRESIMS spectrum of compound **18**.

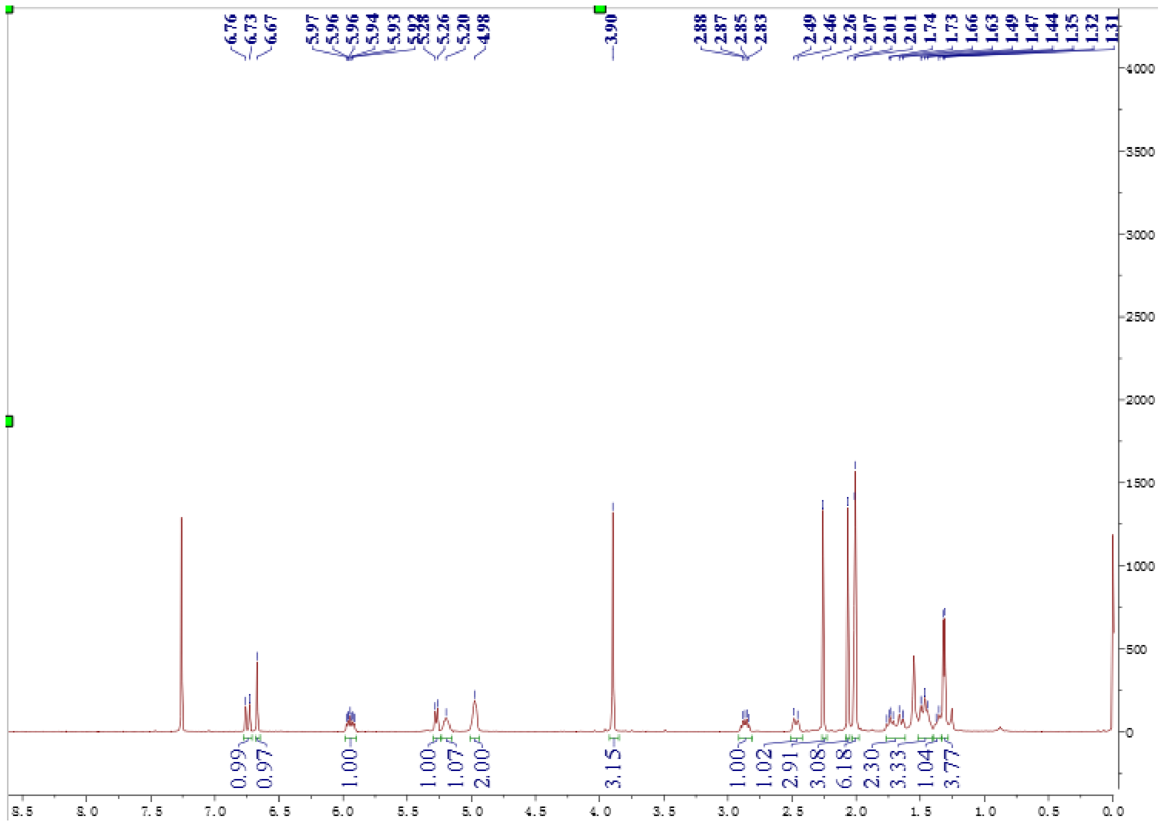

Figure S50. ^{1}H NMR (500 MHz, $CDCl_3$) spectrum of compound **19**.

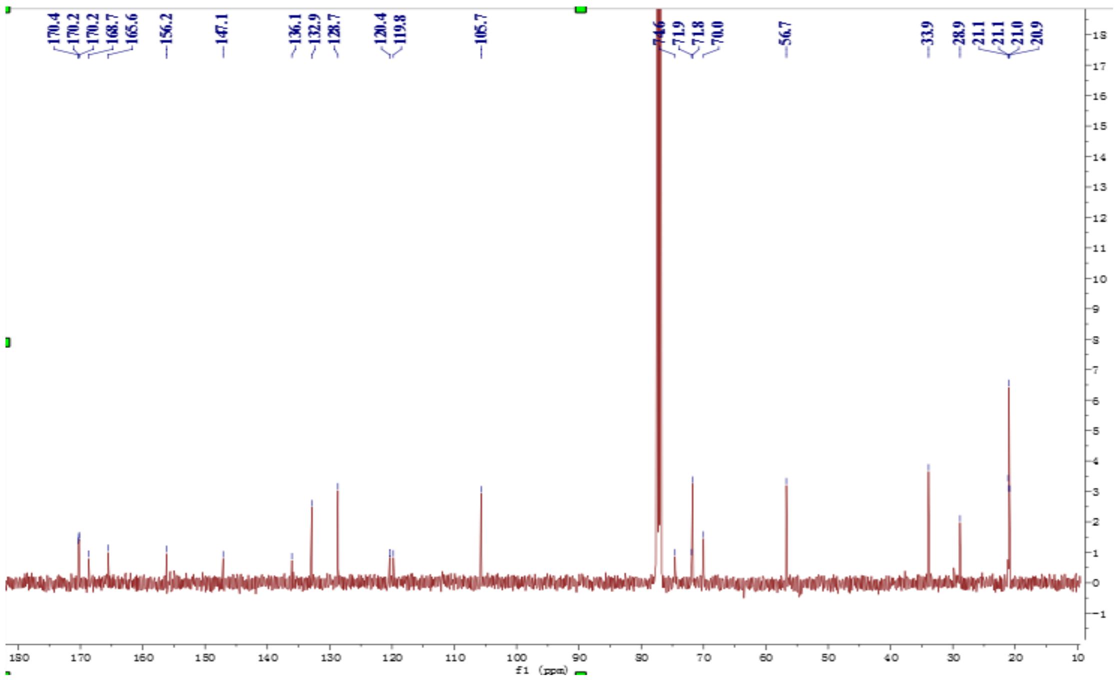

Figure S51. ^{13}C NMR (125 MHz, $CDCl_3$) spectrum of compound **19**.

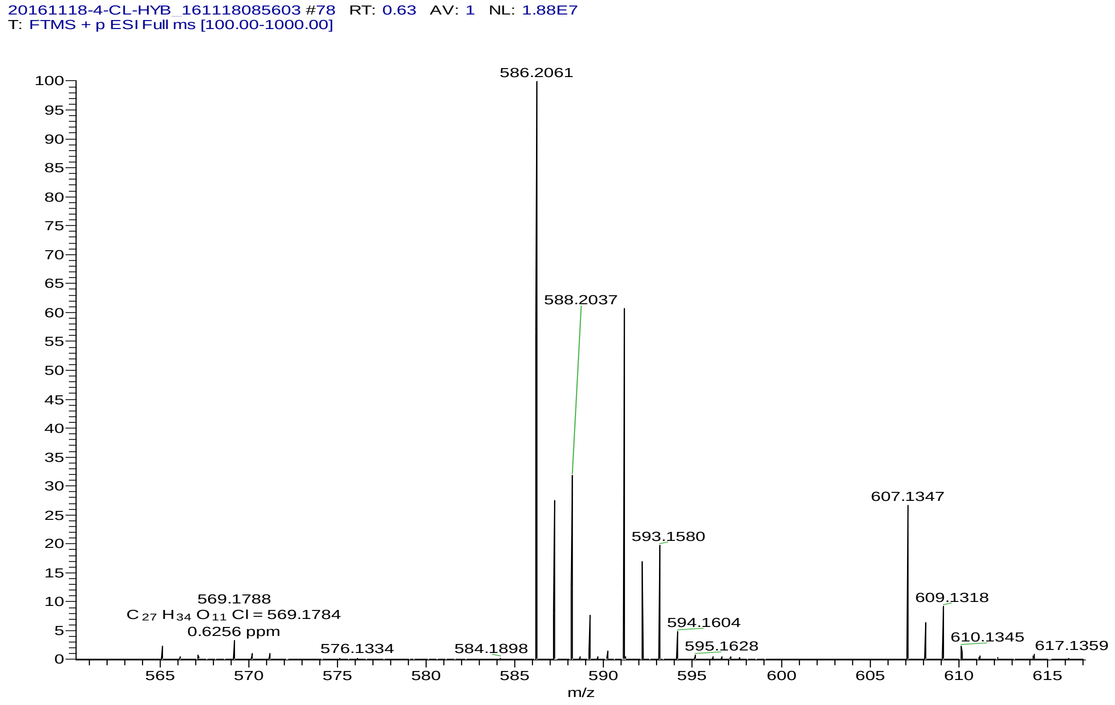

Figure S52. HRESIMS spectrum of compound **19**.

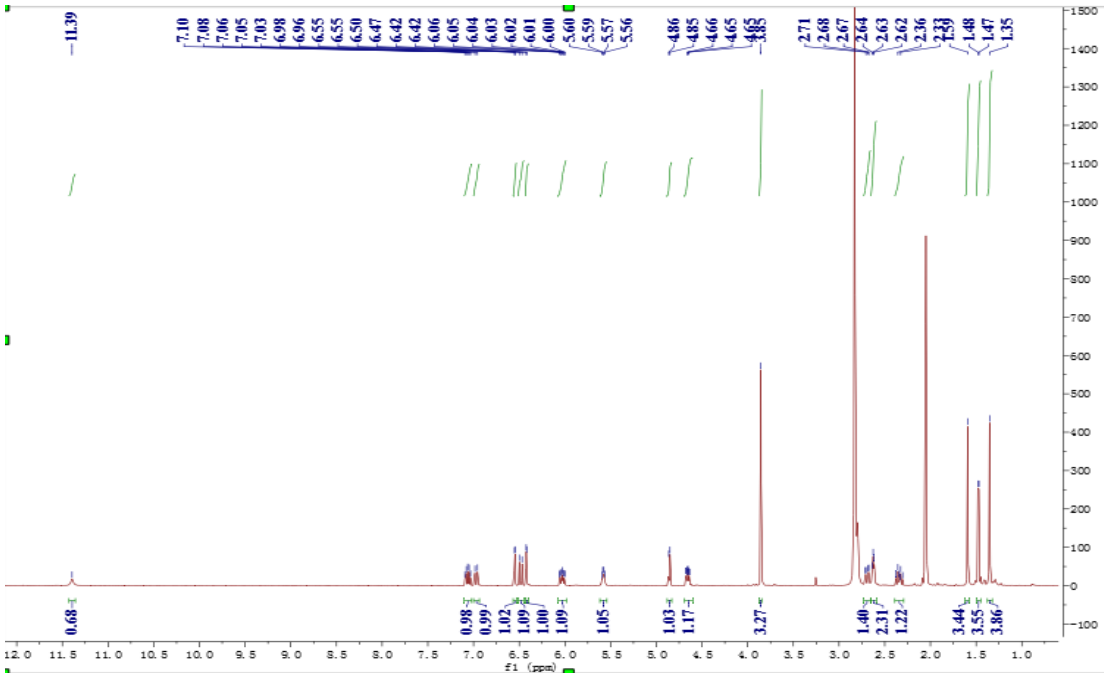

Figure S53. ^{1}H NMR (500 MHz, Acetone-d_6) spectrum of compound **20**.

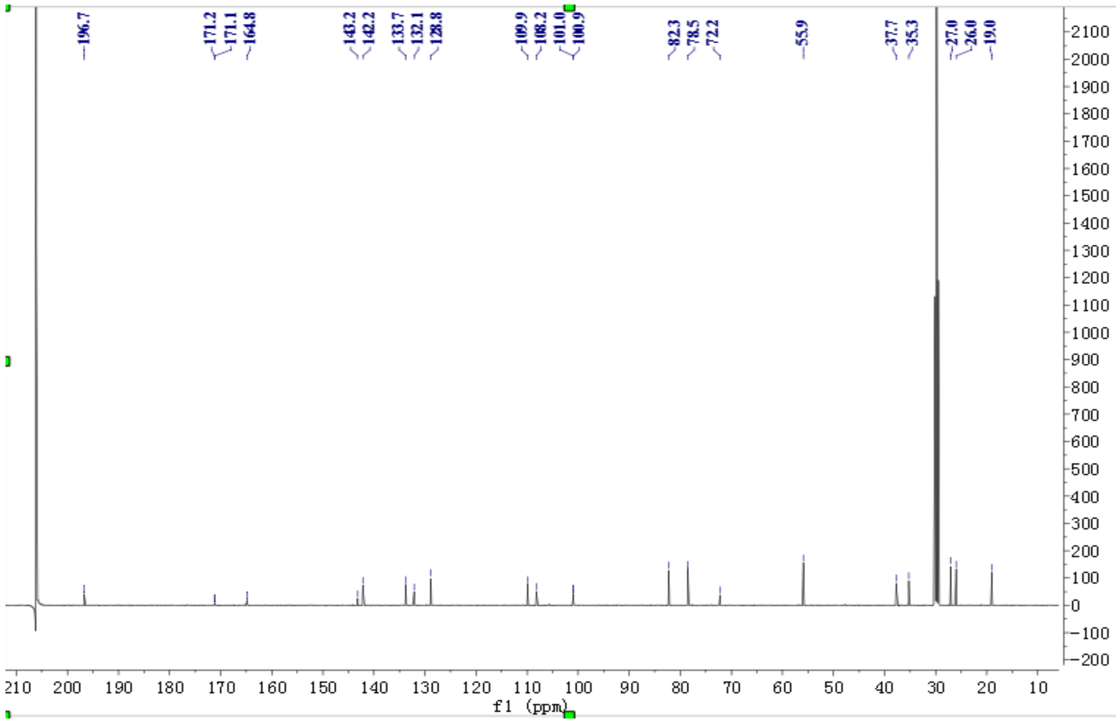

Figure S54. ^{13}C NMR (125 MHz, Acetone-d_6) spectrum of compound **20**.

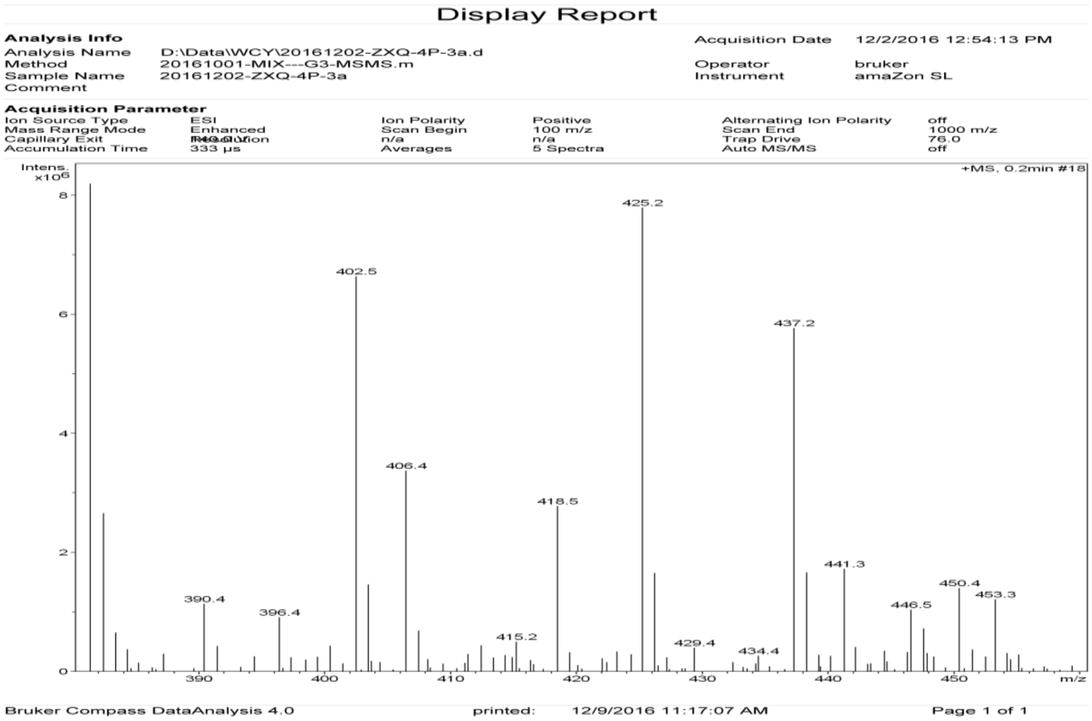

Figure S55. ESIMS spectrum of compound **20**.

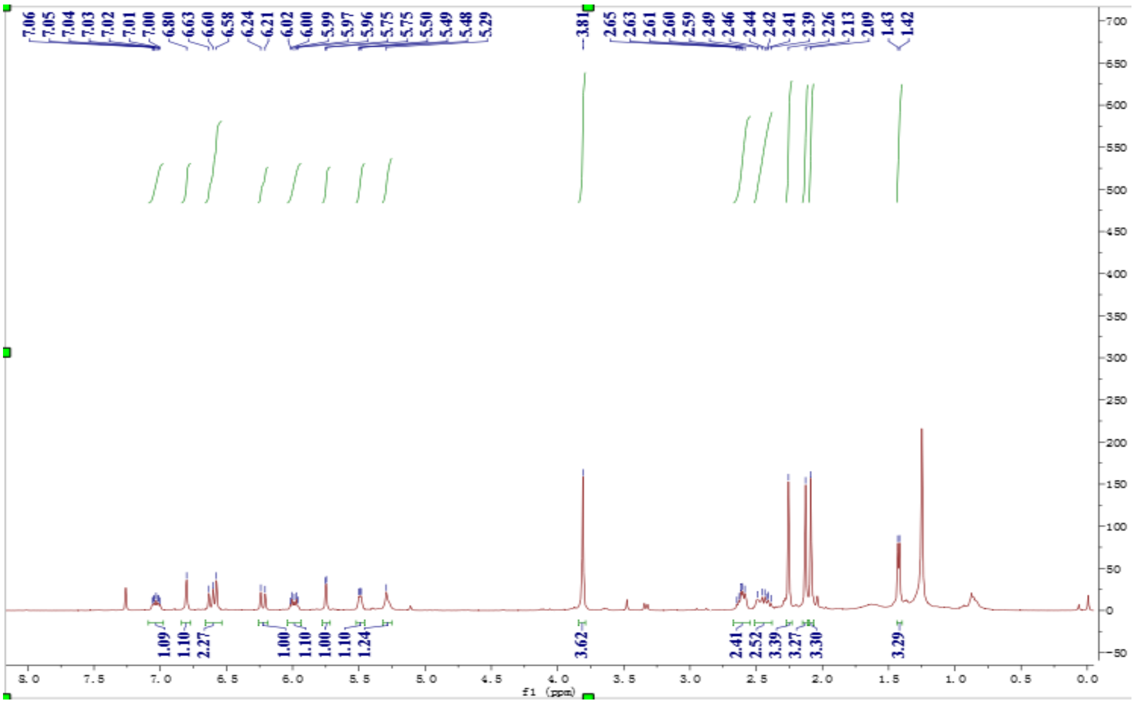

Figure S56. ^{1}H NMR (500 MHz, $CDCl_3$) spectrum of compound **21**.

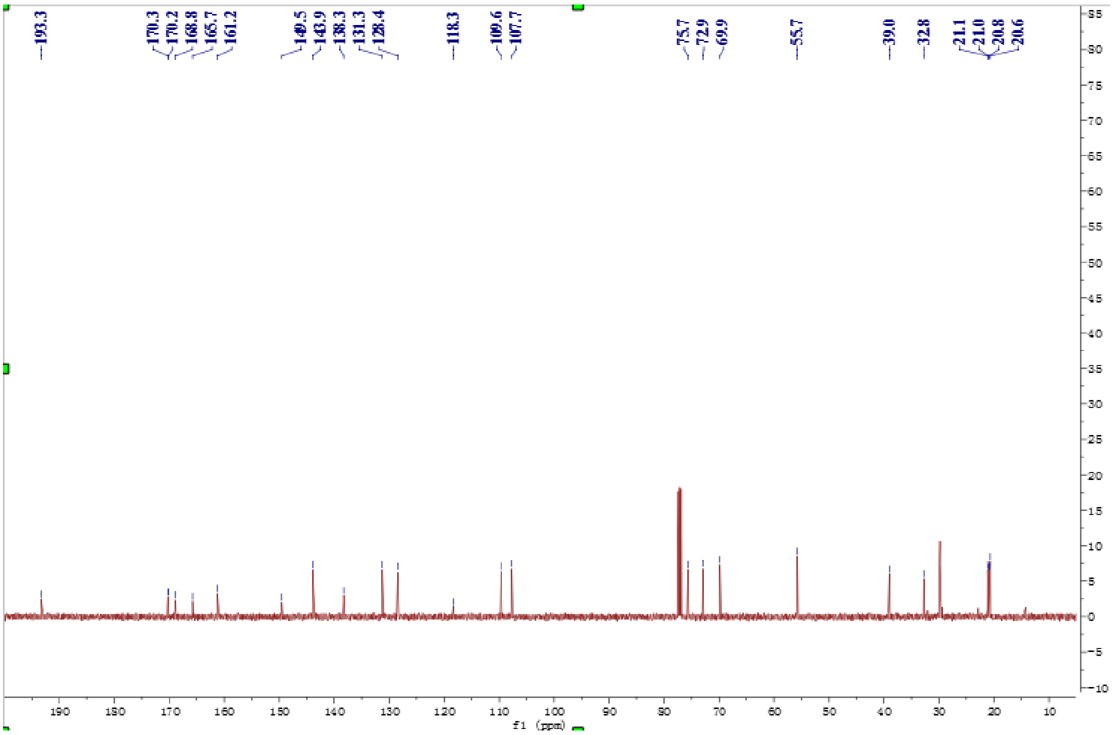

Figure S57. ^{13}C NMR (125 MHz, $CDCl_3$) spectrum of compound **21**.

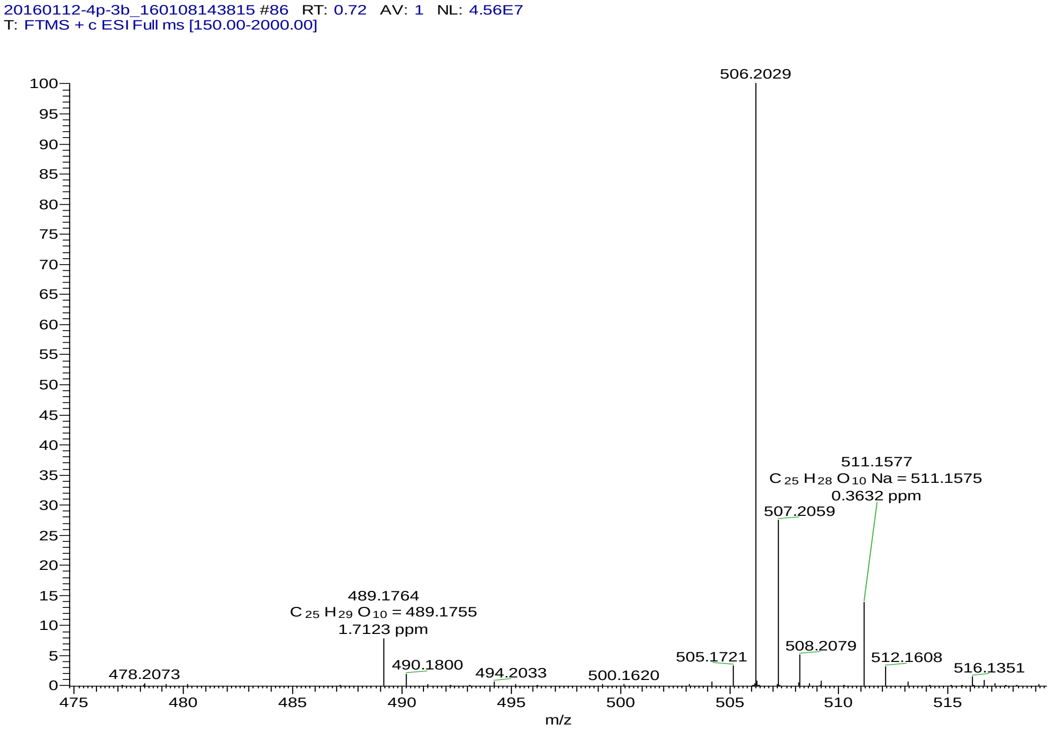

Figure S58. HRESIMS spectrum of compound **21**.

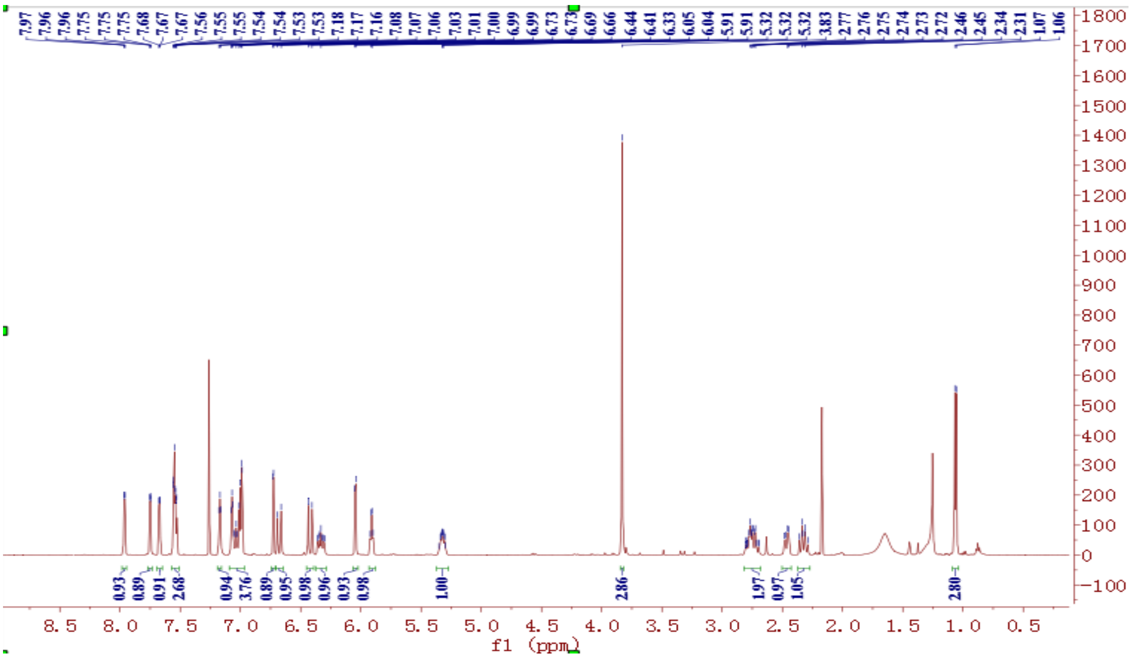

Figure S59. ^{1}H NMR (500 MHz, $CDCl_3$) spectrum of compound **22**.

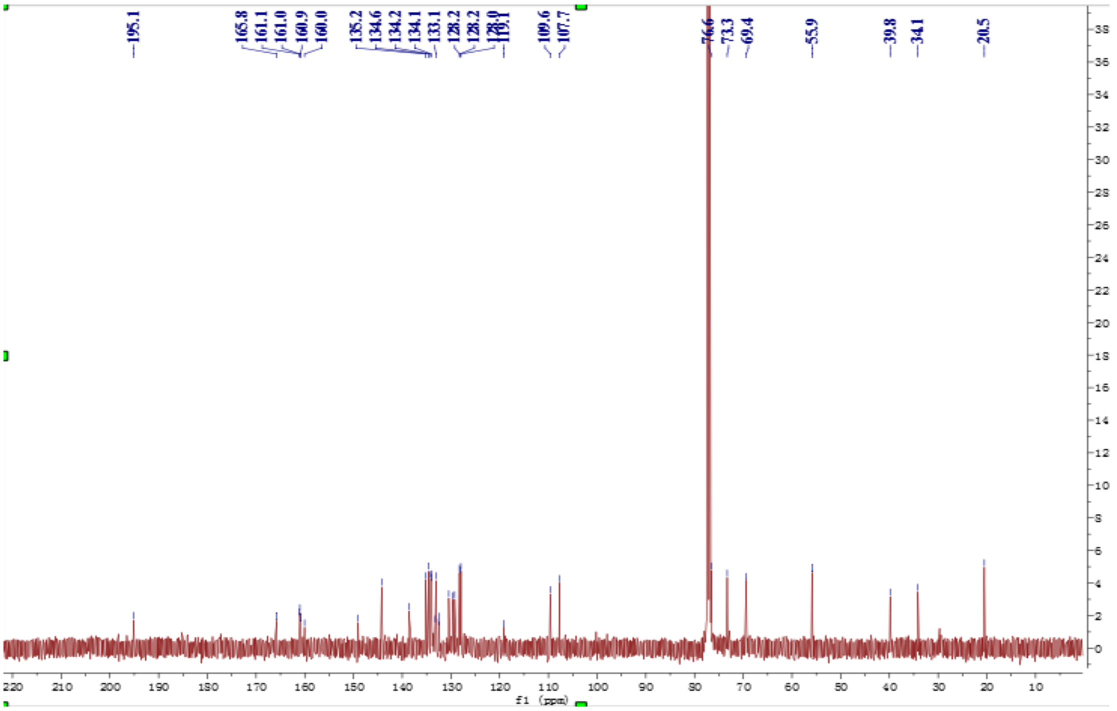

Figure S60. ^{13}C NMR (125 MHz, $CDCl_3$) spectrum of compound **22**.

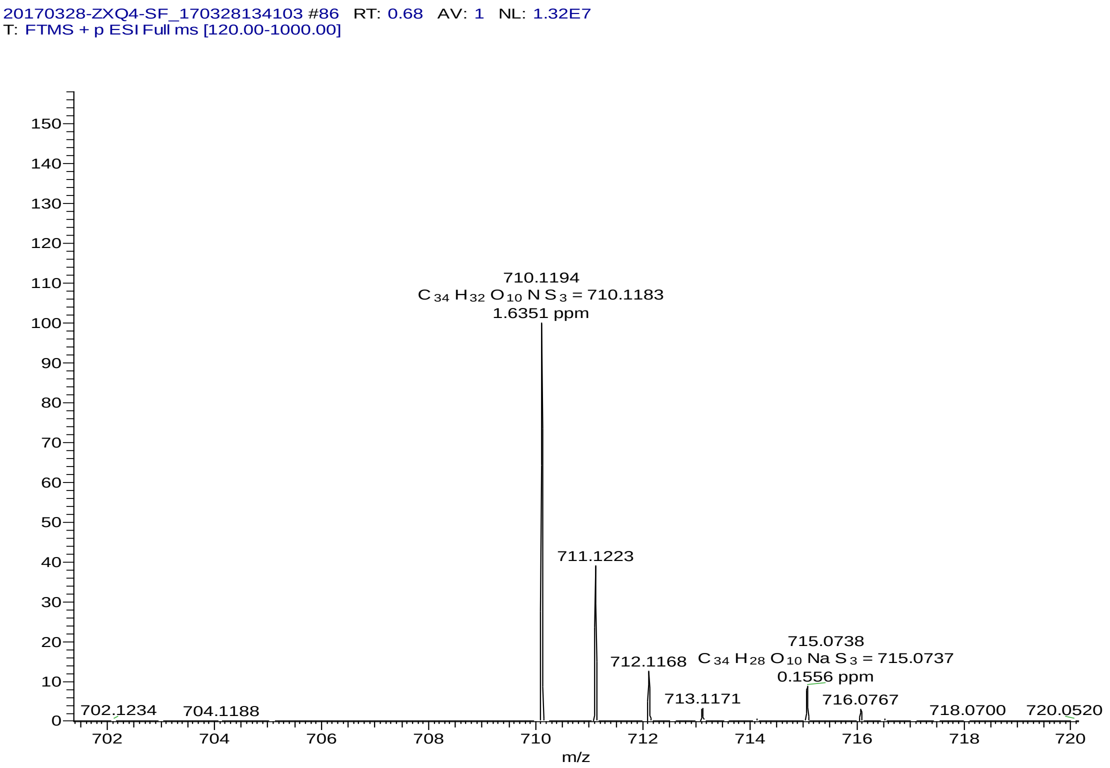

Figure S61. HRESIMS spectrum of compound **22**.

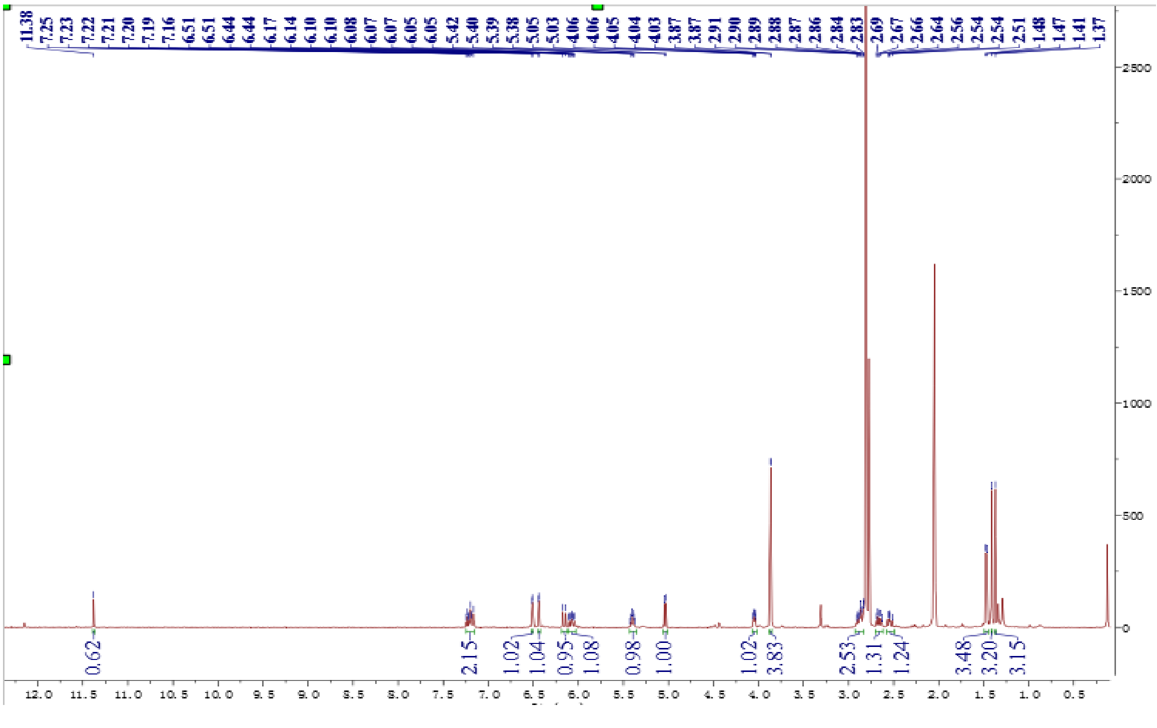

Figure S62. ^{1}H NMR (500 MHz, Acetone-d_6) spectrum of compound **23**.

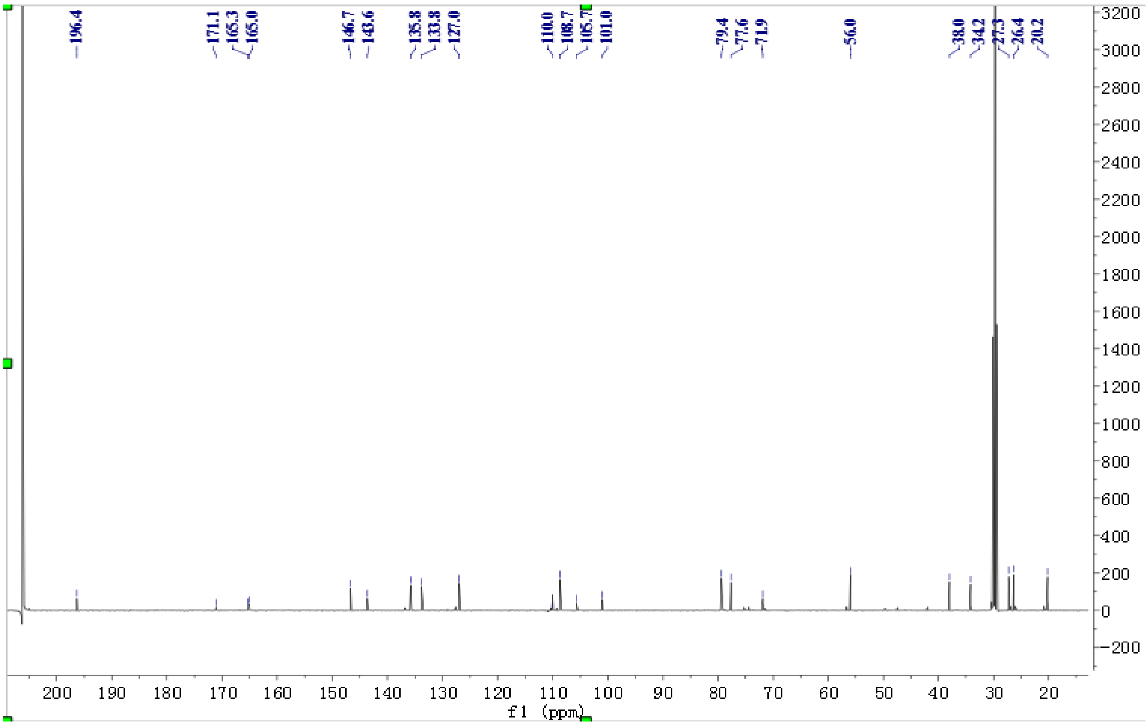

Figure S63. ^{13}C NMR (125 MHz, Acetone-d_6) spectrum of compound **23**.

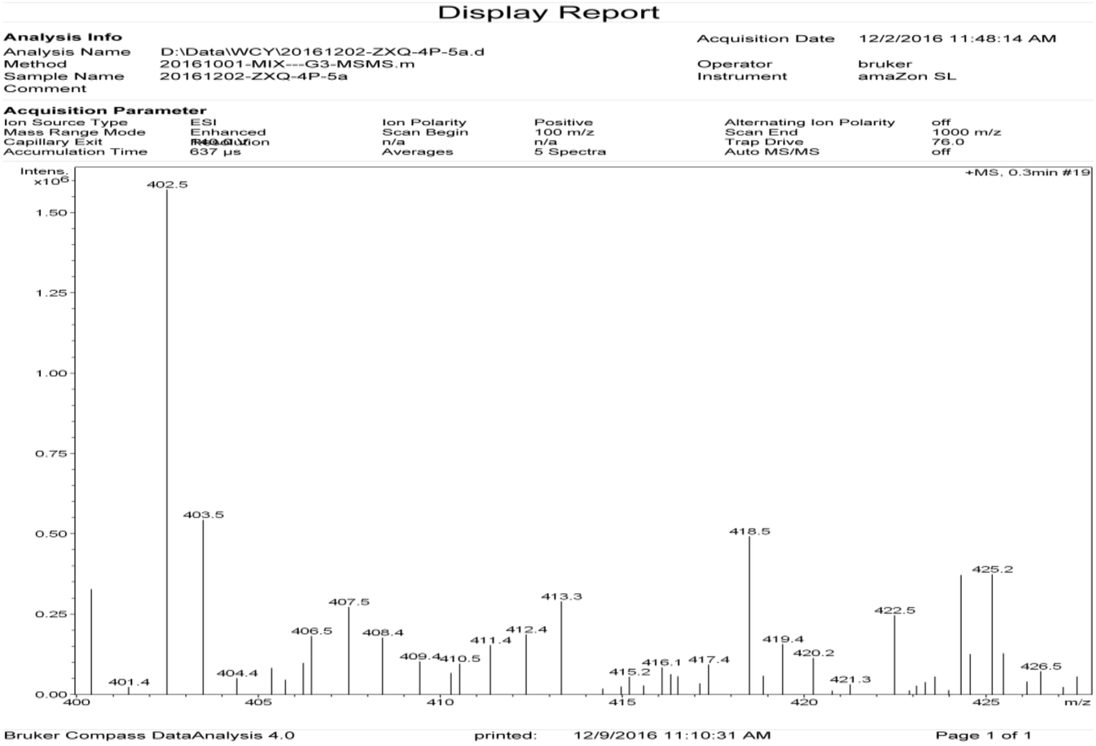

Figure S64. ESIMS spectrum of compound **23**.

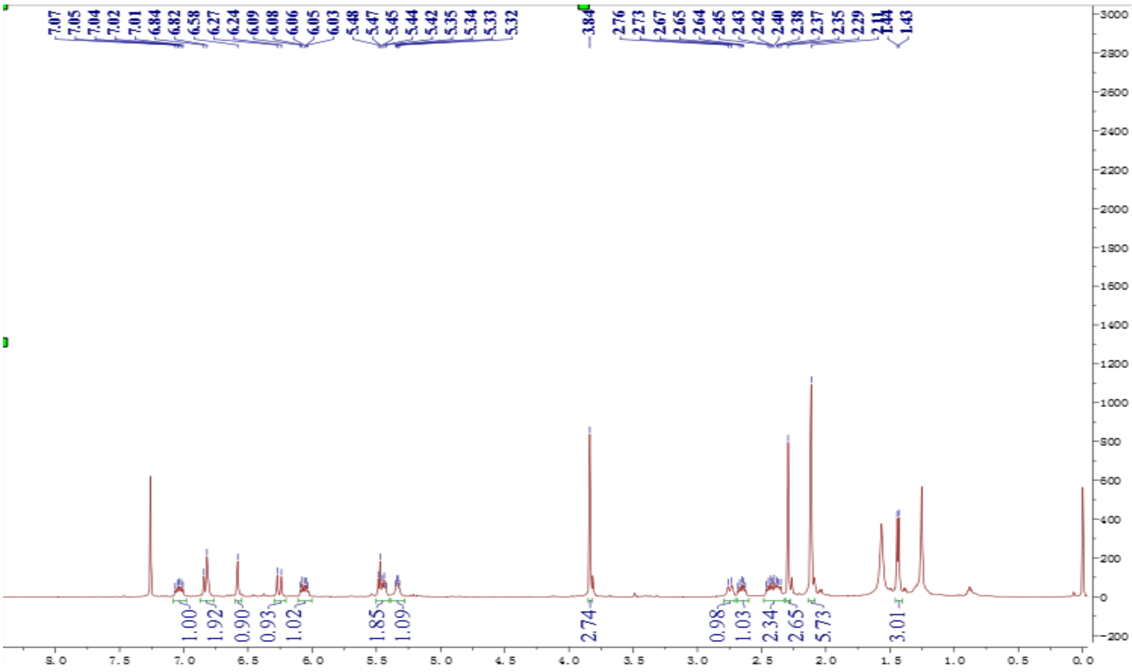

Figure S65. ^{1}H NMR (500 MHz, $CDCl_3$) spectrum of compound **24**.

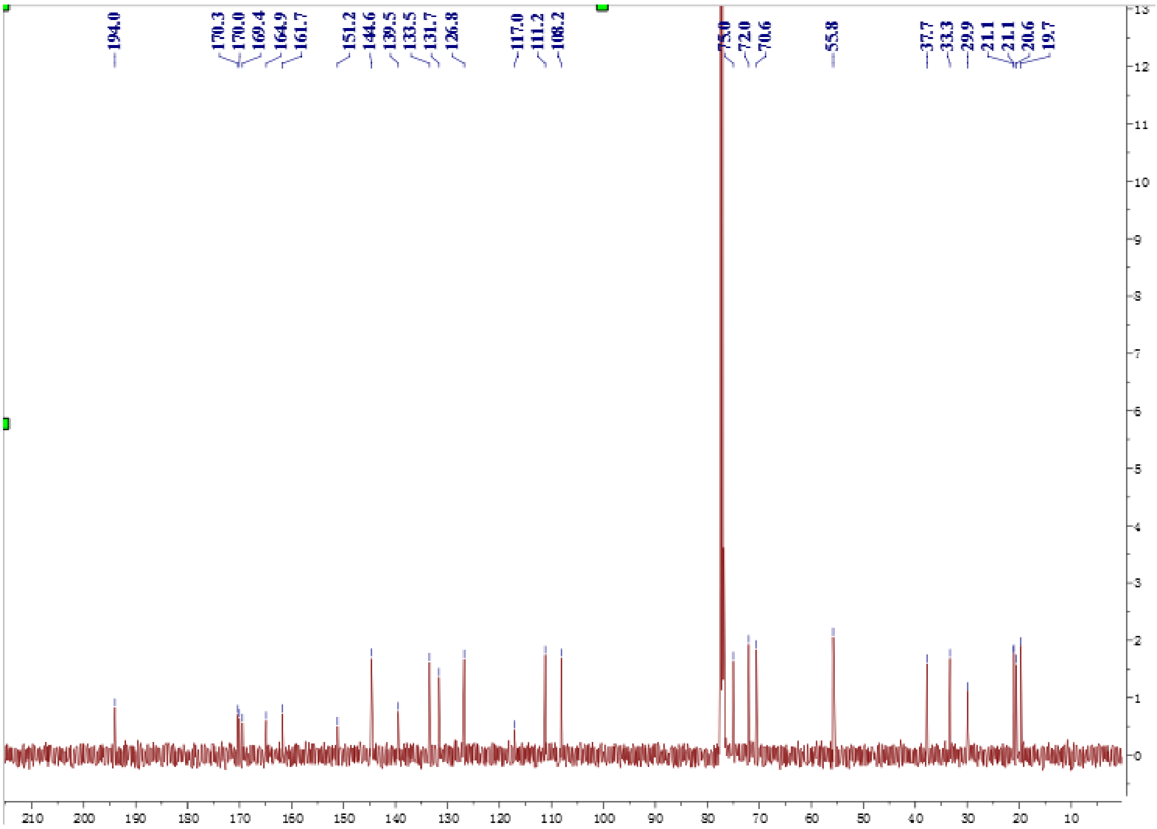

Figure S66. ^{13}C NMR (125 MHz, $CDCl_3$) spectrum of compound **24**.

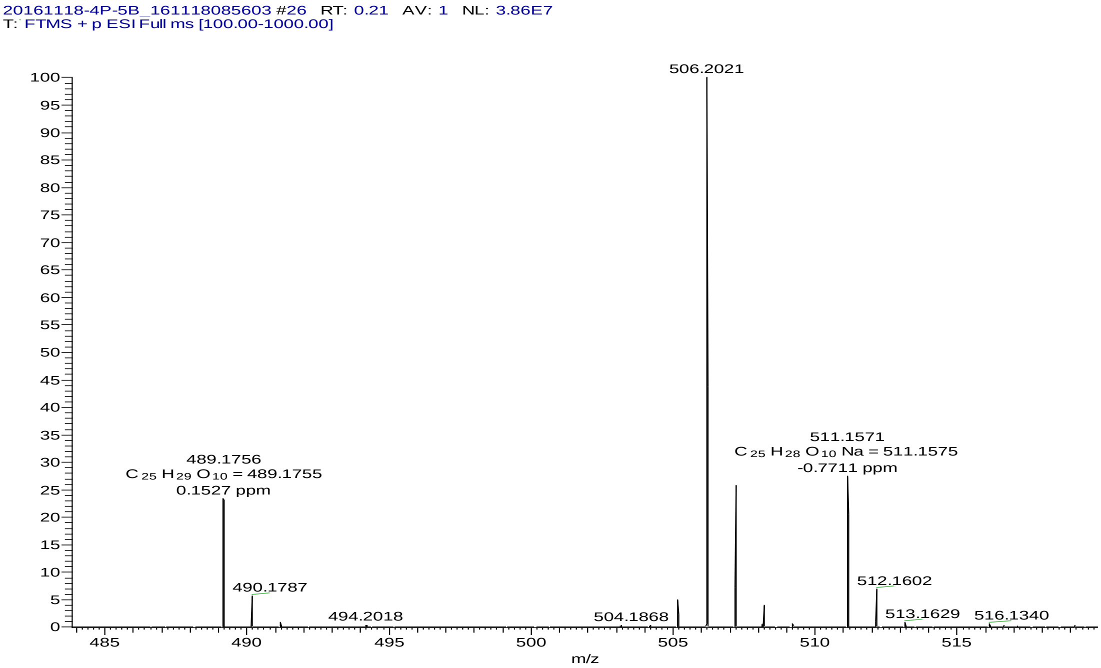

Figure S67. HRESIMS spectrum of compound **24**.

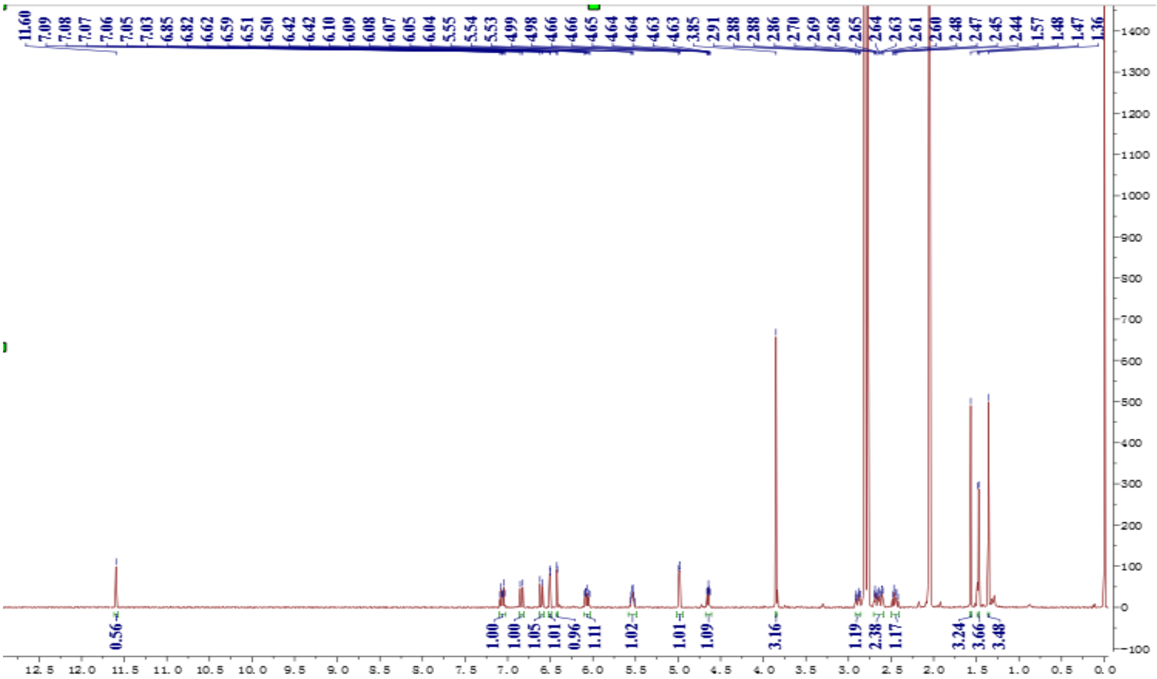

Figure S68. ^{1}H NMR (500 MHz, Acetone-d_6) spectrum of compound **25**.

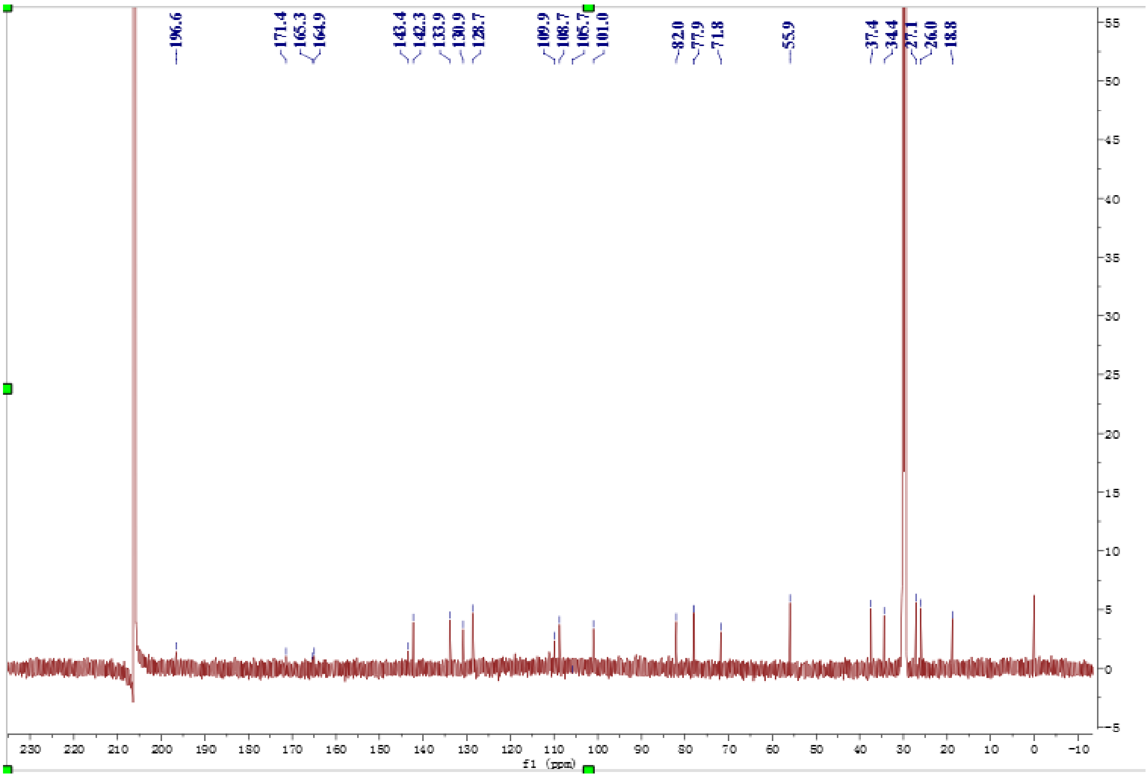

Figure S69. ^{13}C NMR (125 MHz, Acetone-d_6) spectrum of compound **25**.

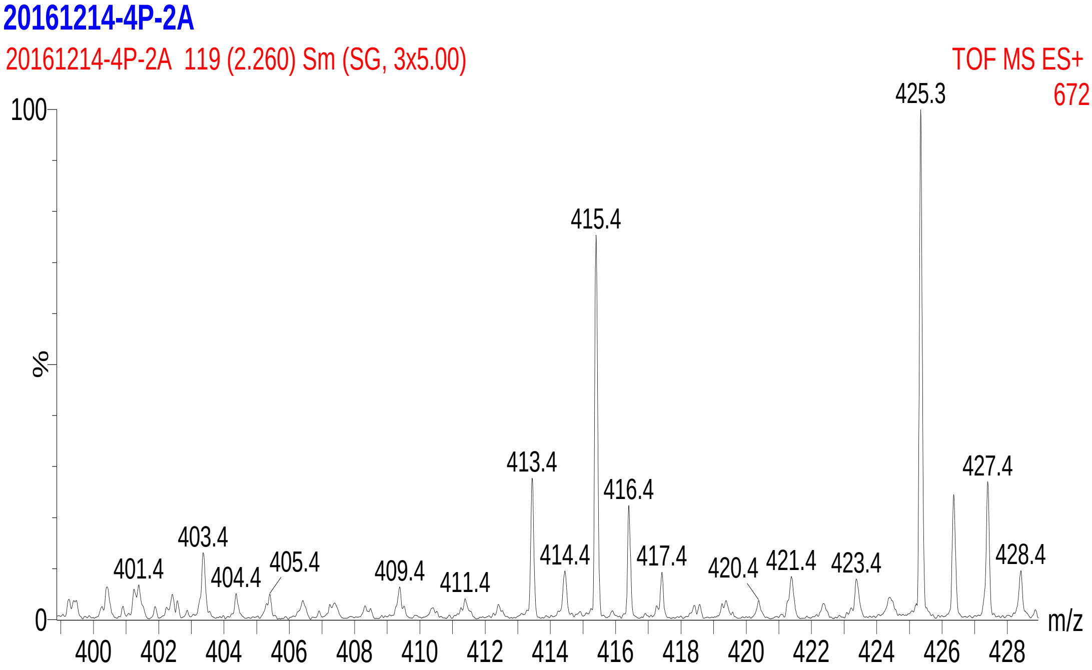

Figure S70. ESIMS spectrum of compound **25**.

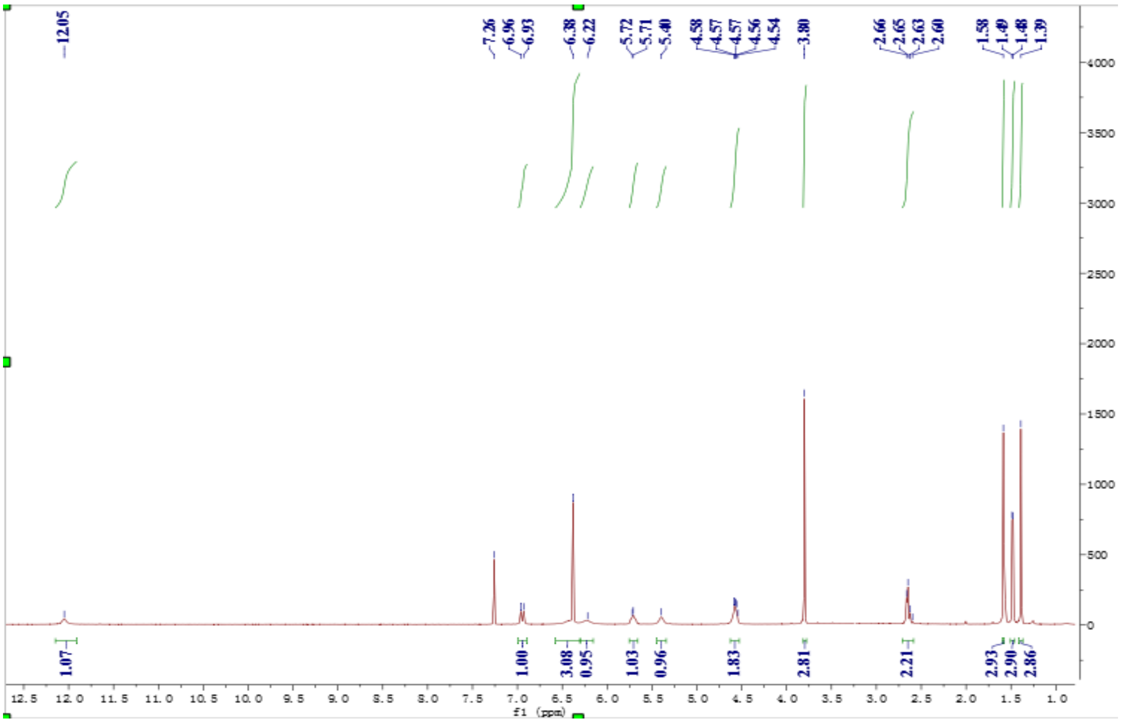

Figure S71. ^{1}H NMR (500 MHz, $CDCl_3$) spectrum of compound **26**.

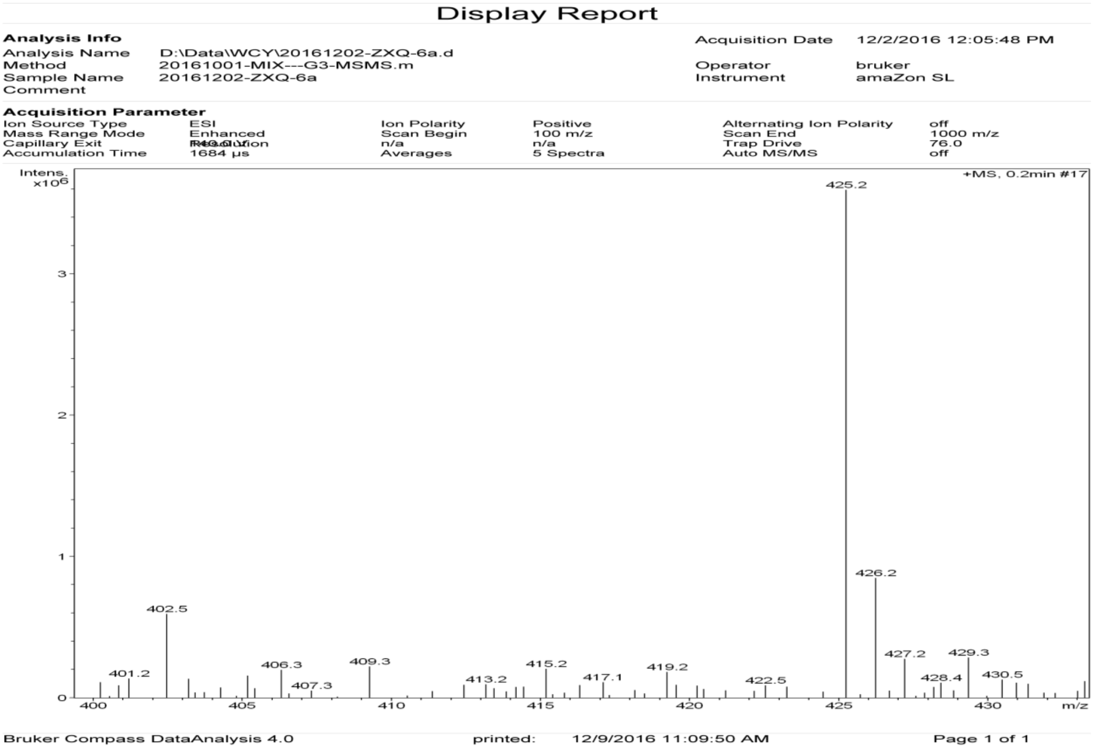

Figure S72. ESIMS spectrum of compound **26**.

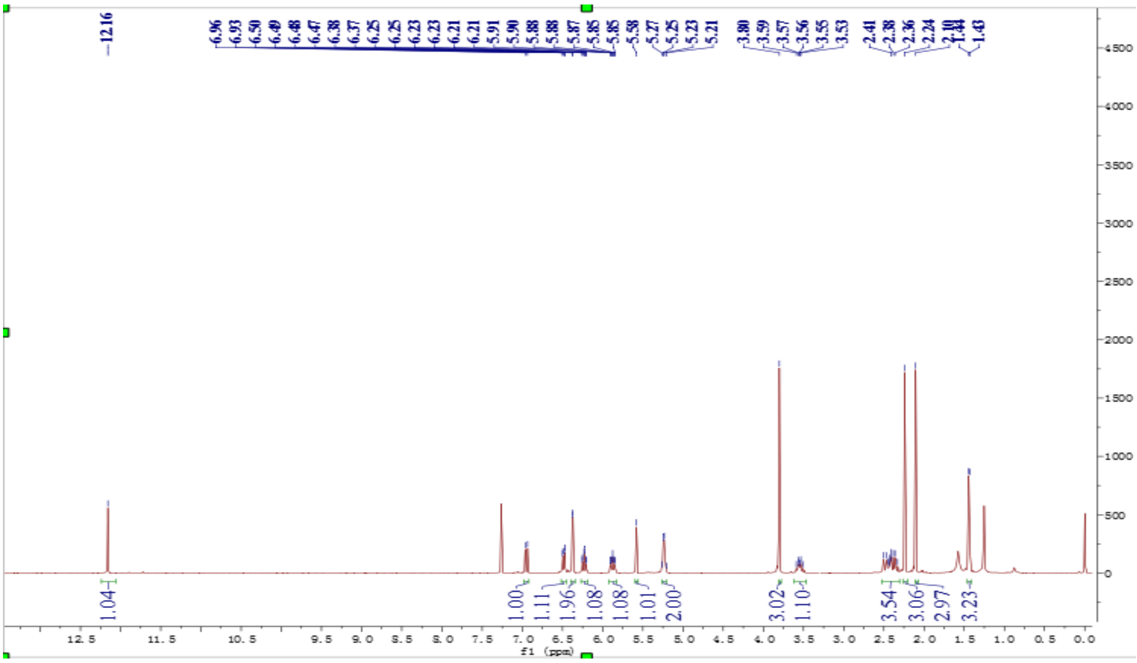

Figure S73. ^{1}H NMR (500 MHz, $CDCl_3$) spectrum of compound **27**.

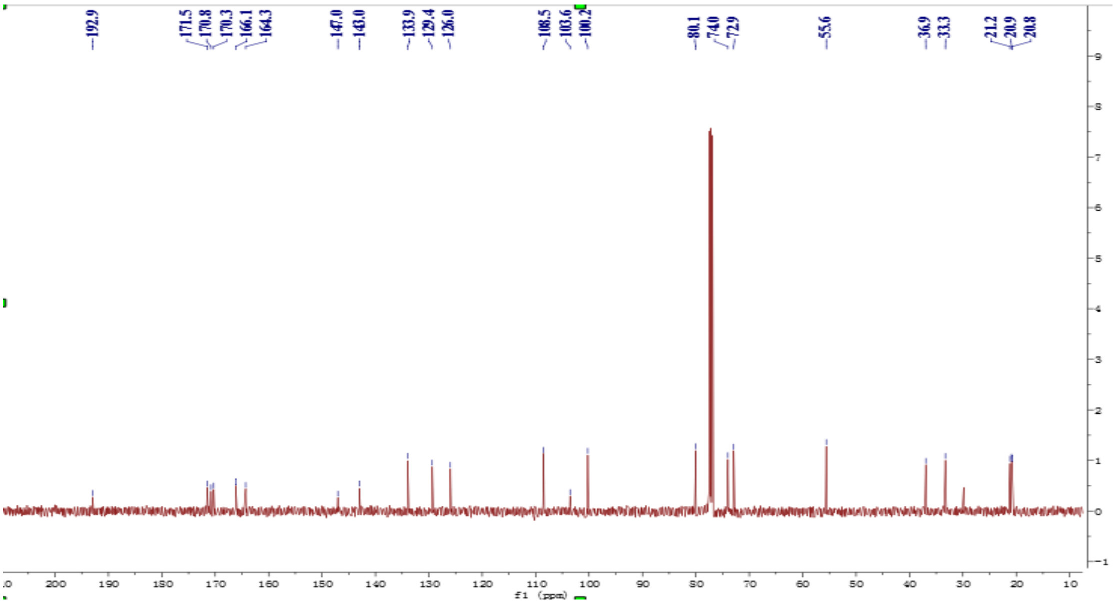

Figure S74. ^{13}C NMR (125 MHz, $CDCl_3$) spectrum of compound **27**.

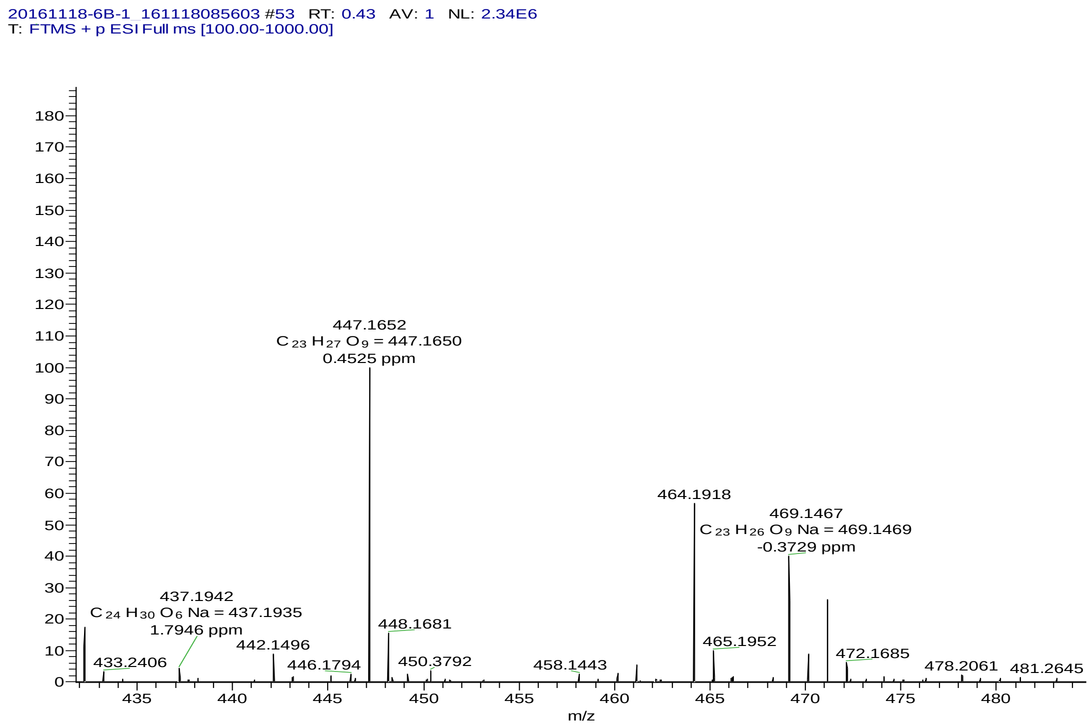

Figure S75. HRESIMS spectrum of compound **27**.

References

1. Liu, Q. A. *et al*. Antifouling and fungicidal resorcylic acid lactones from the sea anemone-derived fungus *Cochliobolus lunatus*. *J. Agric. Food Chem.* **62**, 3183–3191 (2014).

2. Shao, C. L. *et al*. Potent antifouling resorcylic acid lactones from the gorgonian-derived fungus *Cochliobolus lunatus*. *J. Nat. Prod.* **74**, 629–633 (2011).
